# Supplementary material for: Adherence to IMCI guidelines for key symptoms in Ethiopian children: A 2021–2022 national service provision survey
Source: PLoS One. 2024 Oct 16;19(10):e0312138. doi: 10.1371/journal.pone.0312138 (PMC11482708; doi:10.1371/journal.pone.0312138)
Supplement: Attachment — Submitted filename: PONE-D-24-03866_marked up with reviewer 1 comments.pdf [file pone.0312138.s001.pdf]

PLOS ONE

Adherence of Health Professionals to National IMCI Protocols: A Study on Assessing Three Key Symptoms in Sick Children in Ethiopia - Insights from the 2021 National Service Provision Survey

--Manuscript Draft--

**Manuscript Number:**	PONE-D-24-03866
**Article Type:**	Research Article
**Full Title:**	Adherence of Health Professionals to National IMCI Protocols: A Study on Assessing Three Key Symptoms in Sick Children in Ethiopia - Insights from the 2021 National Service Provision Survey
**Short Title:**	Adherence to IMCI protocols
**Corresponding Author:**	Abiyu Abadi Tareke<br>Amref Health Africa<br>GONDAR, ETHIOPIA
**Keywords:**	adherence, factor associated, IMCI protocol, Spatial analysis, Ethiopia
**Abstract:**	Background: The Health Services Provision Assessment in Ethiopia (SPA-ET) is a type of survey that generates data related to the availability and quality of health services in Ethiopia. Despite the presence of IMCI protocols in healthcare settings, there has been limited<br>exploration or assessment of how effectively and consistently health professionals are following these guidelines in the context of child healthcare. The objective of this study is to identify the factors of IMCI adherence and using geographical information systems (GIS) to pinpoint areas with elevated hesitancy rates and discern the factors driving these patterns.<br>Methods: The data utilized to analyze this study was based on information collected through Service provision assessment in Ethiopia (SPA-ET) survey conducted in 2021. A total of 788 sick children who had at least one of the three main symptoms of childhood illness: fever, cough, or diarrhea were included in this study. STATA version 16 was used to cross-tabulate and fit the models. To address the hierarchical nature of the health survey data, we employed multilevel logistic regression. Model comparison parameters, including BIC and AIC. We computed adjusted odds ratios with 95% confidence intervals, and statistical significance was determined at a significance level of $p < 0.05$.<br>Results: The rate adherence to the integrated management of childhood illness guideline (IMCI) is 33% (95% CI: 29.70%, 36.26%). Two-third of health professionals failed to assess the main symptoms of childhood sickness in accordance with IMCI recommendations. Noteworthy factors were associated with adherence rate to IMCI guideline encompass child's aged [AOR = 0.66, 95% CI: (0.45, 0.87)], health facility type [AOR = 2.61, 95% CI: (1.84, 3.37)], place of residency [AOR = 0.54, 95% CI: (0.38, 0.77)], and care provider's qualification [AOR = 1.71, 95% CI: (1.18, 2.48)]. Moreover, the primary cluster is situated in the west Oromia region, with a central focus at coordinates (7.982108 N, 36.203355 E) and extending to a radius of 78.28 km.<br><br>Conclusion: This study confirms a low adherence rate (33%) among health professionals in Ethiopia to the IMCI guideline for assessing the three main symptoms of sick children. The study identified child's age, types of facility, academic qualification and place of residency as crucial factors correlated with adherence rate. Furthermore, 5 secondary clusters (hotspot area) were identified using SaTScan software. To address the varying vaccine refusal, interventional plan needs to be based on academic qualification of care provider, types of health facility. Age of child and place of dwelling. Moreover, interventions to reduce non-adherence of IMCI guideline should be location-tailored based on identified hotspot areas in order to restore Guideline related adherence equality.
**Order of Authors:**	Abiyu Abadi Tareke
	Awoke Keleb
	Kaleab Mesfin Abera

Powered by Editorial Manager® and ProduXion Manager® from Aries Systems Corporation

	Natnael Kebede
	Endalkachew Mesfin
	Aznamariam Ayres
	Yawkal Tsega
	Abel Endawkie
	Shimels Derso Kebede
	Eyob Tilahun Abeje
	Ermias Bekele
	Chala Daba
	Lakew Asmare
	Fekade Demeke Bayou
	Mastewal Arefaynie
	Asnakew Molla Mekonen
**Opposed Reviewers:**	
**Additional Information:**	
**Question**	**Response**
**Financial Disclosure**<br><br>Enter a financial disclosure statement that describes the sources of funding for the work included in this submission. Review the submission guidelines for detailed requirements. View published research articles from *PLOS ONE* for specific examples.<br><br>This statement is required for submission and **will appear in the published article** if the submission is accepted. Please make sure it is accurate.<br><br>**Funded studies**<br>Enter a statement with the following details:<br>• Initials of the authors who received each award<br>• Grant numbers awarded to each author<br>• The full name of each funder<br>• URL of each funder website<br>• Did the sponsors or funders play any role in the study design, data collection and analysis, decision to publish, or preparation of the manuscript?<br><br>Did you receive funding for this work?	The author(s) received no specific funding for this work.

**Competing Interests**<br><br>Use the instructions below to enter a competing interest statement for this submission. On behalf of all authors, disclose any competing interests that could be perceived to bias this work—acknowledging all financial support and any other relevant financial or non-financial competing interests.<br><br>This statement is required for submission and **will appear in the published article** if the submission is accepted. Please make sure it is accurate and that any funding sources listed in your Funding Information later in the submission form are also declared in your Financial Disclosure statement.<br><br>View published research articles from *PLOS ONE* for specific examples.<br><br>**NO authors have competing interests**<br><br>Enter: *The authors have declared that no competing interests exist.*<br><br>**Authors with competing interests**<br><br>Enter competing interest details beginning with this statement:<br><br>*I have read the journal's policy and the authors of this manuscript have the following competing interests: [insert competing interests here]*<br><br>* typeset	The authors have declared that no competing interests exist
**Ethics Statement**<br><br>Enter an ethics statement for this submission. This statement is required if the study involved:<br><br>• Human participants<br>• Human specimens or tissue<br>• Vertebrate animals or cephalopods	N/A

Powered by Editorial Manager® and ProduXion Manager® from Aries Systems Corporation

• Vertebrate embryos or tissues<br>• Field research<br><br>Write "N/A" if the submission does not require an ethics statement.<br><br>General guidance is provided below. Consult the submission guidelines for detailed instructions. **Make sure that all information entered here is included in the Methods section of the manuscript.**	

Powered by Editorial Manager® and ProduXion Manager® from Aries Systems Corporation

**Format for specific study types**<br><br>**Human Subject Research (involving human participants and/or tissue)**<br>• Give the name of the institutional review board or ethics committee that approved the study<br>• Include the approval number and/or a statement indicating approval of this research<br>• Indicate the form of consent obtained (written/oral) or the reason that consent was not obtained (e.g. the data were analyzed anonymously)<br><br>**Animal Research (involving vertebrate animals, embryos or tissues)**<br>• Provide the name of the Institutional Animal Care and Use Committee (IACUC) or other relevant ethics board that reviewed the study protocol, and indicate whether they approved this research or granted a formal waiver of ethical approval<br>• Include an approval number if one was obtained<br>• If the study involved *non-human primates*, add *additional details* about animal welfare and steps taken to ameliorate suffering<br>• If anesthesia, euthanasia, or any kind of animal sacrifice is part of the study, include briefly which substances and/or methods were applied<br><br>**Field Research**<br><br>Include the following details if this study involves the collection of plant, animal, or other materials from a natural setting:<br>• Field permit number<br>• Name of the institution or relevant body that granted permission	
**Data Availability**<br><br>Authors are required to make all data underlying the findings described fully available, without restriction, and from the time of publication. PLOS allows rare exceptions to address legal and ethical concerns. See the PLOS Data Policy and FAQ for detailed information.	Yes - all data are fully available without restriction

A Data Availability Statement describing where the data can be found is required at submission. Your answers to this question constitute the Data Availability Statement and **will be published in the article**, if accepted.<br><br>**Important:** Stating 'data available on request from the author' is not sufficient. If your data are only available upon request, select 'No' for the first question and explain your exceptional situation in the text box.<br><br>Do the authors confirm that all data underlying the findings described in their manuscript are fully available without restriction?	
**Describe where the data may be found in full sentences. If you are copying our sample text, replace any instances of XXX with the appropriate details.**<br><br>• If the data are **held or will be held in a public repository**, include URLs, accession numbers or DOIs. If this information will only be available after acceptance, indicate this by ticking the box below. For example: *All XXX files are available from the XXX database (accession number(s) XXX, XXX.*).<br>• If the data are all contained **within the manuscript and/or Supporting Information files**, enter the following: *All relevant data are within the manuscript and its Supporting Information files.*<br>• If neither of these applies but you are able to provide **details of access elsewhere**, with or without limitations, please do so. For example:<br><br>*Data cannot be shared publicly because of [XXX]. Data are available from the XXX Institutional Data Access / Ethics Committee (contact via XXX) for researchers who meet the criteria for access to confidential data.*<br><br>*The data underlying the results presented in the study are available from (include the name of the third party*	The datasets and materials are available at https://www.dhsprogram.com. The minimal data used for this study are available from the corresponding author on reasonable request.

Powered by Editorial Manager® and ProduXion Manager® from Aries Systems Corporation

and contact information or URL).<br>• This text is appropriate if the data are owned by a third party and authors do not have permission to share the data.<br><br>* typeset	
Additional data availability information:	

Powered by Editorial Manager® and ProduXion Manager® from Aries Systems Corporation

To: **plos one Editors**

Date: January 29, 2024

Subject: Manuscript Submission for Consideration - [**Adherence of Health Professionals to National IMCI Protocols: A Study on Assessing Three Key Symptoms in Sick Children in Ethiopia - Insights from the 2021 National Service Provision Survey**]

We are pleased to submitting the research article for consideration for publication in ***plos one.*** This research investigates the crucial issue of health professionals' adherence to National Integrated Management of Childhood Illness (IMCI) protocols during the assessment of three main symptoms in sick children. Using data from the 2021 National Service Provision Survey in Ethiopia, our study provides valuable insights into the current practices and challenges in adhering to IMCI protocols.

The key contributions of our research include identification of factors associated with adherence of IMCI protocols during the assessment of the three main symptoms of childhood illness and hotspot areas with high number of health professionals who are failed to follow the recommendations of IMCI guidelines. We believe that our work aligns well with the scope and mission of plos one.

We consider ***plos one*** Journal for publication because it is peer reviewed journal with high impact factor and fastest rate of processing. This manuscript is my original work, not published before and is not under consideration for publication in any Journal elsewhere. I have no conflicts of interest to disclose.

With best regards,

Abiyu Abadi Tareke
Corresponding author

Email: abiyu20010@gmail.com

Manuscript Click here to access/download;Manuscript;Manuscript.docx

Adherence of Health Professionals to National IMCI Protocols: A Study on Assessing Three Key Symptoms in Sick Children in Ethiopia - Insights from the 2021 National Service Provision Survey

[1]Abiyu Abadi Tareke*, [2]Awoke Keleb, [3]Kaleab Mesfin Abera, [4]Natnael Kebede, [5]Endalkachew Mesfin, [6]Aznamariam Ayres, [7]Yawkal Tsega, [8]Abel Endawkie, [9]Shimels Derso Kebede, [10]Eyob Tilahun Abeje, [11]Ermias Bekele, [12]Chala Daba, [13]Lakew Asmare, [14]Fekade Demeke Bayou, [15]Mastewal Arefaynie & [16]Asnakew Molla Mekonen

[1]Amref health in Africa, COVID-19 vaccine/EPI technical assistant at West Gondar zonal health department, Gondar, Ethiopia

[2&12]Department of Environmental Health College of Medicine and Health Sciences, Wollo University, Dessie, Ethiopia

[4]Department of Health Promotion, School of Public Health, College of Medicine and Health Sciences, Wollo University, Dessie, Ethiopia

[3,5,7 &16]Department of Health System and Management, School of Public Health, College of Medicine and Health Sciences, Wollo University, Dessie, Ethiopia

[9&11]Department of Health Informatics, School of Public Health, College of Medicine and Health Sciences, Wollo University, Dessie, Ethiopia

[6,8,10, 13 & 14]Department of Epidemiology and Biostatistics School of Public Health College of Medicine and Health Science Wollo University, Dessie, Ethiopia.

[15]Department of Reproductive and Family Health, School of Public Health, College of Medicine and Health Sciences, Wollo University, Dessie, Ethiopia

Abiyu Abadi Tareke

Corresponding author (abiyu20010@gmail.com)

Abstract

Background: The Health Services Provision Assessment in Ethiopia (SPA-ET) is a type of survey that generates data related to the availability and quality of health services in Ethiopia. Despite the presence of IMCI protocols in healthcare settings, there has been limited exploration or assessment of how effectively and consistently health professionals are following these guidelines in the context of child healthcare. The objective of this study is to identify the factors of IMCI adherence and using geographical information systems (GIS) to pinpoint areas with elevated hesitancy rates and discern the factors driving these patterns.

Methods: The data utilized to analyze this study was based on information collected through Service provision assessment in Ethiopia (SPA-ET) survey conducted in 2021. A total of 788 sick children who had at least one of the three main symptoms of childhood illness: fever, cough, or diarrhea were included in this study. STATA version 16 was used to cross-tabulate and fit the models. To address the hierarchical nature of the health survey data, we employed multilevel logistic regression. Model comparison parameters, including BIC and AIC. We computed adjusted odds ratios with 95% confidence intervals, and statistical significance was determined at a significance level of $p < 0.05$.

Results: The rate adherence to the integrated management of childhood illness guideline (IMCI) is 33% (95% CI: 29.70%, 36.26%). Two-third of health professionals failed to assess the main symptoms of childhood sickness in accordance with IMCI recommendations. Noteworthy factors were associated with adherence rate to IMCI guideline encompass child's

aged [AOR = 0.66, 95% CI: (0.45, 0.87)], health facility type [AOR = 2.61, 95% CI: (1.84, 3.37)], place of residency [AOR = 0.54, 95% CI: (0.38, 0.77)], and care provider's qualification [AOR = 1.71, 95% CI: (1.18, 2.48)]. Moreover, the primary cluster is situated in the west Oromia region, with a central focus at coordinates (7.982108 N, 36.203355 E) and extending to a radius of 78.28 km.

Conclusion: This study confirms a low adherence rate (33%) among health professionals in Ethiopia to the IMCI guideline for assessing the three main symptoms of sick children. The study identified child's age, types of facility, academic qualification and place of residency as crucial factors correlated with adherence rate. Furthermore, 5 secondary clusters (hotspot area) were identified using SaTScan software. To address the varying vaccine refusal, interventional plan needs to be based on academic qualification of care provider, types of health facility. Age of child and place of dwelling. Moreover, interventions to reduce non-adherence of IMCI guideline should be location-tailored based on identified hotspot areas in order to restore Guideline related adherence equality.

Key words: adherence, factor associated, IMCI protocol, Spatial analysis, Ethiopia

Introduction

The Ethiopian health service provision (E-SPA) is a holistic survey mainly conducted to assess the quality and availability of health services at innumerable healthcare facilities (hospitals, health centers and other private health facilities)(1). The survey brings information regarding the performance of health systems, weakness and strength of the healthcare delivery system, and areas of enhancement. SPA also assesses the quality of healthcare by evaluating adherence

of health workers to clinical guidelines such as integrated management of child illness (IMCI), patient management and infection prevention(2).

To assist health workers in effectively diagnosing and treating sick children in laboratories and medical equipment limited countries like Ethiopia, World Health Organization (WHO) and the United Nations Children's Fund (UNICEF) introduced IMCI guideline since 1995(3-6). IMCI has countless potential to advance healthcare providers performance(7, 8) and quality care for sick children(3). In Ethiopia, a country with unique healthcare challenges(9), the national IMCI protocols have been instrumental in guiding health professionals in the assessment and management of these critical symptoms(8).

This guideline starts by identification of key symptoms of sick children. Among these, the guideline mainly assesses three symptoms which are indicative of a range of common childhood illnesses, including pneumonia, diarrhea, respiratory infections, and other potentially severe conditions. Those symptoms are general indicators of danger signs which may require hospitalizations(10). Childhood illnesses, mainly those manifesting through vital symptoms such as fever, diarrhea, and cough/difficulty of breathing, contribute significantly to the global burden of morbidity and mortality among children under the age of five.

In 2015, a staggering reality persisted approximately 16,000 children under the age of five continued to lose their lives every day due to preventable causes: Pneumonia, diarrhea, and malaria (11). Current data in Ethiopia suggests that neonatal mortality is at 29 per 1000 live births, infant mortality is at 48 per 1000 live births, and under-5 mortality is at 67 per 1000 live births(12). Surveys conducted in health facilities across three regional states in Ethiopia—Amhara, Oromia, and Southern Nations and Nationalities Peoples' (SNNP)—revealed limited coverage of IMCI in these areas. Amhara exhibited a 20% IMCI coverage, Oromia recorded a

4% coverage, and the South Nations Nationalities People (SNNP) Region reported a 25% coverage(13). Notably, the percentage of trained targeted health workers in these three regions was even lower than the IMCI coverage.

While IMCI has been implemented in certain health facilities across Ethiopia, there remains a significant gap in understanding the adherence status of health professionals to this guideline. Despite the presence of IMCI protocols in healthcare settings, there has been limited exploration or assessment of how effectively and consistently health professionals are following these guidelines in the context of child healthcare. Our comprehensive review of existing literature has not revealed any studies or investigations concerning the adherence of health workers to IMCI protocols in Ethiopia.

This study is the first study to measure the adherence rate of health professionals toward the recommendation of IMCI guideline during the assessment of the three main symptoms of childhood illness. The comprehensive analysis of the most recent data allows for a robust evaluation of the situation of healthcare workers in following the protocols of IMCI in Ethiopia. This research goes beyond simply assessing the national-level scenario and explores into the spatial pattern of IMCI adherence. By employing a potent tool called SaTScanTM software, this study evaluates hotspots of non-adherent health professionals. Identifying hotspot areas could be beneficial for both national and local health decision makers, which allows for location targeted interventions in specific geographic areas and helps for better health resource allocation.

Therefore, this study plays a vital role in unmasking IMCI adherence related problems in Ethiopia. By utilizing national representative data from the latest survey and incorporating advanced statistical techniques, our research provides an accurate assessment of the spatial

variation and factors linked to IMCI adherence. The findings serve as a valuable resource for policymakers, aiding them in addressing the specific needs of different regions within the country. Through this study, we aim to contribute significantly to the ongoing efforts in maximizing IMCI guideline implementation coverage in Ethiopia.

Methods and materials

Study design and period

Study area

Ethiopia, the second-most populous country in Africa, boasts a diverse administrative structure that ranges from the highest level of the region to the lowest level of Kebele. Ethiopia is bordered by Sudan and South Sudan on the west, Eritrea and Djibouti on the northeast, Somalia on the east and southeast, and Kenya on the south. The distribution of population between rural and urban communities is unequal. The total fertility rate of the country is 4.6 births per woman. The corresponding crude birth rate was 32 per 1,000 in 2016. The average household size is 4.6, and the population is projected to reach 122.3 million by 2030(2).

The health system of Ethiopia is composed of a three-tiered, service-delivery structure, with primary (This level consists of health posts, health centers, and primary hospitals), secondary (This level consists of general hospitals and is supposed to provide similar services to those of primary hospitals and serve, on average, one million people), and tertiary-level health care (This is the third tier in the Ethiopian health care system and consists of a specialized hospital that covers a population of approximately 5 million). The health care tier system includes both

public and private health facilities. The private health facilities include clinics (all levels), diagnostic centers, pharmacies, and drug stores.

Ethiopia comprises eleven regions, namely Tigray, Oromia, Afar, Sidama, Afar, Amhara, Benishangul Gumuz, south west Ethiopia, SNNP, Gambella, and Somali (Figure 1). Additionally, it encompasses two administrative cities, namely Dire-Dawa and Addis Ababa. Each region is further divided into zones, zones into woredas, and finally, woredas and the last administrative structure known as Kebeles (Figure 1).

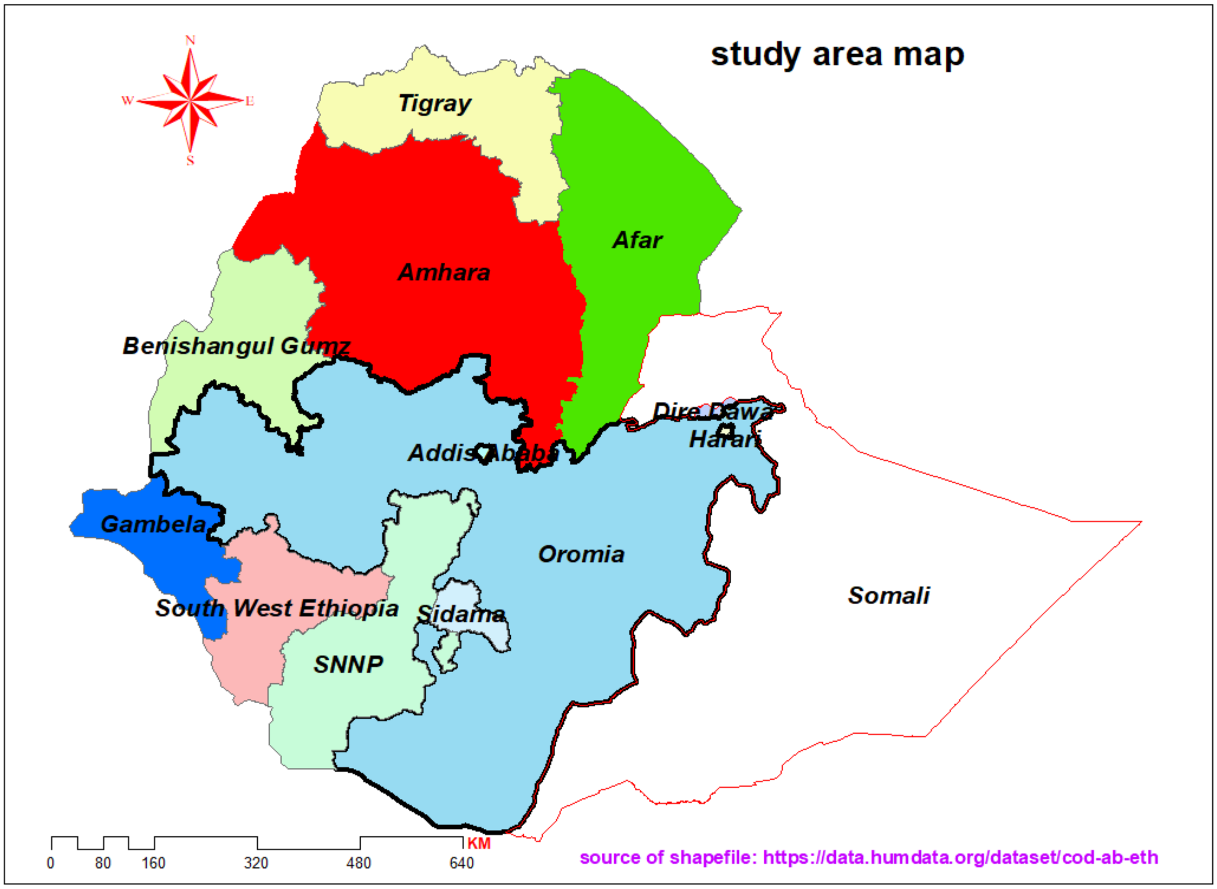

Figure 1: Map of the study area (Ethiopia) in 2021.

Population

Source Population

All health care workers in Ethiopia.

Study Population

Healthcare workers who were directly involved in assessment of sick children exhibiting the three main symptoms: fever, diarrhea and cough/difficulty of breathing were included in this study.

Data sources

Data for this study was obtained from the second E-SPA survey which was collected from 11 August 2021 to 4 February 2022. The survey process was led by Ethiopian public health institute (EPHI) and ministry of health (MOH) and financed by United States Agency for International Development (USAID). The main objective of the 2021 E-SPA was to collect information on the availability and delivery of healthcare services in the country. It also examines the readiness of health facilities to provide quality health services. The main focus areas of E-SPA were child health, maternal and newborn care, family planning, sexually transmitted infections (STI), HIV and AIDS, tuberculosis (TB), malaria, and other diseases such as non-communicable diseases (NCD) and neglected tropical diseases (NTD) (2).

Study variables

Dependent variable

IMCI guideline requires each sick child should be checked for three main symptoms: cough or difficulty of breathing, diarrhea, and fever. Those three main symptoms information might be

gotten from the caretaker as the main complaint or the healthcare professional may probe for information about the symptoms. In this study health professionals who assessed all the three main symptoms are put under the categories of "adherent to IMCI guideline" and those who failed to assess all the three symptoms are categorized as "non-adherent to IMCI guideline".

Independent variables

Following an extensive literature review, the variables selected for inclusion in the multilevel multivariable regression analysis encompass health professional's academic qualification, child's age, sex of care provider, sex of child, types of case (referral/non-referral), and children of place of residency as individual-level factors. And region and facility types are considered as facility level factors. These variables were identified as crucial factors, drawing on existing research and literature, and examined collectively to gain a comprehensive understanding of their potential influence on the adherence to IMCI.

Sample Size and Sampling Procedures

Sampling frame

The survey utilized a master list from the Ministry of Health, comprising 25,711 functioning health facilities in Ethiopia, including 413 hospitals, 3,556 health centers, 16,841 health posts, and 4,942 private health facilities, as our sampling frame.

Sampling procedures

Stratification was realized by first separating the health facilities in each administrative region by facility type. The sample allocation featured a power allocation across regions, to achieve comparable survey precision. All hospitals in Ethiopia, whole higher clinics and entire health

centers in Harari and Dire Dawa were included in the study because of their small number. Using power allocation sample features across all regions, 310 health centers, 356 clinics and 328 health posts were selected to be surveyed.

Fifteen Healthcare providers were interviewed from each health facilities and all healthcare providers were interviewed in facilities having total provider less than fifteen in number. Health facilities having greater than 15 health professionals were sampled randomly (via lottery) method after taking their qualifications, department in which they work, and the services they provide into consideration. Finally, a total of 788 (weighted) children who have experienced any of the three main symptoms and have been seen at any of the selected health facilities are included in this study.

Data analysis

Spatial analyses

To explore, create, visualize and edit the spatial information of IMCI adherence of health workers in Ethiopia, we employed ArcGIS version 10.8 software. For assessing the spatial pattern of health professionals who are non-adherent to IMCI guidelines, we utilized an inferential statistic known as the Spatial Autocorrelation (Global Moran's I) tool (14). This tool calculates the Moran's Index(I) value along with a z-score and p-value to determine whether the pattern of vaccine hesitancy is scattered, clustered, or random. The significance of Moran's Index is evaluated based on both the z-score and p-value.

But, to search for the local hotspot areas (local areas having higher occurrence of non-adherent health professionals to IMCI guideline than expected), purely spatial analysis was employed using SaTScan™ version 10 software. The analysis utilized the Bernoulli model-based purely

spatial scan statistic with 1/0 event data, where cases (1) represent "**non-adherent"** and controls (0) represent "**adherent"** to follow IMCI guideline while assessing the three main symptoms of childhood illness. We retained the default maximum spatial cluster size setting (50% of the population at risk). This setting is recommended as it allows for the identification of clusters of both small and large size without pre-selection bias regarding cluster size.

Multivariable multilevel analysis

To account for the sharing of similar characteristics at health facilities, we developed a two-level multivariable multilevel logistic regression model that health workers nested in their health facility type (hospital, health center and private health facility). Bivariate analyses were performed to assess the association between IMCI adherence and each exposure variable (one variable at a time) using multilevel logistic regression models. Variables that were considered statistically significant or which displayed an association with IMCI adherence in the bivariable models with a P-value of less than 0.25 were included in the final models. Exposure variables were checked for multicollinearity using variance inflation factors (VIF). Variables with VIFs greater than 10 were considered to be indicative of multicollinearity and were removed from the final models(15).

We developed four models to identify the determinants of IMCI adherence. The fully unconditional model (Model 1) was built with no predictors and aimed to describe the components of the total variance in the odds of IMCI adherence and the intra-class correlation (ICC). The next model (Model 2) was adjusted for individual level (level-one) factors that showed bivariable associations with IMCI adherence. The next model (Model 3) was adjusted for the individual-level (level two) factor of mother's country of birth. This was the only maternal level variable included in our study. All other variables could vary between newborns.

The final model (Model 4) was adjusted for both newborn-level and maternal-level factors. This model included all the variables in Model 2 and Model 3. Adjusted odds ratios with 95% confidence intervals (CI) were used as estimates of the association between individual and facility-level factors and the odds of IMCI adherence. All analyses were performed in Stata 16(16).

Measures of random effects the intra-class correlation was used as a measure of whether the variation in IMCI adherence was primarily between-facilities or within-facilities. ICC is the ratio of the level-two variance to the total variance and takes a value between 0 and 1, with higher values suggesting greater importance of facility-level factors(17) in understanding the individual differences in IMCI adherence. The proportional change in variance (PCV) was used as a measure of the change in variance between different models (18). In this case, PCV was used to measure the total variation in IMCI adherence that was attributable to individual-level factors and facility-level factors.

Goodness of fit Goodness of fit was assessed for each of the four models using Akaike's information criterion (AIC) and Bayesian information criteria (BIC) (19)to select the model with the optimal fit. Lower values of AIC and BIC were indicative of better fit.

Ethical consideration

This study made use of datasets obtained from the nationally representative Service Provision Assessment (SPA) survey. The secondary data used in this study are completely anonymized and cannot be traced back to individual participants, and the original data collection process ensured confidentiality and ethical standards. Therefore, additional consent is not be necessary for this study. Ethical approval was not considered necessary, given that the datasets were

requested through a formal process that involved providing a clear explanation of the study's objectives. The researchers registered their study and submitted a request for the SPA-Ethiopia dataset through the online database (https://dhsprogram.com/). Subsequently, they received authorization in the form of a letter that granted permission to download the requested datasets, ensuring adherence to ethical standards and data usage protocols.

Results

A total of 788 sick children who were assessed for at least one the main three symptoms of childhood illness (fever, cough and diarrhea) were included in this study. Majority of the participants were from Oromia region 1528(41%) and followed by Amhara region 740(20%) (Table 1). The median age of children who participated in this study is 18 months (IQR: 9-31 months).

Table 1: demographic characteristics of study participants

Variable	Frequency (weighted sample)	Percent
**Children's age**		
2-24 months	2329	63.96
≥24 months	1312	36.04
**Sex of child**		
Male	2021	55.51
Female	1620	44.49
**Children's place of residency**		
Urban	1718	47.20
Rural	1923	52.80
**Case type**		
Cold case (not referral)	596	16.44
Referral	3032	83.56
**Sex of healthcare provider**		
Male	2590	
Female	1051	71.13
**Qualification of care provider**		28.87
Nurse	2232	61.32

HO	914	25.10
MD	494	13.58
**Facility type**		
Private HF	1246	34.21
HC	2035	55.90
Hospitals	260	9.88
**Region**		
Afar	72	1.98
Amhara	740	20.32
Oromia	1528	41.96
Somali	187	5.15
Benishangul	96	2.65
S.N.N. P	571	15.69
Gambella	75	2.07
Harari	18	0.5
Addis Ababa	237	6.51
Dire Dawa	29	0.78
Sidama	87	2.38

The reported adherence rate to IMCI protocols in Ethiopia is 33%, with a 95% confidence interval ranging from 29.7% to 36.3%. Notably, certain regions exhibit rates significantly above the national average, reflecting varying percentages of adherence toward IMCI guideline. Benishangul Gumuz, Dire-Dawa, Harari, and Gambella stand out with increased rates. Oromia (30%) and Sidama (29%) are among the lowest performing regions (Figure 2).

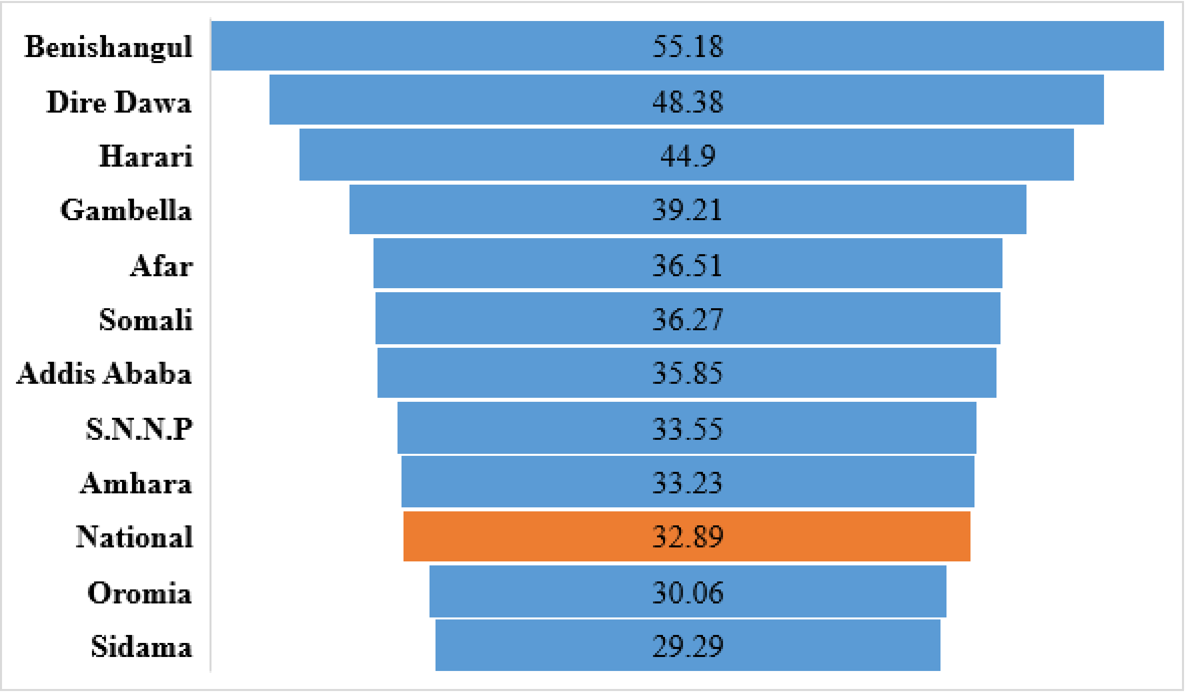

Figure 2: health professional Adherence rate to IMCI guideline in Ethiopia by administrative region, 2021.

Health workers adherence rate toward IMCI guideline is found is higher during assessment of fever i.e., 70%. Higher percentage of adherence is followed during assessing children aged ≤ 24 months (36%), among children assessed by health officers (44%) and children seen at health centers (42%) (Table 2).

Table 2: Adherence rate of health professionals towards IMCI protocols while assessing the three symptoms of sick child in Ethiopia, 2021.

IMCI items	Overall adherence	Age of child		Qualification of the provider			Types of HF		
		≤24 months	>24 months	Nurse	HO	MD	Private HFs	HCs	Hospitals
**Fever**	70.55%	73%	64%	63%	83%	80%	65%	73%	77%
**Diarrhea**	50.38%	49%	52%	49%	51%	53%	45%	54%	49%
**Cough or difficulty of breathing**	60.17%	61%	59%	57%	67%	65%	57%	61%	64%
**All 3 symptoms in one**	33%	36%	28%	27%	44%	36%	22%	42%	33%

Note: HO- health officer, MD-medical doctor, HFs health facilities, HC-health centers

Locations of non-adherent health workers

The SaTScan statistics detected clusters categorized into primary and four secondary clusters. A total of 87 statistically significant location IDs (EAs) were found in those categories of clusters. Out of these, 13 EAs were located inside the window of the most likely cluster (a crucial focus point for targeted interventions) and the remaining clusters were classified as secondary clusters. (Table 3).

SaTScan results indicated that primary clusters (cluster with high LLR, 20.82) mainly converged on west Oromia region (Figure 3), centered at (7.982108 N, 36.203355 E) within a radius of 78.86 km (Table 3). The relative risk 1.55, as depicted in table 3, can be interpreted as the probability of non-adherence in the primary cluster is 55% higher compared to care provider outside the cluster.

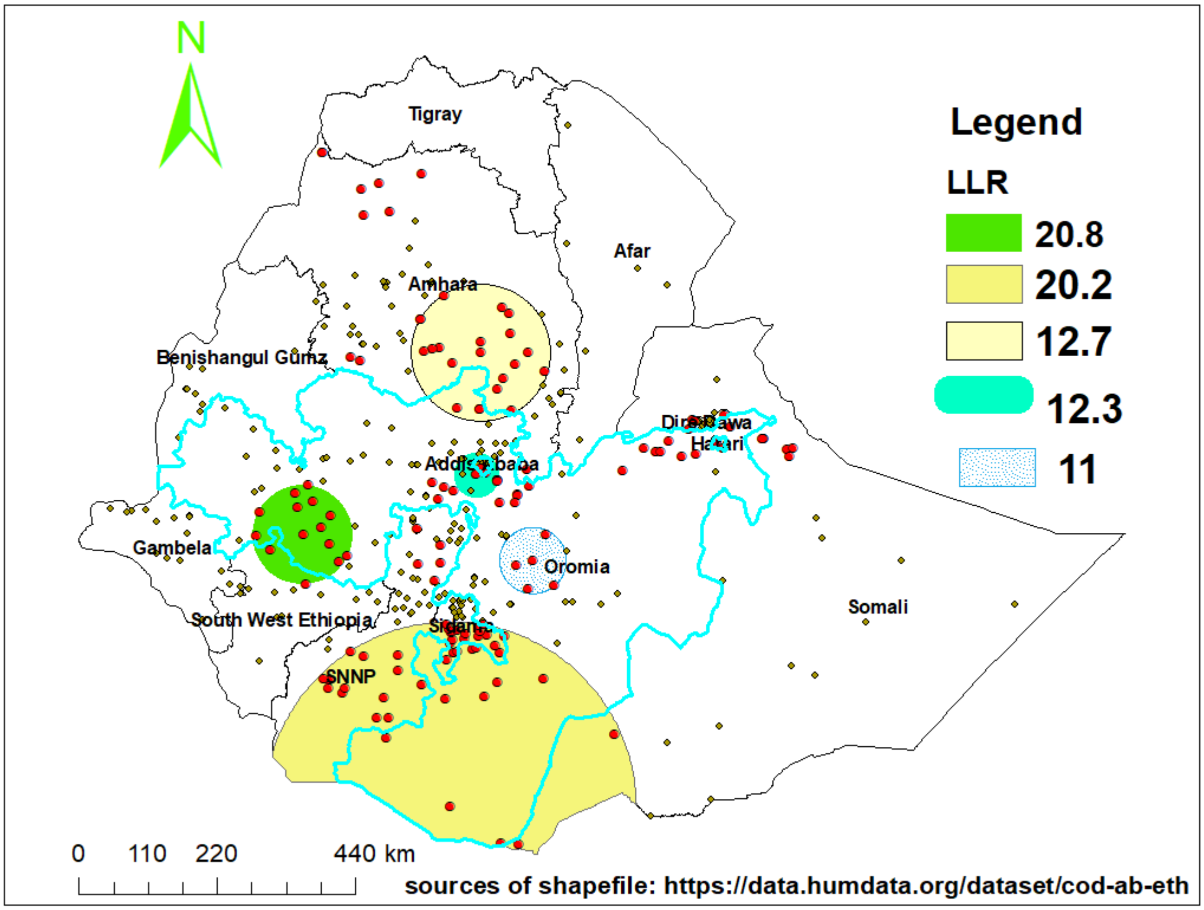

Figure 3: location of statistically significant spatial clustering of health care providers which are not adherent to IMCI in Ethiopia, 2021.

Table 3: all clusters of non-adherent health professionals identified through SaTScan in Ethiopia, 2021.

Types of clusters	Coordinates/Radius	No. cluster	Cluster location	Expected cases	Observed cases	p-value
Most likely cluster	(7.982108 N, 36.203355 E) / 78.86 km	13	West Oromia	34	50	0.00000027

Secondary cluster 1	(4.076593 N, 38.317550 E) / 295.43 km	44	South Oromia, south-east of SNNP regions	57	80	0.00000045
Secondary cluster 2	(10.593845 N, 38.753502 E) / 108.98 km	21	Southern Amhara region	21	31	0.00056
Secondary cluster 3	(8.839652 N, 38.693485 E) / 12.45 km	4	Addis Ababa	20	30	0.00082
Secondary cluster 4	(7.614975 N, 39.500907 E) / 52.73 km	5	Oromia region	18	27	0.0027

Multilevel multivariable logistic regression

Measures of variation

An ICC value of 0.5523 in model 1 indicates that more than half (55.23%) of the variability in IMCI adherence odds is attributed to differences between facilities. When individual-level factors were exclusively considered in the model (Model 2), the corresponding ICC decreased to 52.1%, indicating a reduced contribution of between-individual variation to the explained variance (ICC = 0.521). Model 4, which accounted for both individual-level and facility-level factors, demonstrated a nearly identical proportion of total variance in the odds of IMCI explained by between-facility variation compared to Model 3 (ICC = 0.536). Moreover, the PCV in Model 2 indicated that the addition of the predictors explained an increased proportion of variation in the odds of IMCI adherence (PCV = 4.25).

As illustrated in Table 4, the process of selecting the model with the optimal fit was guided by lower values of AIC (Akaike Information Criterion) and BIC (Bayesian Information Criterion), both of which are indicators of model fit. Accordingly, Model 4, characterized by lower AIC and BIC values, emerged as the best-fitted model for this dataset.

Table 4: Measure of facility level variation in model 1 to 4.

	Model 1	Model 2	Model 3	Model 4
**Measure of variation for IMCI adherence**				
Facility level variance (SE)	4 (0.86)	4.17 (0.9)	3.79 (0.82)	3.84 (0.85)
ICC (%)	55.23	52.11	53.58	53.89
Explained variance PCV (%)	Ref	4.25	5.25	4
**Model fitness**				
AIC	807	795	815	791
BIC	816	838	882	844

Factors associated with adherence

In the realm of multivariable modeling, certain factors emerge as noteworthy contributors to the adherence rate of health workers to the IMCI guideline in Ethiopia. It is worth noting that the age of the child (being over 24 months), the type of health facility (specifically, health centers), the child's place of residency (rural areas), and the academic qualification of the care provider all demonstrate themselves as significant variables correlated to adherence to the IMCI guideline.

Regarding relationship between age of children and IMCI adherence, children in the higher age group (>24 months) show a 34% lower likelihood of adherence [AOR=0.66, 95% CI: 0.45, 0.87] compared to those in the lower age group. Furthermore, children residing in rural communities face a heightened risk of not being assessed according to the IMCI protocols. Health workers are 36% less likely to adhere to the IMCI protocols [AOR=0.54, 95% CI: 0.38, 0.77] when assessing children in rural areas compared to those in urban areas.

Regarding the academic qualification, a discernible difference was shown. The likelihood of adherence is 71% higher [AOR=1.71, 95% CI: 1.18, 2.48] for health officers compared to the base group of nurses.

Analysis of facility-level factors reveals a significantly higher likelihood of adherence to the IMCI guideline. Specifically, healthcare providers in health centers demonstrate a 2.61 times greater adherence to IMCI protocols [95% CI: 1.84, 3.37] compared to their counterparts in private health facilities (Table 5)

Table 5: displays the results of the multivariable logistic analysis of adherence rate to IMCI protocol among health professionals in Ethiopia, 2021.

Table 5: multilevel multivariable logistic regression results model 4.

Variable	COR (95% CI)	AOR (95% CI)	p-value of AOR
**Child's age in months**			
≤24 months(ref)			
>24 months	0.67 (0.49, 0.91)	**0.66 (0.45, 0.87)**	**0.004**
**Health facility type**			
Private HF (ref)			
Health center	2.49 (1.77, 3.48)	**2.61 (1.84, 3.37)**	**0.000**
Hospital	1.72 (1.06, 2.81)	1.35 (0.70, 2.61)	0.405
**Case type**			
None referral (ref)			
Referral	0.85 (0.57, 1.29)	0.91 (0.58, 1.43)	0.78
**Child's Place of residency**			
Urban (ref)			
Rural	0.55 (0.41, 0.75)	**0.54 (0.38, 0.77)**	**0.000**
**Qualification of care provider**			
Nurse (ref)			
Health officer	2.15 (1.51, 3.06)	**1.71 (1.18, 2.48)**	**0.012**
Medical doctor	1.54 (1.03, 2.32)	1.26 (0.67, 2.37)	0.477
**Sex of provider**			
Male (ref)			
Female	0.76 (0.55, 1.04)	0.89 (0.63, 1.27)	0.545
**Sex of child**			
Male (ref)			
Female	0.79 (0.58, 1.06)	0.75 (0.54, 1.03)	0.076
**Region**			
Afar (ref)			
Amhara	0.46 (0.009, 22.67)	0.47 (0.008, 25)	0.712
Oromia	0.70 (0.015, 32.12)	0.89 (0.017, 45.84)	0.957
Somali	1.12 (0.018, 67.00)	1.07 (0.016, 71.7)	0.974

Benishangul	2.84 (0.033, 243.8)	9.31 (0.094, 919.6)	0.341
S.N.N.P	1.04 (0.022, 49.15)	1.14 (0.021, 60.88)	0.946
Gambella	1.63 (0.02, 123.78)	2.19 (0.025, 187.3)	0.729
Harari	1.71 (0.011, 263.3)	1.81 (0.010, 319.9)	0.821
Addis Ababa	0.89 (0.018, 44.34)	0.55 (0.099, 31.13)	0.775
Dire Dawa	2.28 (0.039, 131.9)	2.59 (0.04, 168.33)	0.654
Sidama	0.84 (0.016, 42.96)	1.18 (0.02, 68.41)	0.934

NOTE: ref- reference, COR-Crude odds ratio, AOR- adjusted odds ratio, HF-health facility,

Discussion

We explore findings from a detailed analysis, including spatial SaTScan, multilevel multivariable analysis, and an examination of IMCI adherence percentages using the national survey data ESPA. These approaches offer a thorough insight into geographic patterns, individual and facility-level determinants, and the overall percentage of IMCI adherence in assessing three childhood illnesses.

According to this study, the adherence of health workers to the IMCI guideline in Ethiopia is low (33%), with a 95% confidence interval of [29.7, 36.3]. This prevalence is consistent with research conducted in Tanzania(20) and Kenya(21), which reported a prevalence of 28.4% and 34%, but lower than study done using the SPA data of Nigeria (48%), Tanzania (50%), Uganda (57%) (19%) (21). The variations in IMCI adherence rates may be attributed to differences in socio-demographics, geography, sample sizes, healthcare infrastructure, training programs, and health system disparities between countries. Other contributing factors include the diverse cadres of health workers analyzed in these studies. Additionally, differences may arise from variations in the assessment domains; our study assessed adherence using only the three main symptoms, whereas other studies incorporated danger signs and physical examinations, either separately or in combination with the three main symptoms (22) of childhood illness.

This study identified a significant proportion of healthcare providers showing reluctance to adhere to IMCI protocols, particularly in geographic areas with a higher number of non-compliant health workers, notably in the Amhara, Afar, SNNP, Sidama, and Oromia regions. The concentration of non-adherent health professionals in those areas could stem from insufficient training, understaffing, high workload, lack of awareness, and inadequate supervision and monitoring by higher officials. We urge future researchers to conduct a thorough and detailed assessment to identify the potential root causes, to identify challenges and specific barriers contributing to non-adherence in those identified hotspots geographic areas. We recommend policy makers and program implementers to install evidence-based policy recommendations to address systemic issues contributing to non-adherence.

It appears that in this study, there is an observed inverse relationship between the age of sick children and adherence to IMCI guidelines. Specifically, the finding suggests that as the age of the child increases, the odds of adherence to IMCI decrease. This finding is consistent with study done using four African countries' SPA datasets(21). This might be because healthcare providers spent more time assessing younger children than older ones(22). Another reason might be due to work overload(23, 24) or that healthcare providers are less likely to stick to IMCI recommendations for older children, possibly due to assumptions that older children are more resilient to illness or perceived conditions as less severe. Not sticking to the recommendation of IMCI guideline during assessment of fever, diarrhea or difficulty of breathing in older children is (≥24 months) could result in suboptimal health outcomes, misclassification of diseases, increased cost, further complications, and long stay in hospital. The health governing body in the country should focus on the need for enhanced training and capacity building for healthcare professionals to ensure that

they are equipped with the knowledge and skills required to appropriately manage childhood illnesses in older children.

Another finding of this study is children living in rural settings of Ethiopia face Rural heightened challenges when it comes to adhering to the IMCI guidelines, particularly in comparison to their urban counterparts. Similar finding is also reported by another study done in the united states of America (25) which concluded that rural dwellers are less likely to get preventive health services based on recommended healthcare guidelines. This might be due to that rural-urban divide of skilled healthcare workers. In general, rural healthcare settings face a shortage of skilled health professionals in general(26, 27) and more skilled health professionals are concentrated in urban areas than rural settings in many sub-Saharan countries(28). Another justification for this finding might be the lower levels of education in rural areas caregivers (12, 29) may contribute to a lack of awareness and parents' ability to recognize and respond to signs of illness in their children. Addressing education, community awareness, closing the gap of skilled health professional distribution in rural areas related challenges could contribute to narrowing the gap in adherence to IMCI guidelines between rural and urban populations, ultimately improving the health outcomes of children in rural areas.

From care provider related factors, being a professional qualified in health officer (HO) is found to have an increased likelihood of following the recommendation of IMCI protocols during assessing children who come with complaints of fever, difficulty of breathing or diarrhea compared to nurse professionals. A possible explanation for this might be Health officers typically undergo specific education on IMCI protocols during their professional development at university than nurse professionals. According to the Ethiopian curriculum, health officers have a wider scope

of practices related to clinical skills, including the comprehensive assessment and management of childhood illnesses(30).

Finally, the facility level factors revealed a significant difference in the likelihood of children receiving clinical services in accordance with IMCI recommendations based on the type of healthcare facility visited. Specifically, children who had sought care in health centers demonstrated a notably higher likelihood, nearly 2.61 times more, of receiving clinical services aligned with IMCI guidelines compared to their counterparts who were attended to in private health facilities. This finding is consistent with other studies done in other African countries such in Uganda(31), north-west Ethiopia(32), Nigeria(33, 34), western Kenya (35) which reported that the provision of health services in accordance with their national guidelines is unsatisfactory. It seems possible that these results are due to the major gap of the country in regulating the health workers in private health facilities (36). Other alternative explanations for this result are that private health facilities may face resource constraints, limiting their ability to invest in staff training, and other resources required for implementation of IMCI recommendations. This finding suggests that targeted intervention specifically to private health facilities is required to ensure that children, regardless of the type of healthcare facility, receive clinical services in accordance with IMCI recommendations.

Strengths and limitation of the study

Finally, several strengths and limitations can be pinpointed from this study. The main strength of this study is the utilization of most recent national data to study adherence of health workers to IMCI guideline. The authors of this explored the clustering of health professionals reluctant to adhere to IMCI guideline using the emerging statistical analysis called spatial analysis in the

context of health systems. Location specific response to health problems based on identified clusters could help in narrowing health inequalities.

The national survey data utilized in this study omits data of Tigray region. So, the exclusion of Tigray region's data from this study might have limited the generalizability of findings to the entire population of the country. The opportunity to gain insight into the Tigray region about prevalence, contributing factors and hotspot areas of adherence to IMCI protocols are missed and this could impair region-specific policy recommendations. In relation to the cross-sectional aspect, SPA Ethiopia encounters analogous constraints to other cross-sectional surveys i.e., cannot be used to determine the cause of something. Hence, we used data from secondary survey, missing other behavioral and cultural factors in this study could compromise model performance.

Conclusion

The rate of adherence to IMCI guideline in Ethiopia is low. Low adherence could have various implications, including potential gaps in the quality of child healthcare and the prevention and management of childhood illnesses. By tailoring interventions to the unique needs of each geographic area, this targeted plan aims to create a more equitable distribution of health professionals adhering to IMCI protocols, ultimately improving child healthcare outcomes across the region. A full implementation of IMCI programs should consider several key factors to ensure comprehensive and effective coverage like the diverse needs and contexts of children, healthcare facilities, and providers.

Additional research is necessary to conduct thorough analyses in hotspot areas and to examine changes in IMCI adherence over time, exploring spatio-temporal relationships. It is advisable to undertake studies comparing hotspot areas with non-hotspot regions to discern any noteworthy

differences or similarities influencing variations in adherence. Furthermore, we suggest that future researchers employ qualitative research methods to gather local perspectives on IMCI adherence in hotspot areas, providing nuanced insights into community beliefs and attitudes.

Funding

The authors received no specific funding for this work.

Contributions

AAT, AK, NK, YT, AE, SDK, KMA, ET, EBE, CD, LA, FDB, MA and AM were involved in the design and conception of the study; the analysis, and interpretation of the findings. All the authors read and approved the manuscript starting from the first step up to this.

Abbreviations

AIC: Akaike information criteria, **AOR**: Adjusted Odds Ratio, **BIC**: Bayesian information criteria**, ESPA**: Ethiopian Service Provision Assessment, **DIC**: Deviance Information Criterion, **ICC**: Intra-Class Correlation, **LLR**: Log-Likelihood Ratio, **PCV**: Proportion of Variance Change, **CI**: confidence interval, **COR**: Crude Odds Ratio, **AOR**: Adjusted odds ratio, **EA**: enumeration areas, **SNNP**: South Nation and Nationalities People, **KM**: Kilometer, **IMCI**: Integrated Management Childhood Illness, **EPHI**: Ethiopian public health institute, MOH: Ministry of Health, **USAID**: United States Agency for International Development, TB: tuberculosis, **NCD:** Non-Communicable Diseases**, NTD:** Neglected Tropical Diseases, **VIF**: variance inflation factors, **HO**: health officer, **MD**: Medical Doctor, **HF**: health facility, **HC**: health center**, ID/EA**: cluster ID/ enumeration areas, **IQR**: interquartile range.

Ethics approval and consent to participate

As the authors utilized secondary data from 2021-SPA Ethiopia, consent of participants and ethical approval is not required. But the authors have been granted access to the dataset of 2021 SPA-Ethiopia through registering to https://www.dhsprogram.com/ websites.

Consent for publication

Not applicable

Availability of data and material

The datasets and materials are available at https://www.dhsprogram.com. The minimal data used for this study are available from the corresponding author on reasonable request.

Competing interests

The authors declares that he has no competing interests

Reference

1. Ministry of Health - MoH/Malawi, ICF International. Malawi Service Provision Assessment 2013-14. Lilongwe, Malawi: MoH/Malawi and ICF International; 2014.
2. Ethiopian Public Health Institute, Ethiopia Ministry of Health, ICF. Ethiopia Service Provision Assessment 2021–22. Addis Ababa, Ethiopia, and Rockville, Maryland, USA: EPHI, MoH and ICF; 2023.
3. Bryce J, Victora CG, Habicht J-P, Black RE, Scherpbier RWJHp, planning. Programmatic pathways to child survival: results of a multi-country evaluation of Integrated Management of Childhood Illness. 2005;20(suppl_1):i5-i17.
4. Black RE, Morris SS, Bryce JJTl. Where and why are 10 million children dying every year? 2003;361(9376):2226-34.
5. Lulseged SJEmj. Integrated management of childhood illness: a review of the Ethiopian experience and prospects for child health. 2002;40(2):187-201.
6. Gove SJBotWHO. Integrated management of childhood illness by outpatient health workers: technical basis and overview. The WHO Working Group on Guidelines for Integrated Management of the Sick Child. 1997;75(Suppl 1):7.

7. Nguyen DTK, Leung KK, McIntyre L, Ghali WA, Sauve RJPo. Does integrated management of childhood illness (IMCI) training improve the skills of health workers? A systematic review and meta-analysis. 2013;8(6):e66030.
8. Abayneh M, Gebremedhin T, Dellie E, Tsehay CT, Atnafu AJIJoP. Improving the Assessment and Classification of Sick Children According to the Integrated Management of Childhood Illness (IMCI) Protocol at Sanja Primary Hospital, Northwest Ethiopia: A Pre-Post Interventional Study. 2020;2020.
9. Assefa Y, Gelaw YA, Hill PS, Taye BW, Van Damme WJG, health. Community health extension program of Ethiopia, 2003–2018: successes and challenges toward universal coverage for primary healthcare services. 2019;15:1-11.
10. Robinson DJAh. The integrated management of childhood illness. 1996;18(6):20-1.
11. Federal Ministry of Health Ethiopia %J FMOH AA, Ethiopia. Health Sector transformation plan, 2015/16-2019/20. 2015.
12. Central Statistical Agency - CSA/Ethiopia, ICF. Ethiopia Demographic and Health Survey 2016. Addis Ababa, Ethiopia: CSA and ICF; 2017.
13. Snow JJAA. Health facility End-line Survey-synthesis report. 2008.
14. Sheng J, Han X, Zhou H. Spatially varying patterns of afforestation/reforestation and socio-economic factors in China: a geographically weighted regression approach. Journal of Cleaner Production. 2017;153:362-71.
15. Midi H, Sarkar SK, Rana S. Collinearity diagnostics of binary logistic regression model. Journal of Interdisciplinary Mathematics. 2010;13(3):253-67.
16. StataCorp L. Stata statistical software: release 14. Statacorp lp College Station, TX; 2015.
17. Merlo J, Chaix B, Yang M, Lynch J, Råstam LJJoE, Health C. A brief conceptual tutorial of multilevel analysis in social epidemiology: linking the statistical concept of clustering to the idea of contextual phenomenon. 2005;59(6):443-9.
18. Merlo J, Chaix B, Ohlsson H, Beckman A, Johnell K, Hjerpe P, et al. A brief conceptual tutorial of multilevel analysis in social epidemiology: using measures of clustering in multilevel logistic regression to investigate contextual phenomena. 2006;60(4):290-7.
19. Vrieze SIJPm. Model selection and psychological theory: a discussion of the differences between the Akaike information criterion (AIC) and the Bayesian information criterion (BIC). 2012;17(2):228.
20. Lange S, Mwisongo A, Mæstad OJSs, medicine. Why don't clinicians adhere more consistently to guidelines for the Integrated Management of Childhood Illness (IMCI)? 2014;104:56-63.
21. Krüger C, Heinzel-Gutenbrunner M, Ali MJBHSR. Adherence to the integrated management of childhood illness guidelines in Namibia, Kenya, Tanzania and Uganda: evidence from the national service provision assessment surveys. 2017;17(1):1-12.
22. Adam T, Manzi F, Schellenberg JA, Mgalula L, de Savigny D, Evans DBJBotWHO. Does the Integrated Management of Childhood Illness cost more than routine care? Results from the United Republic of Tanzania. 2005;83(5):369-77.
23. Adam T, Amorim DG, Edwards SJ, Amaral J, Evans DBJHp, planning. Capacity constraints to the adoption of new interventions: consultation time and the Integrated Management of Childhood Illness in Brazil. 2005;20(suppl_1):i49-i57.
24. Edward A, Dam K, Chege J, Ghee AE, Zare H, Chhorvann CJIJfQiHC. Measuring pediatric quality of care in rural clinics—a multi-country assessment—Cambodia, Guatemala, Zambia and Kenya. 2016;28(5):586-93.

25. Casey MM, Call KT, Klingner JMJAjopm. Are rural residents less likely to obtain recommended preventive healthcare services? 2001;21(3):182-8.

26. Watch BJTSoHiB. Health Workforce in Bangladesh: Who Constitutes the Healthcare System. 2007.

27. Ahmed SM, Hossain MA, RajaChowdhury AM, Bhuiya AUJHrfh. The health workforce crisis in Bangladesh: shortage, inappropriate skill-mix and inequitable distribution. 2011;9(1):1-7.

28. Anyangwe SC, Mtonga CJIjoer, health p. Inequities in the global health workforce: the greatest impediment to health in sub-Saharan Africa. 2007;4(2):93-100.

29. Zhang YJCer. Urban-rural literacy gaps in Sub-Saharan Africa: The roles of socioeconomic status and school quality. 2006;50(4):581-602.

30. Negash FJSIJ. Health Officer: A Physician Assistant Comparable Role in Ethiopia. 2021;8.

31. Mpimbaza A, Babikako H, Rutazanna D, Karamagi C, Ndeezi G, Katahoire A, et al. Adherence to malaria management guidelines by health care workers in the Busoga sub-region, eastern Uganda. 2022;21(1):1-15.

32. Argaw MD, Mavundla TR, Gidebo KDJBHSR. Management of uncomplicated malaria in private health facilities in North-West Ethiopia: a clinical audit of current practices. 2019;19(1):1-14.

33. Mokuolu OA, Ntadom GN, Ajumobi OO, Alero RA, Wammanda RD, Adedoyin OT, et al. Status of the use and compliance with malaria rapid diagnostic tests in formal private health facilities in Nigeria. 2016;15(1):1-11.

34. Udoh E, Oyo-Ita A, Odey F, Effa E, Esu E, Oduwole O, et al. Management of uncomplicated malaria in underfives in private and public health facilities in South-eastern Nigeria: a clinical audit of current practices. 2013;2013.

35. Riley C, Dellicour S, Ouma P, Kioko U, ter Kuile FO, Omar A, et al. Knowledge and adherence to the national guidelines for malaria case management in pregnancy among healthcare providers and drug outlet dispensers in rural, western Kenya. 2016;11(1):e0145616.

36. Dejene D, Yigzaw T, Mengistu S, Ayalew F, Kahsaye M, Woldemariam DJGHR, et al. Exploring health workforce regulation practices and gaps in Ethiopia: a national cross-sectional study. 2019;4(1):1-12.
