# Supplementary material for: Adherence to IMCI guidelines for key symptoms in Ethiopian children: A 2021–2022 national service provision survey
Source: PLoS One. 2024 Oct 16;19(10):e0312138. doi: 10.1371/journal.pone.0312138 (PMC11482708; doi:10.1371/journal.pone.0312138)
Supplement: Attachment — Submitted filename: PONE-D-24-03866_R1.pdf [file pone.0312138.s003.pdf]

PLOS ONE

Assessing Health Professionals' Adherence to IMCI Protocols for Key Symptoms in Sick Children in Ethiopia: Findings from the 2021-2022 National Service Provision Survey.

--Manuscript Draft--

**Manuscript Number:**	PONE-D-24-03866R1
**Article Type:**	Research Article
**Full Title:**	Assessing Health Professionals' Adherence to IMCI Protocols for Key Symptoms in Sick Children in Ethiopia: Findings from the 2021-2022 National Service Provision Survey.
**Short Title:**	Adherence to IMCI protocols
**Corresponding Author:**	Abiyu Abadi Tareke<br>Amref Health Africa<br>GONDAR, ETHIOPIA
**Keywords:**	adherence, factor associated, IMCI protocol, Spatial analysis, Ethiopia
**Abstract:**	Background: The Health Services Provision Assessment in Ethiopia (SPA-ET) is a type of survey that generate data related to the availability and quality of health services in Ethiopia. Despite the presence of integrated management of childhood illness protocols in healthcare settings, there has been scare exploration or assessment of how effectively and consistently health professionals are following these guidelines in the context of child healthcare.<br>Objective: This study aims to identify factors influencing healthcare worker adherence to integrated management of childhood illness protocols to pinpoint areas with high non-adherence rates.<br>Methods: The data used in this study was collected through the Service Provision Assessment in Ethiopia survey; a nationwide survey conducted from 11 August 2021 to 4 February 2022. It included a total of 788 health professionals who assessed sick children experiencing at least one of the three main childhood illness symptoms: fever, cough, or diarrhea. We employed STATA version 16 for data analysis, utilizing cross-tabulations to explore relationships between variables and logistic regression modeling to identify factors influencing adherence. To account for the hierarchical structure of the health survey data, we employed multilevel logistic regression. Model selection was based on comparison parameters including the Bayesian Information Criterion (BIC) and Akaike Information Criterion (AIC). We computed adjusted odds ratios with 95% confidence intervals, and statistical significance was determined at a significance level of $p < 0.05$.<br>Results: The rate of adherence to the integrated management of childhood illness guideline was 33% (95% CI: 29.70%, 36.26%). The analysis revealed several factors influencing adherence to IMCI protocols. child’s age [aOR = 0.66, 95% CI: (0.45, 0.87)], facility type [aOR = 2.61, 95% CI: (1.84, 3.37)], place of residency [aOR = 0.54, 95% CI: (0.38, 0.77)], and care provider’s qualification [aOR = 1.71, 95% CI: (1.18, 2.48)] were all statistically significant. Moreover, the primary cluster is situated in the west Oromia region, with a central focus at coordinates (7.982108 N, 36.203355 E) and extending to a radius of 78.28 km.<br>Conclusion: This study confirms a low adherence rate (33%) among health professionals in Ethiopia to the IMCI guideline for assessing the three main symptoms of sick children. The study identified child’s age, facility type, academic qualification and place of residency as crucial factors correlated with adherence rate. Furthermore, 5 secondary clusters (hotspot area) were identified using SaTScan software. To address the higher protocol refusal, interventional plan needs to be based on academic qualification of care provider, facility type, Age of child and place of residency. Moreover, interventions to reduce non-adherence of IMCI guideline should be location-tailored based on identified hotspot areas in order to restore Guideline related adherence equality.
**Order of Authors:**	Abiyu Abadi Tareke
	Awoke Keleb

Powered by Editorial Manager® and ProduXion Manager® from Aries Systems Corporation

Kaleab Mesfin Abera
Natnael Kebede
Endalkachew Mesfin
Aznamariam Ayres
Yawkal Tsega
Abel Endawkie
Shimels Derso Kebede
Eyob Tilahun Abeje
Ermias Bekele Enyew
Chala Daba
Lakew Asmare
Fekade Demeke Bayou
Mastewal Arefaynie
Asnakew Molla Mekonen

Response to Reviewers:

Authors’ response to reviews
Title: Assessing Health Professionals' Adherence to IMCI Protocols for Key Symptoms in Sick Children in Ethiopia: Findings from the 2021-2022 National Service Provision Survey:
Corresponding author: Abiyu Abadi Tareke (abiyu20010@gmail.com)
Version: 2
Date: May 14, 2024
Point by point response for editors/reviewers’ comments
Manuscript number: PONE-D-24-03866
Dear editor/reviewer:
Dear all,
We express our profound appreciation for the insightful and productive feedback that you have provided. Your invaluable comments have significantly enriched the quality of the manuscript, and have greatly augmented our expertise in the realm of scientific paper writing. The authors have diligently considered each of the comments and queries raised by the editors and reviewers, and have responded to them in a targeted manner. Our comprehensive point-by-point rejoinders to all the comments and questions can be found in the subsequent pages. In addition, an accompanying supplementary document has been enclosed, which showcases the modifications made in detail, using the track changes feature. We also made some change to fix grammatical error in some paragraphs.
Review Comments to the Author
Reviewer #1: Kindly provide details of the outcome variable how was this assessed and how the decision was made regarding the non-adherent workers. I've attached a marked PDF with comments for your reference.
Authors’ Response: Thank you for your feedback. Thank you for your valuable feedback and the attached marked PDF with comments. We appreciate your request for clarification on the outcome variable and assessment methods.
Outcome Variable: Adherence to IMCI Protocols
We defined adherence to IMCI protocols as the complete assessment of three key symptoms in sick children: fever, cough/difficulty breathing, and diarrhea. This aligns with the core components of the IMCI approach for identifying and managing common childhood illnesses.
Adherence was assessed through direct observation of sick child consultations by trained survey interviewers. The interviewers used standardized observation checklists specifically designed to capture adherence to IMCI guidelines for these three key symptoms. But this observational doesn’t include physical examination checklists.
Checklists included items about history of three main symptoms for any sick child:
Did the child have difficulty of breathing or cough?
Did the child have diarrhea?
Did the child have fever?

Powered by Editorial Manager® and ProduXion Manager® from Aries Systems Corporation

Non-Adherence:
A health professional was classified as non-adherent if they did not perform a specific procedure recommended by the IMCI guidelines for assessing one or more of the three key symptoms. The specific criteria for non-adherence were outlined in the observation checklist. But interviewer did not assess whether the information shared was correct or whether findings were interpreted appropriately.

Reviewer #2: Dear author(s), thank you for the opportunity to review your article titled “Adherence of Health Professionals to National IMCI Protocols: A Study on Assessing Three Key Symptoms in Sick Children in Ethiopia - Insights from the 2021 National Service Provision Survey”. Overall, I find your article very interesting and relevant for the journal. However, there are certain points which you can work on to further improve your manuscript:

Authors’ Response: Thank you. Absolutely! We are happy to hear your feedback and suggestions for improvement.

1. Grammar/language: during review, I noticed many typos and certain sentences that would need to be rephrased. In line 142, it is “Health care workers” while in line 144, it is “Healthcare workers”.

Authors’ response: Thank you for bringing these issues to my attention. We apologize for the typos and inconsistencies in the sentences. We carefully reviewed the document again and made the necessary corrections. We appreciate your feedback and attention to detail.

Line 161, “caretaker” replace it with "caregiver".

Authors’ response: Thank you for pointing out the error. We changed in line 161 from "caretaker" to "caregiver" to ensure accuracy. Your attention to detail is greatly appreciated.

Line 247, “Therefore, additional consent is not be necessary for this study”. Line 270, “…toward IMCI guideline is found is higher during…”. Line 367, “… difficulty of breathing in older children is (≥24 months) could result…”. Line 378, “In general, rural healthcare settings face a shortage of skilled health professionals in general(26, 27)…” There is repetition of ‘in general’. All of these sentences need to be rephrased. Please pay attention to upper and lower case letters to use them appropriately, particularly in the titles of the tables and figures. Additionally, a thorough review from a language expert is recommended for further corrections.

Authors’ Response: Thank you for bringing these issues to our attention. We apologize for the errors and repetition in those sentences. Here are the revised versions:

Line 247: "Therefore, additional consent is not required for this study."

Line 270: " The adherence rate of health workers toward the IMCI guideline is higher during the assessment of fever, specifically at 70%. Adherence is higher, specifically 36%, when assessing children aged ≤ 24 months compared to those aged ≥ 24 months. Furthermore, a high percentage of adherence, specifically 44%, was observed when children were assessed by health officers. Similarly, children seen at health centers showed an adherence rate of 42%. "

Line 367: " Not sticking to the recommendations of the IMCI guideline during assessment of three main sickness in older children (≥24 months) could lead to suboptimal health outcomes like misclassification of diseases, increased costs, additional complications, and prolonged hospital stays."

Line 378: "A key challenge for rural healthcare settings in many sub-Saharan countries, as documented evidence shows, is the shortage of skilled health professionals. These professionals are concentrated in urban areas compared to rural settings."

We also ensure proper capitalization, especially in the titles of tables and figures. We appreciate your feedback and recommend a thorough review by a language expert for further corrections. We invite university of Gondar English teachers to edit and comment on our manuscript.

2. Abstract: “BIC and AIC”? always write a full form of non-standard abbreviations. “…survey conducted in 2021.”, write down the duration of the survey as you did later in the Methods and Materials section.

Authors’ Response: Thank you for your feedback. We apologize for the oversight. The survey was conducted over a period of seven months in 2021. We will make sure to include the duration of the survey in the Abstract, as we have done in the Methods and Materials section. We also apologize for not providing the full form of the abbreviations "BIC" and "AIC" in the abstract. In the revised version, we ensured including the full form of these non-standard abbreviations, stating "Bayesian Information Criterion

Powered by Editorial Manager® and ProduXion Manager® from Aries Systems Corporation

	(BIC)" and "Akaike Information Criterion (AIC)" in the abstract.<br>3. Introduction: In abbreviation to E-SPA, you've written “Ethiopian health service provision”. Assessment word is missed. Para 6, “This study is the first study…”, rephrase this sentence.<br>Authors’ Response: In abbreviation to E-SPA (Ethiopian Health Service Provision Assessment), the word "Assessment" was inadvertently missed. We apologize for the error. In the revised version, we will make sure to include the complete abbreviation as "Ethiopian Health Service Provision Assessment (E-SPA)". Thank you for bringing this to our attention, and we appreciate your attention to detail.<br>Para 6, “This study is the first study…”, rephrase this sentence.<br>Authors’ Response: Thank you for your comment. The sentence is re-phased as “This study is the first to assess healthcare worker adherence to IMCI guidelines for evaluating three key childhood illness symptoms.”<br>4. Methods: Line 125, provide a reference for the health service-delivery structure for the readers to understand the context of it. Define your health facility types here which is used a lot in your research. Considering the secondary nature of your data, mention whether ethics and consent were taken into consideration in the original survey. Follow strobe or other relevant guidelines to arrange your article.<br>Authors’ Response: We have now included a reference in the introduction to provide readers with a comprehensive understanding of the health service-delivery structure. This addition will help contextualize our study within the broader healthcare framework. Given the secondary nature of our data, we have addressed the ethical considerations by explicitly mentioning that the original survey, from which our data were derived, adhered to ethical standards and obtained appropriate consent from participants. We have clarified this point to ensure transparency regarding the ethical procedures followed during the original survey. We included your comments under method section >> Ethical consideration section.<br>5. Results: Start with the whole survey demographics then the population of interest or only the later one I.e. for 788 patients. Here you have just included the overall participants' demographics of the original survey. Follow one format for writing confidence intervals.<br>Authors’ Response: To enhance clarity, we will revise the presentation of demographics data to start with the overall survey demographics before focusing on the population of interest, i.e., the 788 patients included in our study. By following this format, readers will have a clearer understanding of the broader demographic characteristics of the survey participants before delving into the specifics of our study population. Dear reviewers thank you for your suggestions. In the initial submission of this manuscript, Table 1 was inadvertently included before removing missing values from the dependent variable, resulting in an inflated sample size of 3641 instead of the correct 788. To rectify this error, we have replaced the values in columns 2 and 3 by copying values from the accurate table generated after removing missing values using the Stata command. We apologize for this oversight and appreciate the valuable feedback from reviewer 1 and reviewer 2, which helped us identify and correct this error.<br>Here you write it as “…with a 95% confidence interval ranging from 29.7% to 36.3%” while in another place you mention it as “(95% CI: 29.70%, 36.26%)”.<br>Authors’ Response: Thank you for pointing out the inconsistency. To ensure uniformity in reporting confidence intervals throughout the manuscript, we will adopt one format for presenting them. Considering both options provided, we suggest using the format "[95% CI: 29.7%, 36.3%]" for consistency. This format is concise and widely recognized, making it easier for readers to interpret the confidence intervals across different sections of the manuscript. We appreciate your attention to detail and will make the necessary revisions accordingly.<br>Line 271, age is written as “≤ 24 months” while in tables it is 2-24 months. This changes the meaning of it. Only follow 2-24 months.<br>Authors’ Response: Thank you for your feedback. Here's the revised paragraph and adjustment to the age representation: We standardized the age representation to "2-24 months" vs “≥24 months” throughout the manuscript to maintain consistency and clarity.<br>Line 270-273, rewrite this whole para in correct order. Additionally, here, when a no. is greater among all it's called “highest” not higher.<br>Authors’ Response: dear reviewer for your surprise this paragraph is also recommended to rephrase by the other peer reviewer. Thank you for your feedback and the additional clarification regarding the rephrasing recommendation by the other

	peer reviewer. Here's the revised paragraph with the correct ordering and terminology: “The adherence rate of health workers toward the IMCI guideline is highest during the assessment of fever, specifically at 70%. Adherence is higher, specifically 36%, when assessing children aged 2-24 months compared to those aged ≥ 24 months. Furthermore, a highest percentage of adherence, specifically 44%, was observed when children were assessed by health officers. Similarly, children seen at health centers showed an adherence rate of 42%.”<br>We appreciate the opportunity to address these concerns and ensure the clarity and accuracy of our manuscript. If there are any further adjustments needed, please let us know.<br>Line 305, “… AIC (Akaike Information Criterion)…” is written as Akaike Information Criterion (AIC). Line 313, “…child being over 24 months”. In other places you mention it as “age >24 months”, maintain consistency in your manuscript.<br>Authors’ Response: Line 305: We adjusted the text to maintain consistency, writing "AIC (Akaike Information Criterion)" as "Akaike Information Criterion (AIC)" throughout the manuscript. Additionally, line 313: We modified the expression "child being over 24 months" to "age ≥24 months" to align with the terminology used elsewhere in the manuscript.<br>6. Discussion: Since general danger signs were not considered in the study, mention them in your limitations. Para 3 and 4 lack sufficient comparisons to past studies. Provide a critical analysis of your findings with the prior studies for the mentioned paragraphs.<br>Authors’ Response: We agree that paragraphs 3 and 4 could benefit from a more in-depth comparison with past studies. We made the necessary revisions.<br>Line 359, “It appears that in this study…” rephrase it simply to “In this study…”. First 2 sentences of the following para have the same meanings, avoid repetition and keep it concise. Line 405, isn’t it counterintuitive that private sectors have resource constraints? Justify your explanation in the text.<br>Authors’ Response: Thank you for your feedback. Here's how we addressed your suggestions: Line 359: We simplified the expression to "In this study..." for conciseness and clarity. Following paragraph: We consolidated the first two sentences to avoid repetition and keep it concise while conveying the same meaning effectively.<br>7. Tables and Figures: Tables 1 and 4 are missing footnotes. Mention in it all the abbreviations included in the respective tables. An ideal table should stand for itself without referring to text. Table 2, avoid phrases like “all 3 symptoms in one”, write it instead as “all 3 symptoms included”. Again age here is ≤24 months. Clear this controversy. Use single terms for a variable across the tables. E.g. here you name it “Facility type” while in Table 5 it is “Health facility type”. In Table 3, the p-values are written inappropriately. E.g one is “0.00000027” other is “0.0027”. Round them off to the same number of digits. Table no.5, avoid writing the p-value as “0.000” instead write it as p <0.001. Adjusted odds ratios are usually written as aOR not as “AOR”.<br>Authors’ Response: Thank you for the detailed feedback on the tables. Here's how we'll address each point: According to your recommendations, In Tables 1 and 4, we included footnotes mentioning all the abbreviations used in the respective tables to enhance clarity and ensure that the tables standalone without referring to the text. Table 2: We modified the phrase "all 3 symptoms in one" to "all 3 symptoms included" accordingly. Regarding the age controversy, we standardized the representation to "<24 months" across all tables and narrations for consistency. Table 3: We round off the p-values to the same number of digits (to four decimal places) for consistency purpose. Table 5: We will adjust the presentation of p-values as "p <0.001" instead of "0.000". throughout our manuscript toughly ensured consistency in variables naming. We decided to continue with “facility type” instead of “types of health facility or health facility type”. Finally, Adjusted odds ratios will be presented as "aOR" instead of "AOR" in Table 5 and other part of the manuscript to maintain consistency with standard conventions.
**Additional Information:**	
**Question**	**Response**
**Financial Disclosure**<br><br>Enter a financial disclosure statement that describes the sources of funding for the	The author(s) received no specific funding for this work.

Powered by Editorial Manager® and ProduXion Manager® from Aries Systems Corporation

work included in this submission. Review the submission guidelines for detailed requirements. View published research articles from *PLOS ONE* for specific examples.<br><br>This statement is required for submission and **will appear in the published article** if the submission is accepted. Please make sure it is accurate.<br><br>**Funded studies**<br>Enter a statement with the following details:<br>• Initials of the authors who received each award<br>• Grant numbers awarded to each author<br>• The full name of each funder<br>• URL of each funder website<br>• Did the sponsors or funders play any role in the study design, data collection and analysis, decision to publish, or preparation of the manuscript?<br><br>Did you receive funding for this work?	
**Competing Interests**<br><br>Use the instructions below to enter a competing interest statement for this submission. On behalf of all authors, disclose any competing interests that could be perceived to bias this work—acknowledging all financial support and any other relevant financial or non-financial competing interests.<br><br>This statement is required for submission and **will appear in the published article** if the submission is accepted. Please make sure it is accurate and that any funding sources listed in your Funding Information later in the submission form are also declared in your Financial Disclosure statement.<br><br>View published research articles from *PLOS ONE* for specific examples.	The authors have declared that no competing interests exist

**NO authors have competing interests**<br><br>Enter: *The authors have declared that no competing interests exist.*<br><br>**Authors with competing interests**<br><br>Enter competing interest details beginning with this statement:<br><br>*I have read the journal's policy and the authors of this manuscript have the following competing interests: [insert competing interests here]*<br><br>* typeset	
**Ethics Statement**<br><br>Enter an ethics statement for this submission. This statement is required if the study involved:<br><br>• Human participants<br>• Human specimens or tissue<br>• Vertebrate animals or cephalopods<br>• Vertebrate embryos or tissues<br>• Field research<br><br>Write "N/A" if the submission does not require an ethics statement.<br><br>General guidance is provided below. Consult the submission guidelines for detailed instructions. **Make sure that all information entered here is included in the Methods section of the manuscript.**	N/A

**Format for specific study types**<br><br>**Human Subject Research (involving human participants and/or tissue)**<br>• Give the name of the institutional review board or ethics committee that approved the study<br>• Include the approval number and/or a statement indicating approval of this research<br>• Indicate the form of consent obtained (written/oral) or the reason that consent was not obtained (e.g. the data were analyzed anonymously)<br><br>**Animal Research (involving vertebrate animals, embryos or tissues)**<br>• Provide the name of the Institutional Animal Care and Use Committee (IACUC) or other relevant ethics board that reviewed the study protocol, and indicate whether they approved this research or granted a formal waiver of ethical approval<br>• Include an approval number if one was obtained<br>• If the study involved *non-human primates*, add *additional details* about animal welfare and steps taken to ameliorate suffering<br>• If anesthesia, euthanasia, or any kind of animal sacrifice is part of the study, include briefly which substances and/or methods were applied<br><br>**Field Research**<br><br>Include the following details if this study involves the collection of plant, animal, or other materials from a natural setting:<br>• Field permit number<br>• Name of the institution or relevant body that granted permission	
**Data Availability**<br><br>Authors are required to make all data underlying the findings described fully available, without restriction, and from the time of publication. PLOS allows rare exceptions to address legal and ethical concerns. See the PLOS Data Policy and FAQ for detailed information.	Yes - all data are fully available without restriction

Powered by Editorial Manager® and ProduXion Manager® from Aries Systems Corporation

A Data Availability Statement describing where the data can be found is required at submission. Your answers to this question constitute the Data Availability Statement and **will be published in the article**, if accepted.<br><br>**Important:** Stating 'data available on request from the author' is not sufficient. If your data are only available upon request, select 'No' for the first question and explain your exceptional situation in the text box.<br><br>Do the authors confirm that all data underlying the findings described in their manuscript are fully available without restriction?	
**Describe where the data may be found in full sentences. If you are copying our sample text, replace any instances of XXX with the appropriate details.**<br><br>• If the data are **held or will be held in a public repository**, include URLs, accession numbers or DOIs. If this information will only be available after acceptance, indicate this by ticking the box below. For example: *All XXX files are available from the XXX database (accession number(s) XXX, XXX.*).<br>• If the data are all contained **within the manuscript and/or Supporting Information files**, enter the following: *All relevant data are within the manuscript and its Supporting Information files.*<br>• If neither of these applies but you are able to provide **details of access elsewhere**, with or without limitations, please do so. For example:<br><br>*Data cannot be shared publicly because of [XXX]. Data are available from the XXX Institutional Data Access / Ethics Committee (contact via XXX) for researchers who meet the criteria for access to confidential data.*<br><br>*The data underlying the results presented in the study are available from (include the name of the third party*	All data used to conduct this study are provided within the manuscript.

and contact information or URL).<br>• This text is appropriate if the data are owned by a third party and authors do not have permission to share the data.<br><br>* typeset	
Additional data availability information:	

Powered by Editorial Manager® and ProduXion Manager® from Aries Systems Corporation

Manuscript Click here to access/download;Manuscript;Manuscript.docx

Assessing Health Professionals' Adherence to IMCI Protocols for Key Symptoms in Sick Children in Ethiopia: Findings from the 2021-2022 National Service Provision Survey.

[1]Abiyu Abadi Tareke*, [2]Awoke Keleb, [3]Kaleab Mesfin Abera, [4]Natnael Kebede, [5]Endalkachew Mesfin, [6]Aznamariam Ayres, [7]Yawkal Tsega, [8]Abel Endawkie, [9]Shimels Derso Kebede, [10]Eyob Tilahun Abeje, [11]Ermias Bekele Enyew, [12]Chala Daba, [13]Lakew Asmare, [14]Fekade Demeke Bayou, [15]Mastewal Arefaynie & [16]Asnakew Molla Mekonen

[1]Amref health in Africa, COVID-19 vaccine/EPI technical assistant at West Gondar zonal health department, Gondar, Ethiopia

[2&12]Department of Environmental Health College of Medicine and Health Sciences, Wollo University, Dessie, Ethiopia

[4]Department of Health Promotion, School of Public Health, College of Medicine and Health Sciences, Wollo University, Dessie, Ethiopia

[3,5,7 &16]Department of Health System and Management, School of Public Health, College of Medicine and Health Sciences, Wollo University, Dessie, Ethiopia

[9&11]Department of Health Informatics, School of Public Health, College of Medicine and Health Sciences, Wollo University, Dessie, Ethiopia

[6,8,10, 13 & 14]Department of Epidemiology and Biostatistics School of Public Health College of Medicine and Health Science Wollo University, Dessie, Ethiopia.

[15]Department of Reproductive and Family Health, School of Public Health, College of Medicine and Health Sciences, Wollo University, Dessie, Ethiopia

Abiyu Abadi Tareke

Corresponding author (abiyu20010@gmail.com)

Abstract

Background: The Health Services Provision Assessment in Ethiopia (SPA-ET) is a type of survey that generate data related to the availability and quality of health services in Ethiopia. Despite the presence of integrated management of childhood illness protocols in healthcare settings, there has been scare exploration or assessment of how effectively and consistently health professionals are following these guidelines in the context of child healthcare.

Objective: This study aims to identify factors influencing healthcare worker adherence to integrated management of childhood illness protocols to pinpoint areas with high non-adherence rates.

Methods: The data used in this study was collected through the Service Provision Assessment in Ethiopia survey; a nationwide survey conducted from 11 August 2021 to 4 February 2022. It included a total of 788 health professionals who assessed sick children experiencing at least one of the three main childhood illness symptoms: fever, cough, or diarrhea. We employed STATA version 16 for data analysis, utilizing cross-tabulations to explore relationships between variables and logistic regression modeling to identify factors influencing adherence. To account for the hierarchical structure of the health survey data, we employed multilevel logistic regression. Model selection was based on comparison parameters including the Bayesian Information Criterion (BIC) and Akaike Information Criterion (AIC). We computed

adjusted odds ratios with 95% confidence intervals, and statistical significance was determined at a significance level of $p < 0.05$.

Results: The rate of adherence to the integrated management of childhood illness guideline was 33% (95% CI: 29.70%, 36.26%). The analysis revealed several factors influencing adherence to IMCI protocols. child's age [aOR = 0.66, 95% CI: (0.45, 0.87)], facility type [aOR = 2.61, 95% CI: (1.84, 3.37)], place of residency [aOR = 0.54, 95% CI: (0.38, 0.77)], and care provider's qualification [aOR = 1.71, 95% CI: (1.18, 2.48)] were all statistically significant. Moreover, the primary cluster is situated in the west Oromia region, with a central focus at coordinates (7.982108 N, 36.203355 E) and extending to a radius of 78.28 km.

Conclusion: This study confirms a low adherence rate (33%) among health professionals in Ethiopia to the IMCI guideline for assessing the three main symptoms of sick children. The study identified child's age, facility type, academic qualification and place of residency as crucial factors correlated with adherence rate. Furthermore, 5 secondary clusters (hotspot area) were identified using SaTScan software. To address the higher protocol refusal, interventional plan needs to be based on academic qualification of care provider, facility type, Age of child and place of residency. Moreover, interventions to reduce non-adherence of IMCI guideline should be location-tailored based on identified hotspot areas in order to restore Guideline related adherence equality.

Key words: adherence, factor associated, IMCI protocol, Spatial analysis, Ethiopia

Introduction

The Ethiopian health service provision assessment (E-SPA) is a holistic survey mainly conducted to assess the quality and availability of health services at innumerable healthcare

facilities (hospitals, health centers and other private health facilities)(1). The survey brings information regarding the performance of health systems, weakness and strength of the healthcare delivery system, and areas of enhancement. SPA also assesses the quality of healthcare by evaluating adherence of health workers to clinical guidelines such as integrated management of child illness (IMCI), patient management and infection prevention(2).

To assist health workers in effectively diagnosing and treating sick children in laboratories and medical equipment limited countries like Ethiopia, World Health Organization (WHO) and the United Nations Children's Fund (UNICEF) introduced IMCI guideline since 1995(3-6). IMCI has countless potential to advance healthcare providers performance(7, 8) and quality care for sick children(3). In Ethiopia, a country with several healthcare challenges(9), the IMCI protocols have been instrumental in guiding health professionals in the assessment and management of these critical symptoms(8).

This guideline starts with identification of key symptoms of sick children. Among these, the guideline mainly assesses three symptoms which are indicative of a range of common childhood illnesses, including pneumonia, diarrhea, respiratory infections, and other potentially severe conditions. These symptoms can serve as red flags, potentially necessitating hospitalization (10). Childhood illnesses, particularly those presenting with vital symptoms like fever, diarrhea, and cough/difficulty of breathing, are a significant contributor to the global burden of illness and death among children under five.

In 2015, preventable diseases like pneumonia, diarrhea, and malaria tragically claimed the lives of an estimated 16,000 children under five daily (11). Current data in Ethiopia indicates high child mortality rates: 29 neonatal deaths, 48 infant deaths, and 67 under-5 deaths per 1,000 live births (12). Despite this high burden of child mortality and morbidity, surveys conducted in

health facilities across three Ethiopian regions – Amhara, Oromia, and Southern Nations, Nationalities, and Peoples' Region (SNNP) – revealed disappointingly low coverage of IMCI protocols. Amhara exhibited a 20% IMCI coverage, Oromia recorded a mere 4% coverage, and the SNNP Region reported a 25% coverage (13). These findings underscore the urgent need to improve IMCI coverage across all Ethiopian regions, potentially contributing to a reduction in the high child mortality rates observed in the country.

While IMCI has been implemented in all health facilities across Ethiopia, there remains a significant gap in understanding the adherence status of health professionals to this guideline. And there has been limited exploration or assessment of how effectively and consistently health professionals are following these guidelines. Our literature review found no prior research on healthcare worker adherence to IMCI protocols in Ethiopia.

This study is the first to assess healthcare worker adherence to IMCI guidelines for evaluating the three key childhood illness symptoms. To gain a robust understanding of healthcare worker adherence to IMCI protocols across Ethiopia, this research utilizes a comprehensive analysis of the most recent data. Going beyond national-level assessment, the study explores into spatial patterns by employing SaTScanTM software tool. This powerful tool allows for the identification of hotspots with high concentrations of non-adherent healthcare professionals, enabling targeted interventions to improve adherence. By pinpointing hotspot areas, this study offers valuable insights for both national and local health decision-makers. This information allows for targeted interventions to be implemented in specific geographic locations, ultimately improving healthcare worker adherence to IMCI protocol.

Therefore, this study plays a vital role in unmasking IMCI adherence related problems in Ethiopia. By utilizing national representative data from the latest survey and incorporating

advanced statistical techniques, our research provides an accurate assessment of the spatial variation and factors linked to IMCI adherence. The findings serve as a valuable resource for policymakers, aiding them in addressing the specific needs of different regions within the country. Through this study, we aim to contribute significantly to the ongoing efforts in maximizing IMCI guideline implementation coverage in Ethiopia.

Methods and materials

Study design and period

Study area

Ethiopia, the second-most populous country in Africa, boasts a diverse administrative structure that ranges from the highest level of the region to the lowest level of Kebele. Ethiopia is bordered by Sudan and South Sudan on the west, Eritrea and Djibouti on the northeast, Somalia on the east and southeast, and Kenya on the south. The distribution of population between rural and urban communities is unequal. The total fertility rate of the country is 4.6 births per woman. The corresponding crude birth rate was 32 per 1,000 in 2016. The average household size is 4.6, and the population is projected to reach 122.3 million by 2030(2).

Ethiopia's healthcare system has three tiers: primary (health posts, centers, and hospitals), secondary (general hospitals serving about a million people), and tertiary (specialized hospitals for 5 million)(14, 15). While focused on accessibility through primary care, challenges remain in service quality and rural access. The healthcare tier system includes both public and private health facilities. Private health facilities include clinics, diagnostic centers, and pharmacies. The country comprises eleven regions, namely Tigray, Oromia, Afar, Sidama, Afar, Amhara,

Benishangul Gumuz, south west Ethiopia, SNNP, Gambella, and Somali (Figure 1). Additionally, it encompasses two administrative cities, namely Dire-Dawa and Addis Ababa. Each region is further divided into zones, zone into woredas, and woreda to the last administrative structure known as Kebeles (Figure 1).

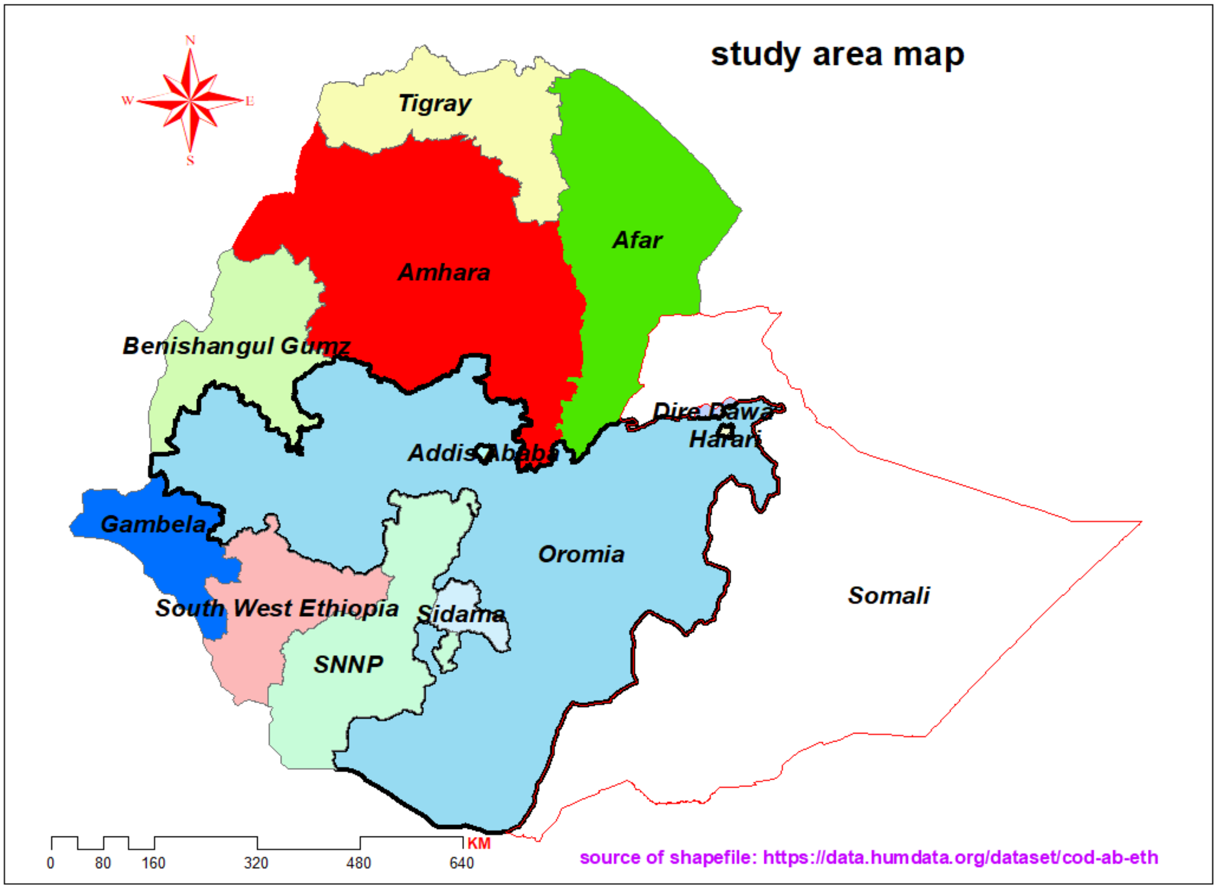

Figure 1: Map of the study area (Ethiopia) in 2021.

Population

Source Population

All healthcare workers in Ethiopia.

Study Population

Healthcare workers who were directly involved in assessment of sick children exhibiting the three main symptoms: fever, diarrhea and cough/difficulty of breathing were included in this study.

Data sources

Data for this study was obtained from the second E-SPA survey which was collected from 11 August 2021 to 4 February 2022. The survey process was led by Ethiopian public health institute (EPHI) and ministry of health (MOH) and financed by United States Agency for International Development (USAID). The main objective of the 2021 E-SPA was to collect information on the availability and delivery of healthcare services in the country. It also examines the readiness of health facilities to provide quality health services. The main focus areas of E-SPA were child health, maternal and newborn care, family planning, sexually transmitted infections (STI), HIV/AIDS, tuberculosis (TB), malaria, non-communicable diseases (NCD) and neglected tropical diseases (NTD) (2).

Study variables

Dependent variable

The outcome variable for this study is: Adherence to IMCI Protocols (yes/no)

IMCI guideline requires each sick child's mother/caregiver to be asked about three main symptoms: cough or difficulty of breathing, diarrhea, and fever. Those three main symptoms information might be gotten from the caregivers as the main complaint or the healthcare professional is required to probe for information about the symptoms.

Adherence was assessed through direct observation of sick child consultations by trained survey interviewers. The interviewers used standardized observation checklists specifically designed to capture adherence to IMCI guidelines for these three key symptoms. But this observational doesn’t include physical examination checklists. Checklists included items about history of three main symptoms for any sick child: 1. Did the health professional asked the caregiver if the child have difficulty of breathing or cough? 2. Did the health professional asked the caregiver if the child have diarrhea? and 3. Did the health professional ask the caregiver if the child have fever?

Adherence: if healthcare provider is actively asking caregivers specific questions about all three symptoms, regardless of whether they are the main reason for the visit.

Non-Adherence: A health professional was classified as non-adherent if they did not perform a specific procedure recommended by the IMCI guidelines for assessing one or more of the three key symptoms.

Independent variables

Following a comprehensive literature review, we selected variables to be included in a multilevel multivariable regression analysis. These variables encompass individual-level factors such as healthcare professional's academic qualification, child's age, sex of both the care provider and child, case type (referral/non-referral), and child's place of residence. Additionally, region and facility type were considered as facility-level factors. These variables were identified as crucial factors potentially influencing adherence to IMCI protocols. Examining them collectively allows for a deeper understanding of these interrelationships.

Sample Size and Sampling Procedures

Sampling frame

The survey utilized a master list from the Ministry of Health, comprising 25,711 functioning health facilities in Ethiopia, including 413 hospitals, 3,556 health centers, 16,841 health posts, and 4,942 private health facilities, as sampling frame.

Sampling procedures

Stratification was realized by first separating the health facilities in each administrative region by facility type. The sample allocation featured a power allocation across regions, to achieve comparable survey precision (to get adequate sample from regions with small sample size). Due to their limited number, all higher clinics and health centers within Harari and Dire Dawa, along with all hospitals nationwide, were included in the study, ensuring a comprehensive analysis in these specific regions. Using power allocation sample features across all regions, 310 health centers, 356 clinics and 328 health posts were selected to be surveyed(2).

Fifteen healthcare providers were interviewed in each health facilities and all healthcare providers were interviewed in facilities having total provider less than fifteen in number. Health facilities having greater than 15 health professionals were sampled randomly (via lottery) method after taking their qualifications, department in which they work, and the services they provide into consideration.

For this study, total of 788 (weighted) health professionals who assessed children who have experienced any of the three main symptoms and have been seen at any of the selected health facilities are included in this study.

Data analysis

Spatial analyses

To explore, create, visualize, and edit the spatial information of IMCI adherence of health workers in Ethiopia, we employed ArcGIS version 10.8 software. For assessing the spatial pattern of health professionals who are non-adherent to IMCI guidelines, we utilized an inferential statistic known as the Spatial Autocorrelation (Global Moran's I) tool (16). This tool calculates the Moran's Index(I) value along with a z-score and p-value to determine whether the pattern of non-adherence is scattered, clustered, or randomly distributed. The significance of Moran’s Index is evaluated based on both the z-score and p-value.

To search for the local hotspot areas (local areas having higher occurrence of non-adherent health professionals to IMCI guideline than expected), purely spatial scan statistics was employed using SaTScan™ version 10 software. The analysis utilized the Bernoulli model-based purely spatial scan statistic with 1/0 event data, where cases (1) represent “**non-adherent”** and controls (0) represent “**adherent”** to follow IMCI guideline while assessing the three main symptoms of childhood illness. We retained the default maximum spatial cluster size setting (50% of the population at risk). This setting is recommended as it allows for the identification of clusters of both small and large size without pre-selection bias regarding cluster size.

Multivariable multilevel analysis

To account for the sharing of similar characteristics at health facilities, we developed a two-level multivariable multilevel logistic regression model that health workers nested in their facility type (hospital, health center and private health facility). Bivariable analyses were

performed to assess the association between IMCI adherence and each exposure variable (one variable at a time) using multilevel logistic regression analysis. Variables that were considered statistically significant or which displayed an association with IMCI adherence in the bivariable models with a P-value of less than 0.25 were included in the final models (multivariable multilevel regression). Exposure variables were checked for multicollinearity using variance inflation factors (VIF). Variables with VIFs greater than 10 were considered to be indicative of multicollinearity and should be removed from the final models(17).

We developed four models to identify factors associated with IMCI adherence. The fully unconditional model (Model 1) was built with no predictors and aimed to describe the components of the total variance in the odds of IMCI adherence and the intra-class correlation (ICC). The next model (Model 2) was adjusted for individual level (level-one) factors that showed bivariable associations with IMCI adherence. The next model (Model 3) was adjusted for the facility-level (level two) factors. The final model (Model 4) was adjusted for both individual-level and facility-level factors. Adjusted odds ratios with 95% confidence intervals (CI) were used as estimates of the association between individual and facility-level factors and the odds of IMCI adherence.

The intra-class correlation was used as a measure of whether the variation in IMCI adherence was primarily between-facilities or within-facilities. ICC is the ratio of the level-two variance to the total variance and takes a value between 0 and 1, with higher values suggesting greater importance of facility-level factors(18) in understanding the individual differences in IMCI adherence. The proportional change in variance (PCV) was used as a measure of the change in variance between different models (19). In this case, PCV was used to measure the total

variation in IMCI adherence that was attributable to individual-level factors and facility-level factors.

Goodness of fit was assessed for each of the four models using Akaike's information criterion (AIC) and Bayesian information criteria (BIC) (20)to select the model with the optimal fit. Lower values of AIC and BIC were indicative of better fit.

Ethical consideration

This study used datasets obtained from the nationally representative Service Provision Assessment (SPA) survey. The survey data used in this study are completely anonymized and cannot be traced back to individual participants, and the original data collection process ensured adherence to ethical standards and obtained appropriate consent from participants. Therefore, additional consent is not required for this study.

Ethical approval was not considered necessary, given that the datasets were requested through a formal process that involved providing a clear explanation of the study's objectives. We submitted a request for the SPA-Ethiopia dataset through the online database (https://dhsprogram.com/). Subsequently, we received authorization in the form of a letter that granted permission to download the requested datasets, ensuring adherence to ethical standards and data usage protocols.

Results

Total of 788 health professionals who assessed sick children Majority of the participants were from Oromia region 300(38%) and followed by SNNP212(27%) (Table 1). The median age of children who participated in this study was 18 months (IQR: 9-31 months).

Table 1: demographic characteristics of study participants (n=788)

Variable	Frequency (weighted sample)	Percent
**Children's age**		
2-24 months	4711	59.84
≥24 months	317	40.16
**Sex of child**		
Male	394	49.91
Female	394	50.09
**Children's place of residency**		
Urban	366	46.42
Rural	422	53.58
**Case type**		
Cold case (not referral)	129	16.41
Referral	659	83.59
**Sex of healthcare provider**		
Male	509	64.51
Female	279	35.49
**Qualification of care provider**		
Nurse	464	58.88
HO	190	24.04
MD	134	17.09
**Facility type**		
Private HF	317	40.14
HC	368	46.76
Hospitals	103	13.10
**Region**		
Afar	3	0.31
Amhara	99	12.58
Oromia	300	37.99
Somali	22	2.83
Benishangul	8	1.03
S.N.N. P	212	26.94
Gambella	8	1.05
Harari	3	0.37
Addis Ababa	70	8.85
Dire Dawa	18	2.30
Sidama	45	5.75

NOTE: HF-health facility, HC- health center, MD- medical doctor, HO- health officer, S.N.N.P- south Nations, Nationalities, and People

The reported adherence rate to IMCI protocols in Ethiopia was 33% [95% CI: 29.7%, 36.3%]. Notably, certain regions exhibit rates significantly above the national average, reflecting varying percentages of adherence toward IMCI guideline. Benishangul Gumuz, Dire-Dawa, Harari, and Gambella stand out with increased rates. Oromia (30%) and Sidama (29%) are among the lowest performing regions (Figure 2).

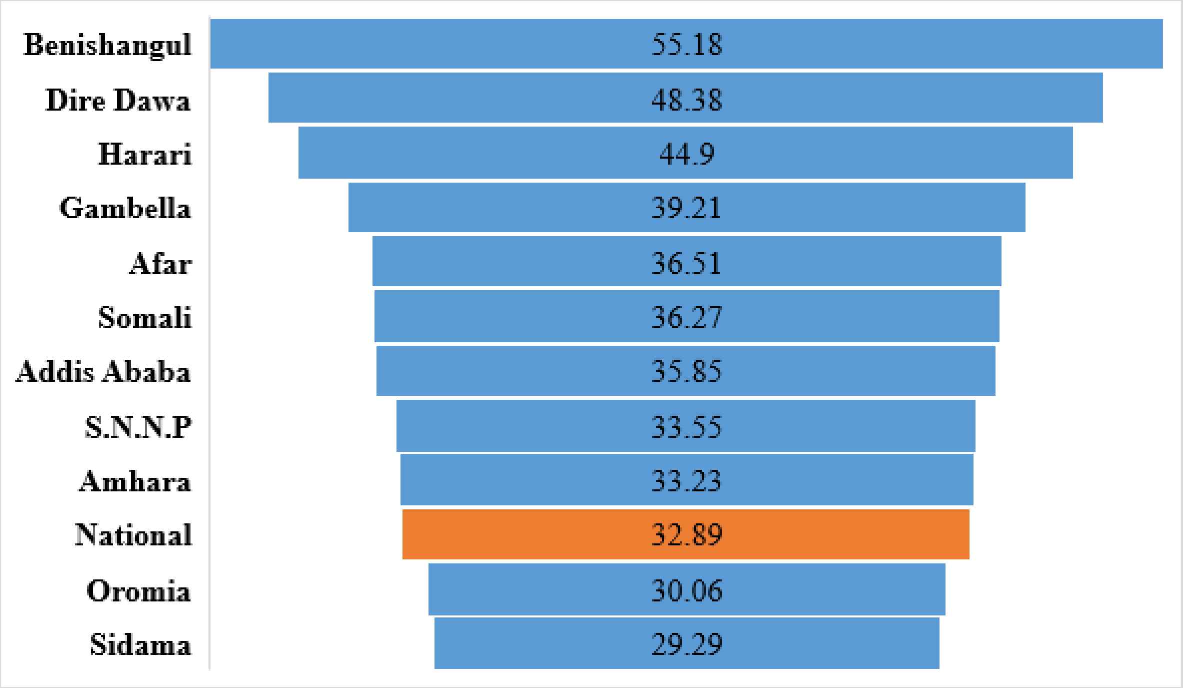

Figure 2: health professionals' adherence rate to IMCI guideline in Ethiopia by administrative region, 2021.

The adherence rate of health workers toward the IMCI guideline is highest during the assessment of fever, specifically at 70%. Adherence is higher, specifically 36%, when assessing children aged 2-24 months compared to those aged ≥ 24 months. Furthermore, a highest percentage of adherence, specifically 44%, was observed when children were assessed by health officers. Similarly, children seen at health centers showed an adherence rate of 42%. (Table 2).

Table 2: adherence rate of health professionals towards IMCI protocols while assessing the three symptoms of sick child in Ethiopia, 2021.

IMCI items	Overall adherence	Age of child		Qualification of the provider			Facility types		
		**2-2424 months**	**≥24 months**	**Nurse**	**HO**	**MD**	**Private HFs**	**HCs**	**Hospitals**
**Fever**	70.55%	73%	64%	63%	83%	80%	65%	73%	77%
**Diarrhea**	50.38%	49%	52%	49%	51%	53%	45%	54%	49%
**Cough or difficulty of breathing**	60.17%	61%	59%	57%	67%	65%	57%	61%	64%
**All 3 symptoms included**	33%	36%	28%	27%	44%	36%	22%	42%	33%

Note: HO- health officer, MD-medical doctor, HFs -health facilities, HC-health centers

Locations of non-adherent health workers

The SaTScan statistics detected clusters categorized into primary and four secondary clusters. A total of 87 statistically significant location IDs (EAs) were found in those categories of clusters. Out of these, 13 EAs were located inside the window of the most likely cluster (a crucial focus point for targeted interventions) and the remaining clusters were classified as secondary clusters. (Table 3).

SaTScan results indicated that primary cluster (a cluster with high LLR, 20.82) mainly converged on west Oromia region (Figure 3), centered at (7.982108 N, 36.203355 E) within a radius of 78.86 km (Table 3). The relative risk 1.55 can be interpreted as the probability of non-adherence in the primary cluster is 55% higher compared to care provider outside the cluster.

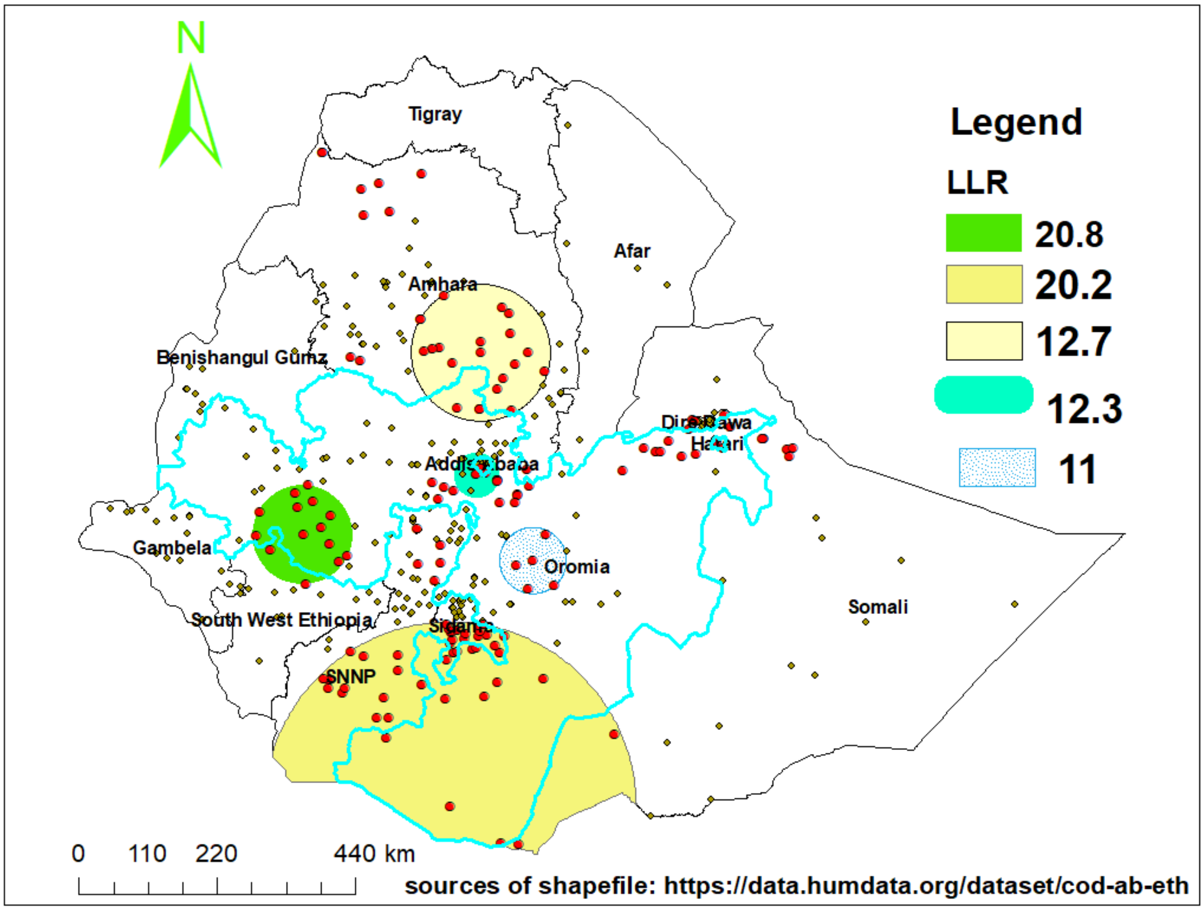

Figure 3: location of statistically significant spatial clustering of healthcare providers which are not adherent to IMCI in Ethiopia, 2021.

Table 3: all clusters of non-adherent health professionals identified through SaTScan in Ethiopia, 2021.

Types of clusters	Coordinates/Radius	No. cluster	Cluster location	Expected cases	Observed cases	p-value
Most likely cluster	(7.982108 N, 36.203355 E) / 78.86 km	13	West Oromia	34	50	0.0000

Secondary cluster 1	(4.076593 N, 38.317550 E) / 295.43 km	44	South Oromia, south-east of SNNP regions	57	80	0.0000
Secondary cluster 2	(10.593845 N, 38.753502 E) / 108.98 km	21	Southern Amhara region	21	31	0.0006
Secondary cluster 3	(8.839652 N, 38.693485 E) / 12.45 km	4	Addis Ababa	20	30	0.0008
Secondary cluster 4	(7.614975 N, 39.500907 E) / 52.73 km	5	Oromia region	18	27	0.0027

NOTE: KM-kilometer, E-east, N-north

Multilevel multivariable logistic regression

Measures of variation

An ICC value of 0.5523 in model 1 indicates that more than half (55.23%) of the variability in the odds of IMCI adherence is attributed to differences between facilities. When individual-level factors were exclusively considered in the model (Model 2), the corresponding ICC decreased to 52.1%, indicating a reduced contribution of between-individual variation to the explained variance (ICC = 0.521). Model 4, which accounted for both individual-level and facility-level factors, demonstrated a nearly identical proportion of total variance in the odds of IMCI explained by between-facility variation compared to Model 3 (ICC = 0.536). Moreover, the PCV in Model 2 indicated that the addition of the predictors explained an increased proportion of variation in the odds of IMCI adherence (PCV = 4.25).

As illustrated in Table 4, the process of selecting the model with the optimal fit was guided by lower values of AIC and BIC, both of which are indicators of model fit. Accordingly, Model 4, characterized by lower AIC and BIC values, emerged as the best-fitted model for this dataset.

Table 4: measure of facility level variation in model 1 to 4.

	Model 1	Model 2	Model 3	Model 4
**Measure of variation for IMCI adherence**				
Facility level variance (SE)	4 (0.86)	4.17 (0.9)	3.79 (0.82)	3.84 (0.85)
ICC (%)	55.23	52.11	53.58	53.89
Explained variance PCV (%)	Ref	4.25	5.25	4
**Model fitness**				
AIC	807	795	815	791
BIC	816	838	882	844

NOTE: SE-standard Error, ICC-intra-class correlation coefficient, PCV-proportional change in variance, AIC-Akaike information criterion, BIC-Bayesian information criterion

Factors associated with adherence

In the realm of multivariable modeling, certain factors emerge as noteworthy contributors to the adherence rate of health workers to the IMCI guideline in Ethiopia. It is worth noting that the age of the child (being ≥24 months), the facility type (specifically, being health professional working in health center), the child's place of residency (in rural areas), and the academic qualification of the care provider all demonstrate themselves as significant variables correlated to adherence to the IMCI guideline.

Regarding relationship between age of children and IMCI adherence, children in the higher age group (≥24 months) show a 34% lower likelihood of being assessed based on IMCI protocols [aOR=0.66, 95% CI: 0.45, 0.87] compared to those in the lower age group. Furthermore, children residing in rural communities face a heightened risk of not being assessed according to the IMCI protocols. Health workers are 36% less likely to adhere to the IMCI protocols [aOR=0.54, 95% CI: 0.38, 0.77] when assessing children in rural areas compared to those in urban areas.

Regarding the academic qualification, a discernible difference was shown. The likelihood of adherence is 71% higher [aOR=1.71, 95% CI: 1.18, 2.48] for health officers compared to the base group of nurses.

Analysis of facility-level factors reveals a significantly higher likelihood of adherence to the IMCI guideline. Specifically, healthcare providers in health centers demonstrate a 2.61 times greater adherence to IMCI protocols [95% CI: 1.84, 3.37] compared to their counterparts in private health facilities (Table 5)

Table 5: The multivariable logistic analysis (model 4) of adherence rate to IMCI protocol among health professionals in Ethiopia, 2021.

Variable	COR (95% CI)	aOR (95% CI)	p-value of aOR
**Child's age in months**			
≤24 months(ref)			
>24 months	0.67 (0.49, 0.91)	**0.66 (0.45, 0.87)**	**0.004**
**facility type**			
Private HF (ref)			
Health center	2.49 (1.77, 3.48)	**2.61 (1.84, 3.37)**	**<0.001**
Hospital	1.72 (1.06, 2.81)	1.35 (0.70, 2.61)	0.405
**Case type**			
None referral (ref)			
Referral	0.85 (0.57, 1.29)	0.91 (0.58, 1.43)	0.78
**Child's Place of residency**			
Urban (ref)			
Rural	0.55 (0.41, 0.75)	**0.54 (0.38, 0.77)**	**<0.001**
**Qualification of care provider**			
Nurse (ref)			
Health officer	2.15 (1.51, 3.06)	**1.71 (1.18, 2.48)**	**0.012**
Medical doctor	1.54 (1.03, 2.32)	1.26 (0.67, 2.37)	0.477
**Sex of provider**			
Male (ref)			
Female	0.76 (0.55, 1.04)	0.89 (0.63, 1.27)	0.545
**Sex of child**			
Male (ref)			
Female	0.79 (0.58, 1.06)	0.75 (0.54, 1.03)	0.076
**Region**			
Afar (ref)			
Amhara	0.46 (0.009, 22.67)	0.47 (0.008, 25)	0.712
Oromia	0.70 (0.015, 32.12)	0.89 (0.017, 45.84)	0.957
Somali	1.12 (0.018, 67.00)	1.07 (0.016, 71.7)	0.974
Benishangul	2.84 (0.033, 243.8)	9.31 (0.094, 919.6)	0.341
S.N.N.P	1.04 (0.022, 49.15)	1.14 (0.021, 60.88)	0.946

Gambella	1.63 (0.02, 123.78)	2.19 (0.025, 187.3)	0.729
Harari	1.71 (0.011, 263.3)	1.81 (0.010, 319.9)	0.821
Addis Ababa	0.89 (0.018, 44.34)	0.55 (0.099, 31.13)	0.775
Dire Dawa	2.28 (0.039, 131.9)	2.59 (0.04, 168.33)	0.654
Sidama	0.84 (0.016, 42.96)	1.18 (0.02, 68.41)	0.934

NOTE: ref- reference, COR-Crude odds ratio, aOR- adjusted odds ratio, HF-health facility, S.N.N.P- south Nations, Nationalities, and People

Discussion

We explore findings from a detailed analysis, including spatial SaTScan, multilevel multivariable analysis, and an examination of IMCI adherence percentages using the national survey data ESPA. These approaches offer a thorough insight into geographic patterns, individual and facility-level determinants, and the overall percentage of IMCI adherence in assessing three childhood illnesses.

According to this study, the adherence of health workers to the IMCI guideline in Ethiopia is low 33% [95% CI: 29.7%, 36.3%]. This prevalence is consistent with research conducted in Tanzania(21) and Kenya(22), which reported a prevalence of 28.4% and 34%, but lower than study done using the SPA data of Nigeria (48%), Tanzania (50%), Uganda (57%) (19%) (22). The variations in IMCI adherence rates may be attributed to differences in socio-demographics, geography, sample sizes, healthcare infrastructure, training programs, and health system disparities between countries. Other contributing factors include the diverse cadres of health workers analyzed in these studies. Additionally, differences may arise from variations in the assessment domains; our study assessed adherence using only the three main symptoms, whereas other studies incorporated danger signs and physical examinations, either separately or in combination with the three main symptoms (22) of childhood illness.

This study identified a significant proportion of healthcare providers showing reluctance to adhere to IMCI protocols, particularly in geographic areas with a higher number of non-compliant health

workers, notably in the Amhara, Afar, SNNP, Sidama, and Oromia regions. This coincides with previous research in Ethiopia which reported inconsistency in the proportion of children correctly assessed according to IMCI guidelines (23). The concentration of non-adherent health professionals in those areas could stem from insufficient training (24), understaffing, high workload, lack of awareness, and inadequate supervision and monitoring by higher officials. We urge future researchers to conduct a thorough and detailed assessment to identify the potential root causes, to identify challenges and specific barriers contributing to non-adherence in those identified hotspots geographic areas. We recommend policy makers and program implementers to integrate evidence-based policy recommendations to address systemic issues contributing to non-adherence.

In this study, there is an observed inverse relationship between the age of sick children and adherence to IMCI guidelines. This finding is consistent with study done using four African countries' SPA datasets(22). Research conducted in Tanzania also suggests a possible bias towards younger children(25). The study found that healthcare providers dedicated more assessment time to children under 5 years old compared to older children. This might be because healthcare providers spent more time assessing younger children than older ones(25). Another reason might be due to work overload(26, 27) or that healthcare providers are less likely to stick to IMCI recommendations for older children, possibly due to assumptions that older children are more resilient to illness or perceived conditions as less severe. Not sticking to the recommendations of the IMCI guideline during assessment of three main sickness in older children (≥24 months) could lead to suboptimal health outcomes like misclassification of diseases, increased costs, additional complications, and prolonged hospital stays. The health governing body in the country should focus on the need for enhanced training and capacity building for healthcare professionals to ensure

that they are equipped with the knowledge and skills required to appropriately manage childhood illnesses in older children.

Another finding of this study is children living in rural settings of Ethiopia face Rural heightened challenges when it comes to adhering to the IMCI guidelines, particularly in comparison to their urban counterparts. Similar finding is also reported by another study done in the united states of America (28) which concluded that rural dwellers are less likely to get preventive health services based on recommended healthcare guidelines. This might be due to that rural-urban divide of skilled healthcare workers. A key challenge in rural healthcare settings in many sub-Saharan countries, as documented evidence shows, is the shortage of skilled health professionals(29, 30). These professionals are concentrated in urban areas compared to rural settings(31). Another justification for this finding might be the lower levels of education in rural areas caregivers (12, 32) may contribute to a lack of awareness and parents' ability to recognize and respond to signs of illness in their children. Addressing education, community awareness, closing the gap of skilled health professional distribution in rural areas related challenges could contribute to narrowing the gap in adherence to IMCI guidelines between rural and urban populations, ultimately improving the health outcomes of children in rural areas.

Our analysis of care provider factors indicates that health officers (HOs) are more likely to adhere to IMCI protocol recommendations when assessing children compared to nurses. One possible reason for this could be that health officer professionals receive more specialized education on IMCI protocols during their professional training at university compared to nurse professionals. According to the Ethiopian curriculum, health officers have a wider scope of practices related to clinical skills, including the comprehensive assessment and management of childhood illnesses(33).

Finally, the facility level factors revealed a significant difference in the likelihood of children receiving clinical services in accordance with IMCI recommendations based on the type of facility visited. Specifically, children who had sought care in health centers demonstrated a notably higher likelihood, nearly 2.61 times more, of receiving clinical services aligned with IMCI guidelines compared to their counterparts who were attended to in private health facilities. This finding is consistent with other studies done in other African countries such in Uganda(34), north-west Ethiopia(35), Nigeria(36, 37), western Kenya (38) which reported that the provision of health services in accordance with their national guidelines is unsatisfactory. It seems possible that these results are due to the major gap of the country in regulating the health workers in private health facilities (39). Other alternative explanations for this result might be that private health facilities may face resource constraints, limiting their ability to invest in staff training, and other resources required for implementation of IMCI recommendations. This finding suggests that targeted intervention specifically to private health facilities is required to ensure that children, regardless of the type of facility, receive clinical services in accordance with IMCI recommendations.

Strengths and limitation of the study

Finally, several strengths and limitations can be pinpointed from this study. The main strength of this study is the utilization of most recent national data to study adherence of health workers to IMCI guideline. The authors of this explored the clustering of health professionals reluctant to adhere to IMCI guideline using the emerging statistical analysis called spatial analysis in the context of health systems. Location specific response to health problems based on identified clusters could help in narrowing health inequalities.

The national survey data utilized in this study omits data of Tigray region. So, the exclusion of Tigray region's data from this study might have limited the generalizability of findings to the entire population of the country. The opportunity to gain insight into the Tigray region about prevalence, contributing factors and hotspot areas of adherence to IMCI protocols are missed and this could impair region-specific policy recommendations. In relation to the cross-sectional aspect, SPA Ethiopia encounters analogous constraints to other cross-sectional surveys i.e., cannot be used to determine the cause of something. Hence, we used data from secondary survey, missing other behavioral and cultural factors in this study could compromise model performance. Finally, this study didn't directly measure healthcare professionals' adherence to established protocols for assessing danger signs and conducting physical examinations in children. While the data hints at areas for improvement, it can't decisively determine overall adherence rates or reasons for deviations from protocols.

This study's observational design introduces limitations. The Hawthorne effect, where healthcare providers modify their behavior when aware of observation, could lead to an overestimation adherence rate to IMCI protocols. Furthermore, our dependence on caregiver recall of symptoms through questioning presents a potential source of bias. Inaccurate or incomplete recall by caregivers might distort the data, hindering a precise assessment of actual adherence practices.

Conclusion

The rate of adherence to IMCI guideline in Ethiopia is low. Low adherence could have various implications, including potential gaps in the quality of child healthcare and the prevention and management of childhood illnesses. By tailoring interventions to the unique needs of each geographic area, this targeted plan aims to create a more equitable distribution of health

professionals adhering to IMCI protocols, ultimately improving child healthcare outcomes across the region. A full implementation of IMCI programs should consider several key factors to ensure comprehensive and effective coverage like the diverse needs and contexts of children, healthcare facilities, and providers.

Additional research is necessary to conduct thorough analyses in hotspot areas and to examine changes in IMCI adherence over time, exploring spatio-temporal relationships. It is advisable to undertake studies comparing hotspot areas with non-hotspot regions to discern any noteworthy differences or similarities influencing variations in adherence. Furthermore, we suggest that future researchers employ qualitative research methods to gather local perspectives on IMCI adherence in hotspot areas, providing nuanced insights into community beliefs and attitudes.

Funding

The authors received no specific funding for this work.

Contributions

AAT, AK, NK, YT, AE, SDK, KMA, ET, EBE, CD, LA, FDB, MA and AM were involved in the design and conception of the study; the analysis, and interpretation of the findings. All the authors read and approved the manuscript starting from the first step up to this.

Abbreviations

AIC: Akaike information criteria, **aOR**: Adjusted Odds Ratio, **BIC**: Bayesian information criteria**,** **ESPA**: Ethiopian Service Provision Assessment, **DIC**: Deviance Information Criterion, **ICC**: Intra-Class Correlation, **LLR**: Log-Likelihood Ratio, **PCV**: Proportion of Variance Change, **CI**: confidence interval, **COR**: Crude Odds Ratio**EA**: enumeration areas,

SNNP: South Nation and Nationalities People, **KM**: Kilometer, **IMCI**: Integrated Management Childhood Illness, **EPHI**: Ethiopian public health institute, MOH: Ministry of Health, **USAID**: United States Agency for International Development, TB: tuberculosis, **NCD:** Non-Communicable Diseases**, NTD:** Neglected Tropical Diseases, **VIF**: variance inflation factors, **HO**: health officer, **MD**: Medical Doctor, **HF**: health facility, **HC**: health center**,** **ID/EA**: cluster ID/ enumeration areas, **IQR**: interquartile range.

Ethics approval and consent to participate

As the authors utilized secondary data from 2021-SPA Ethiopia, consent of participants and ethical approval is not required. But the authors have been granted access to the dataset of 2021 SPA-Ethiopia through registering to https://www.dhsprogram.com/ websites.

Consent for publication

Not applicable

Availability of data and material

All data used to conduct this study are provided within the manuscript.

Competing interests

The authors declares that he has no competing interests

Reference

1. Ministry of Health - MoH/Malawi, ICF International. Malawi Service Provision Assessment 2013-14. Lilongwe, Malawi: MoH/Malawi and ICF International; 2014.
2. Ethiopian Public Health Institute, Ethiopia Ministry of Health, ICF. Ethiopia Service Provision Assessment 2021–22. Addis Ababa, Ethiopia, and Rockville, Maryland, USA: EPHI, MoH and ICF; 2023.

3. Bryce J, Victora CG, Habicht J-P, Black RE, Scherpbier RWJHp, planning. Programmatic pathways to child survival: results of a multi-country evaluation of Integrated Management of Childhood Illness. 2005;20(suppl_1):i5-i17.
4. Black RE, Morris SS, Bryce JJTl. Where and why are 10 million children dying every year? 2003;361(9376):2226-34.
5. Lulseged SJEmj. Integrated management of childhood illness: a review of the Ethiopian experience and prospects for child health. 2002;40(2):187-201.
6. Gove SJBotWHO. Integrated management of childhood illness by outpatient health workers: technical basis and overview. The WHO Working Group on Guidelines for Integrated Management of the Sick Child. 1997;75(Suppl 1):7.
7. Nguyen DTK, Leung KK, McIntyre L, Ghali WA, Sauve RJPo. Does integrated management of childhood illness (IMCI) training improve the skills of health workers? A systematic review and meta-analysis. 2013;8(6):e66030.
8. Abayneh M, Gebremedhin T, Dellie E, Tsehay CT, Atnafu AJIJoP. Improving the Assessment and Classification of Sick Children According to the Integrated Management of Childhood Illness (IMCI) Protocol at Sanja Primary Hospital, Northwest Ethiopia: A Pre-Post Interventional Study. 2020;2020.
9. Assefa Y, Gelaw YA, Hill PS, Taye BW, Van Damme WJG, health. Community health extension program of Ethiopia, 2003–2018: successes and challenges toward universal coverage for primary healthcare services. 2019;15:1-11.
10. Robinson DJAh. The integrated management of childhood illness. 1996;18(6):20-1.
11. Federal Ministry of Health Ethiopia %J FMOH AA, Ethiopia. Health Sector transformation plan, 2015/16-2019/20. 2015.
12. Central Statistical Agency - CSA/Ethiopia, ICF. Ethiopia Demographic and Health Survey 2016. Addis Ababa, Ethiopia: CSA and ICF; 2017.
13. Snow JJAA. Health facility End-line Survey-synthesis report. 2008.
14. Abraha A, Myléus A, Byass P, Kahsay A, Kinsman JJPO. Social determinants of under-5 child health: A qualitative study in Wolkayit Woreda, Tigray Region, Ethiopia. 2019;14(6):e0218101.
15. Ababa FDRoEMoHJMoHA. Health Sector Development Program IV 2010/11-2014/15. 2010.
16. Sheng J, Han X, Zhou H. Spatially varying patterns of afforestation/reforestation and socio-economic factors in China: a geographically weighted regression approach. Journal of Cleaner Production. 2017;153:362-71.
17. Midi H, Sarkar SK, Rana S. Collinearity diagnostics of binary logistic regression model. Journal of Interdisciplinary Mathematics. 2010;13(3):253-67.
18. Merlo J, Chaix B, Yang M, Lynch J, Råstam LJJoE, Health C. A brief conceptual tutorial of multilevel analysis in social epidemiology: linking the statistical concept of clustering to the idea of contextual phenomenon. 2005;59(6):443-9.
19. Merlo J, Chaix B, Ohlsson H, Beckman A, Johnell K, Hjerpe P, et al. A brief conceptual tutorial of multilevel analysis in social epidemiology: using measures of clustering in multilevel logistic regression to investigate contextual phenomena. 2006;60(4):290-7.
20. Vrieze SIJPm. Model selection and psychological theory: a discussion of the differences between the Akaike information criterion (AIC) and the Bayesian information criterion (BIC). 2012;17(2):228.

21. Lange S, Mwisongo A, Mæstad OJSs, medicine. Why don't clinicians adhere more consistently to guidelines for the Integrated Management of Childhood Illness (IMCI)? 2014;104:56-63.
22. Krüger C, Heinzel-Gutenbrunner M, Ali MJBHSR. Adherence to the integrated management of childhood illness guidelines in Namibia, Kenya, Tanzania and Uganda: evidence from the national service provision assessment surveys. 2017;17(1):1-12.
23. Abtew S, Negatou M, Wondie T, Tadesse Y, Alemayehu WA, Tsegaye DA, et al. Poor adherence to the integrated community case management of newborn and child illness protocol in rural Ethiopia. 2022;107(6):1337.
24. Reñosa MD, Dalglish S, Bärnighausen K, McMahon SJGHA. Key challenges of health care workers in implementing the integrated management of childhood illnesses (IMCI) program: a scoping review. 2020;13(1):1732669.
25. Adam T, Manzi F, Schellenberg JA, Mgalula L, de Savigny D, Evans DBJBotWHO. Does the Integrated Management of Childhood Illness cost more than routine care? Results from the United Republic of Tanzania. 2005;83(5):369-77.
26. Adam T, Amorim DG, Edwards SJ, Amaral J, Evans DBJHp, planning. Capacity constraints to the adoption of new interventions: consultation time and the Integrated Management of Childhood Illness in Brazil. 2005;20(suppl_1):i49-i57.
27. Edward A, Dam K, Chege J, Ghee AE, Zare H, Chhorvann CJIJfQiHC. Measuring pediatric quality of care in rural clinics—a multi-country assessment—Cambodia, Guatemala, Zambia and Kenya. 2016;28(5):586-93.
28. Casey MM, Call KT, Klingner JMJAjopm. Are rural residents less likely to obtain recommended preventive healthcare services? 2001;21(3):182-8.
29. Watch BJTSoHiB. Health Workforce in Bangladesh: Who Constitutes the Healthcare System. 2007.
30. Ahmed SM, Hossain MA, RajaChowdhury AM, Bhuiya AUJHrfh. The health workforce crisis in Bangladesh: shortage, inappropriate skill-mix and inequitable distribution. 2011;9(1):1-7.
31. Anyangwe SC, Mtonga CJIjoer, health p. Inequities in the global health workforce: the greatest impediment to health in sub-Saharan Africa. 2007;4(2):93-100.
32. Zhang YJCer. Urban-rural literacy gaps in Sub-Saharan Africa: The roles of socioeconomic status and school quality. 2006;50(4):581-602.
33. Negash FJSIJ. Health Officer: A Physician Assistant Comparable Role in Ethiopia. 2021;8.
34. Mpimbaza A, Babikako H, Rutazanna D, Karamagi C, Ndeezi G, Katahoire A, et al. Adherence to malaria management guidelines by health care workers in the Busoga sub-region, eastern Uganda. 2022;21(1):1-15.
35. Argaw MD, Mavundla TR, Gidebo KDJBHSR. Management of uncomplicated malaria in private health facilities in North-West Ethiopia: a clinical audit of current practices. 2019;19(1):1-14.
36. Mokuolu OA, Ntadom GN, Ajumobi OO, Alero RA, Wammanda RD, Adedoyin OT, et al. Status of the use and compliance with malaria rapid diagnostic tests in formal private health facilities in Nigeria. 2016;15(1):1-11.
37. Udoh E, Oyo-Ita A, Odey F, Effa E, Esu E, Oduwole O, et al. Management of uncomplicated malaria in underfives in private and public health facilities in South-eastern Nigeria: a clinical audit of current practices. 2013;2013.

38. Riley C, Dellicour S, Ouma P, Kioko U, ter Kuile FO, Omar A, et al. Knowledge and adherence to the national guidelines for malaria case management in pregnancy among healthcare providers and drug outlet dispensers in rural, western Kenya. 2016;11(1):e0145616.

39. Dejene D, Yigzaw T, Mengistu S, Ayalew F, Kahsaye M, Woldemariam DJGHR, et al. Exploring health workforce regulation practices and gaps in Ethiopia: a national cross-sectional study. 2019;4(1):1-12.

Revised Manuscript with Track Changes

Assessing Health Professionals' Adherence to IMCI Protocols for Key Symptoms in Sick Children in Ethiopia: Findings from the 2021-2022 National Service Provision Survey.

~~Adherence of Health Professionals to National IMCI Protocols: A Study on Assessing Three Key Symptoms in Sick Children in Ethiopia - Insights from the 2021 National Service Provision Survey~~

[1]Abiyu Abadi Tareke*, [2]Awoke Keleb, [3]Kaleab Mesfin Abera, [4]Natnael Kebede, [5]Endalkachew Mesfin, [6]Aznamariam Ayres, [7]Yawkal Tsega, [8]Abel Endawkie, [9]Shimels Derso Kebede, [10]Eyob Tilahun Abeje, [11]Ermias Bekele Enyew, [12]Chala Daba, [13]Lakew Asmare, [14]Fekade Demeke Bayou, [15]Mastewal Arefaynie & [16]Asnakew Molla Mekonen

[1]Amref health in Africa, COVID-19 vaccine/EPI technical assistant at West Gondar zonal health department, Gondar, Ethiopia

[2&12]Department of Environmental Health College of Medicine and Health Sciences, Wollo University, Dessie, Ethiopia

[4]Department of Health Promotion, School of Public Health, College of Medicine and Health Sciences, Wollo University, Dessie, Ethiopia

[3,5,7 &16]Department of Health System and Management, School of Public Health, College of Medicine and Health Sciences, Wollo University, Dessie, Ethiopia

[9&11]Department of Health Informatics, School of Public Health, College of Medicine and Health Sciences, Wollo University, Dessie, Ethiopia

[6,8,10, 13 & 14]Department of Epidemiology and Biostatistics School of Public Health College of Medicine and Health Science Wollo University, Dessie, Ethiopia.

[15]Department of Reproductive and Family Health, School of Public Health, College of Medicine and Health Sciences, Wollo University, Dessie, Ethiopia

Abiyu Abadi Tareke

Corresponding author (abiyu20010@gmail.com)

Abstract

Background: The Health Services Provision Assessment in Ethiopia (SPA-ET) is a type of survey that generate data related to the availability and quality of health services in Ethiopia. Despite the presence of integrated management of childhood illness protocols in healthcare settings, there has been inadequate exploration or assessment of how effectively and consistently health professionals are following these guidelines in the context of child healthcare.

~~**Background:** The Health Services Provision Assessment in Ethiopia (SPA-ET) is a type of survey that generates data related to the availability and quality of health services in Ethiopia. Despite the presence of IMCI protocols in healthcare settings, there has been limited exploration or assessment of how effectively and consistently health professionals are following these guidelines in the context of child healthcare.~~

Objective: This study aims to identify factors influencing healthcare worker adherence to integrated management of childhood illness protocols to pinpoint areas with high non-adherence rates. ~~The objective of this study is to identify the factors of IMCI adherence and~~

using geographical information systems (GIS) to pinpoint areas with elevated hesitancy rates and discern the factors driving these patterns.

Methods: The data uses in this study was collected through the Service Provision Assessment in Ethiopia survey, a nationwide survey conducted from 11 August 2021 to 4 February 2022The data utilized to analyze this study was based on information collected through Service provision assessment in Ethiopia (SPA-ET) survey conducted in 2021. It included a total of 788 health professionals who assessed sick children experiencing at least one of the three main childhood illness symptoms: fever, cough, or diarrhea. A total of 788 sick children who had at least one of the three main symptoms of childhood illness: fever, cough, or diarrhea were included in this study. We employed STATA version 16 for data analysis, utilizing cross-tabulations to explore relationships between variables and logistic regression modeling to identify factors influencing adherenceSTATA version 16 was used to cross-tabulate and fit the models. To account for the hierarchical structure of the health survey data, we employed multilevel logistic regression. Model selection was based on comparison parameters including the Bayesian Information Criterion (BIC) and Akaike Information Criterion (AIC). To address the hierarchical nature of the health survey data, we employed multilevel logistic regression. Model comparison parameters, including BIC and AIC. We computed adjusted odds ratios with 95% confidence intervals, and statistical significance was determined at a significance level of $p < 0.05$.

Formatted: Font: (Default) Times New Roman, 12 pt, Font color: Auto, Pattern: Clear

Formatted: Font: (Default) Times New Roman, 12 pt, Font color: Auto, Pattern: Clear

Formatted: Font: (Default) Times New Roman, 12 pt, Font color: Auto, Pattern: Clear

Results: The rate of adherence to the integrated management of childhood illness guideline (IMCI) is was 33% (95% CI: 29.70%, 36.26%). Two-third of health professionals failed to assess the main symptoms of childhood sickness in accordance with IMCI recommendations. The analysis revealed several factors influencing adherence to IMCI protocols. Noteworthy

~~factors were associated with adherence rate to IMCI guideline encompass~~ child's age~~d~~ [a~~A~~OR = 0.66, 95% CI: (0.45, 0.87)], ~~health~~ facility type [a~~A~~OR = 2.61, 95% CI: (1.84, 3.37)], place of residency [a~~A~~OR = 0.54, 95% CI: (0.38, 0.77)], and care provider's qualification [a~~A~~OR = 1.71, 95% CI: (1.18, 2.48)] were all statistically significant. Moreover, the primary cluster is situated in the west Oromia region, with a central focus at coordinates (7.982108 N, 36.203355 E) and extending to a radius of 78.28 km.

Conclusion: This study confirms a low adherence rate (33%) among health professionals in Ethiopia to the IMCI guideline for assessing the three main symptoms of sick children. The study identified child's age, ~~types of~~ facility type, academic qualification and place of residency as crucial factors correlated with adherence rate. Furthermore, 5 secondary clusters (hotspot area) were identified using SaTScan software. To address the ~~varying~~ high ~~vaccine~~ protocol refusal, interventional plan needs to be based on academic qualification of care provider, ~~types of health~~ facility type, ~~.~~Age of child and place of ~~dwelling~~residency. Moreover, interventions to reduce non-adherence of IMCI guideline should be location-tailored based on identified hotspot areas in order to restore Guideline related adherence equality.

Key words: adherence, factor associated, IMCI protocol, Spatial analysis, Ethiopia

Introduction

The Ethiopian health service provision assessment (E-SPA) is a holistic survey mainly conducted to assess the quality and availability of health services at innumerable healthcare facilities (hospitals, health centers and other private health facilities)(1). The survey brings information regarding the performance of health systems, weakness and strength of the healthcare delivery system, and areas of enhancement. ~~SPA~~ It also assesses the quality of

healthcare by evaluating adherence of health workers to clinical guidelines such as integrated management of child illness (IMCI), patient management and infection prevention protocol(2).

To assist health workers in effectively diagnosing and treating sick children in laboratories and medical equipment limited countries like Ethiopia, World Health Organization (WHO) and the United Nations Children's Fund (UNICEF) introduced IMCI guideline since 1995(3-6). IMCI has countless potential to advance healthcare providers performance(7, 8) and quality care for sick children(3). In Ethiopia, a country with ~~unique~~ several healthcare challenges(9), the ~~national~~ IMCI protocols have been instrumental in guiding health professionals in the assessment and management of ~~these~~ the three critical symptoms i.e., difficulty of breathing/cough, fever and diarrhea (8).

This guideline starts ~~by~~ with identification of key symptoms of sick children. Among these, the guideline mainly assesses three symptoms which are indicative of a range of common childhood illnesses, including pneumonia, diarrhea, respiratory infections, and other potentially severe conditions. These symptoms can serve as red flags, potentially necessitating hospitalization ~~Those symptoms are general indicators of danger signs which may require hospitalizations~~(10). Childhood illnesses, particularly those presenting with vital symptoms like fever, diarrhea, and cough/difficulty of breathing, are a significant contributor to the global burden of illness and death among children under five. ~~Childhood illnesses, mainly those manifesting through vital symptoms such as fever, diarrhea, and cough/difficulty of breathing, contribute significantly to the global burden of morbidity and mortality among children under the age of five.~~

In 2015, preventable diseases like pneumonia, diarrhea, and malaria tragically claimed the lives of an estimated 16,000 children under five daily ~~In 2015, a staggering reality persisted~~

approximately 16,000 children under the age of five continued to lose their lives every day due to preventable causes: Pneumonia, diarrhea, and malaria (11). Current data in Ethiopia indicates high child mortality rates: 29 neonatal deaths, 48 infant deaths, and 67 under-5 deaths per 1,000 live births Current data in Ethiopia suggests that neonatal mortality is at 29 per 1000 live births, infant mortality is at 48 per 1000 live births, and under-5 mortality is at 67 per 1000 live births(12). Despite this high burden of child mortality and morbidity, surveys conducted in health facilities across three Ethiopian regions – Amhara, Oromia, and Southern Nations, Nationalities, and Peoples' Region (SNNP) – revealed disappointingly low coverage of Integrated Management of Childhood Illness (IMCI) protocols. Amhara exhibited a 20% IMCI coverage, Oromia recorded a mere 4% coverage, and the SNNP Region reported a 25% coverage Surveys conducted in health facilities across three regional states in Ethiopia – Amhara, Oromia, and Southern Nations and Nationalities Peoples' (SNNP) – revealed limited coverage of IMCI in these areas. Amhara exhibited a 20% IMCI coverage, Oromia recorded a 4% coverage, and the South Nations Nationalities People (SNNP) Region reported a 25% coverage(13). Notably, the percentage of trained targeted health workers in these three regions was even lower than the IMCI coverage. These findings underscore the urgent need to improve IMCI coverage across all Ethiopian regions, potentially contributing to a reduction in the high child mortality rates observed in the country.

While IMCI has been implemented in certain all health facilities across Ethiopia, there remains a significant gap in understanding the adherence status of health professionals to this guideline. Despite the presence of IMCI protocols in healthcare settings,And there has been limited exploration or assessment of how effectively and consistently health professionals are following these guidelines in the context of child healthcare. Our literature review found no

prior research on healthcare worker adherence to IMCI protocols in Ethiopia.~~Our comprehensive review of existing literature has not revealed any studies or investigations concerning the adherence of health workers to IMCI protocols in Ethiopia.~~

This study is the first to assess healthcare worker adherence to IMCI guidelines for evaluating the three key childhood illness symptoms. ~~This study is the first study to measure the adherence rate of health professionals toward the recommendation of IMCI guideline during the assessment of the three main symptoms of childhood illness.~~ To gain a robust understanding of healthcare worker adherence to IMCI protocols across Ethiopia, this research utilizes a comprehensive analysis of the most recent data. Going beyond national-level assessment, the study delves into spatial patterns by employing SaTScanTM software tool. This powerful tool allows for the identification of hotspots with high concentrations of non-adherent healthcare professionals, enabling targeted interventions to improve adherence. ~~The comprehensive analysis of the most recent data allows for a robust evaluation of the situation of healthcare workers in following the protocols of IMCI in Ethiopia. This research goes beyond simply assessing the national-level scenario and explores into the spatial pattern of IMCI adherence. By employing a potent tool called SaTScanTM software, this study evaluates hotspots of non-adherent health professionals~~

By pinpointing hotspot areas, this study offers valuable insights for both national and local health decision-makers. This information allows for targeted interventions to be implemented in specific geographic locations, ultimately improving healthcare worker adherence to IMCI protocols.

~~. Identifying hotspot areas could be beneficial for both national and local health decision makers, which allows for location targeted interventions in specific geographic areas and helps for better health resource allocation.~~

Therefore, this study plays a vital role in unmasking IMCI adherence related problems in Ethiopia. By utilizing national representative data from the latest survey and incorporating advanced statistical techniques, our research provides an accurate assessment of the spatial variation and factors linked to IMCI adherence. The findings serve as a valuable resource for policymakers, ~~aiding~~ informing them in addressing the specific needs of different regions within the country. Through this study, we aim to contribute significantly to the ongoing efforts in maximizing IMCI guideline implementation coverage in Ethiopia.

Methods and materials

Study design and period

Study area

Ethiopia, the second-most populous country in Africa, boasts a diverse administrative structure that ranges from the highest level of the region to the lowest level of Kebele. Ethiopia is bordered by Sudan and South Sudan on the west, Eritrea and Djibouti on the northeast, Somalia on the east and southeast, and Kenya on the south. The distribution of population between rural and urban communities is unequal. The total fertility rate of the country is 4.6 births per woman. The corresponding crude birth rate was 32 per 1,000 in 2016. The average household size is 4.6, and the population is projected to reach 122.3 million by 2030(2).

Ethiopia's healthcare system has three tiers: primary (health posts, centers, and hospitals), secondary (general hospitals serving about a million people), and tertiary (specialized hospitals for 5 million)(14, 15). While focused on accessibility through primary care, challenges remain in service quality and rural access. The health system of Ethiopia is composed of a three-tiered, service-delivery structure, with primary (This level consists of health posts, health centers, and primary hospitals), secondary (This level consists of general hospitals and is supposed to provide similar services to those of primary hospitals and serve, on average, one million people), and tertiary-level health care (This is the third tier in the Ethiopian health care system and consists of a specialized hospital that covers a population of approximately 5 million). The healthcare health care tier system includes both public and private health facilities. Private health facilities include clinics, diagnostic centers, and pharmacies.The private health facilities include clinics (all levels), diagnostic centers, pharmacies, and drug stores.

The country Ethiopia comprises eleven regions, namely Tigray, Oromia, Afar, Sidama, Afar, Amhara, Benishangul Gumuz, south west Ethiopia, SNNP, Gambella, and Somali (Figure 1). Additionally, it encompasses two administrative cities, namely Dire-Dawa and Addis Ababa. Each region is further divided into zones, zones into woredas, and finally, woredas and to the last administrative structure known as Kebeles (Figure 1).

Formatted: Default Paragraph Font

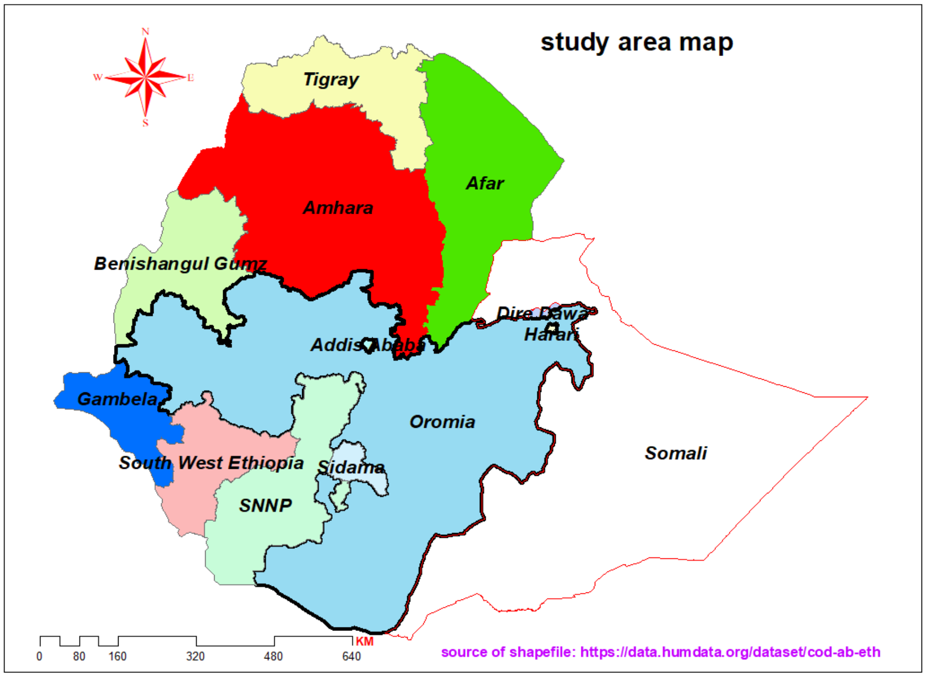

Figure 1: Map of the study area (Ethiopia) in 2021.

Population

Source Population

All healthcare ~~health care~~ workers in Ethiopia.

Study Population

Healthcare workers who were directly involved in assessment of sick children exhibiting the three main symptoms: fever, diarrhea and cough/difficulty of breathing were included in this study.

Data sources

Data for this study was obtained from the second E-SPA survey which was collected from 11 August 2021 to 4 February 2022. The survey process was led by Ethiopian public health institute (EPHI) and ministry of health (MOH) and financed by United States Agency for International Development (USAID). The main objective of the 2021 E-SPA was to collect information on the availability and delivery of healthcare services in the country. It also examines the readiness of health facilities to provide quality health services. The main focus areas of E-SPA were child health, maternal and newborn care, family planning, sexually transmitted infections (STI), HIV/~~ and ~~AIDS, tuberculosis (TB), malaria, ~~and other diseases such as ~~non-communicable diseases (NCD) and neglected tropical diseases (NTD) (2).

Study variables

Dependent variable

The outcome variable for this study is: Adherence to IMCI Protocols (yes/no)

IMCI guideline requires each sick child's mother/caregiver ~~should ~~to be ~~checked ~~asked ~~for~~ about three main symptoms: cough or difficulty of breathing, diarrhea, and fever. Those three main symptoms information might be gotten from the ~~caretaker ~~caregivers as the main complaint or the healthcare professional ~~may ~~is required to probe for information about the symptoms.

Adherence was assessed through direct observation of sick child consultations by trained survey interviewers. The interviewers used standardized observation checklists specifically designed to capture adherence to IMCI guidelines for these three key symptoms. But this observational doesn't include physical examination checklists. Checklists included items about

Formatted: Normal

Formatted: Font:

Formatted: Font: Not Bold

Formatted: Font:

Formatted: Font: Not Bold

Formatted: Font:

history of three main symptoms for any sick child: 1. did the health professional asked the caregiver if the child have difficulty of breathing or cough? 2. did the did the health professional asked the caregiver if the child have diarrhea? And 3. did the health professional asked the caregiver if the child have fever?

Adherence: if healthcare provider is actively asking caregivers specific questions about all three symptoms, regardless of whether they are the main reason for the visit.

Non-Adherence: A health professional was classified as non-adherent if they did not perform a specific procedure recommended by the IMCI guidelines for assessing one or more of the three key symptoms.

Formatted: Font: Bold

Formatted: Justified, Indent: Left: 0.25", Space Before: 0 pt, After: 8 pt, No bullets or numbering

Formatted: Font:

Formatted: Font: Bold

In this study health professionals who assessed all the three main symptoms are put under the categories of "adherent to IMCI guideline" and those who failed to assess all the three symptoms are categorized as "non-adherent to IMCI guideline".

Independent variables

Following a comprehensive literature review, we selected variables to be included in a multilevel multivariable regression analysis. These variables encompass individual-level factors such as healthcare professional's academic qualification, child's age, sex of both the care provider and child, case type (referral/non-referral), and child's place of residence. Additionally, region and facility type were considered as facility-level factors. These variables were identified as crucial factors potentially influencing adherence to IMCI protocols. Examining them collectively allows for a deeper understanding of these interrelationships.

Formatted: Default Paragraph Font, Font color: Black

Formatted: Normal

Formatted: Default Paragraph Font, Font color: Black

Formatted: Default Paragraph Font, Font color: Black

Formatted: Font color: Black

~~Following an extensive literature review, the variables selected for inclusion in the multilevel multivariable regression analysis encompass health professional's academic qualification, child's age, sex of care provider, sex of child, types of case (referral/non-referral), and children of place of residency as individual-level factors. And region and facility types are considered as facility level factors. These variables were identified as crucial factors, drawing on existing research and literature, and examined collectively to gain a comprehensive understanding of their potential influence on the adherence to IMCI.~~

Sample Size and Sampling Procedures

Sampling frame

The survey utilized a master list from the Ministry of Health, comprising 25,711 functioning health facilities in Ethiopia, including 413 hospitals, 3,556 health centers, 16,841 health posts, and 4,942 private health facilities, as ~~our~~ sampling frame.

Sampling procedures

Stratification was realized by first separating the health facilities in each administrative region by facility type. The sample allocation featured a power ~~allocation across~~allocation across regions, to achieve comparable survey precision (to get adequate sample from regions with small sample size). Due to their limited number, all higher clinics and health centers within Harari and Dire Dawa, along with all hospitals nationwide, were included in the study, ensuring a comprehensive analysis in these specific regions. ~~All hospitals in Ethiopia, whole higher clinics and entire health centers in Harari and Dire Dawa were included in the study because of their small number.~~ Using power allocation sample features across all regions, 310 health centers, 356 clinics and 328 health posts were selected to be surveyed(2).

Fifteen hHealthcare providers were interviewed from in each health facilities and all healthcare providers were interviewed in facilities having total provider less than fifteen in number. Health facilities having greater than 15 health professionals were sampled randomly (via lottery) method after taking their qualifications, department in which they work, and the services they provide into consideration.

For this study, total of 788 (weighted) health professionals who assessed children who have experienced any of the three main symptoms and have been seen at any of the selected health facilities are included in this study. Finally, a total of 788 (weighted) children who have experienced any of the three main symptoms and have been seen at any of the selected health facilities are included in this study.

Formatted: Font: (Default) Times New Roman, Font color: Black, Pattern: Clear

Formatted: Font: (Default) Times New Roman, Font color: Black, Pattern: Clear

Formatted: Font: (Default) Times New Roman, Font color: Black, Pattern: Clear

Data analysis

Formatted: Heading 2

Spatial analyses

To explore, create, visualizevisualize, and edit the spatial information of IMCI adherence of health workers in Ethiopia, we employed ArcGIS version 10.8 software. For assessing the spatial pattern of health professionals who are non-adherent to IMCI guidelines, we utilized an inferential statistic known as the Spatial Autocorrelation (Global Moran's I) tool (16). This tool calculates the Moran's Index(I) value along with a z-score and p-value to determine whether the pattern of non-adherencevaccine hesitancy is scattered, clustered, or randomly distributedrandom. The significance of Moran's Index is evaluated based on both the z-score and p-value.

But, toTo search for the local hotspot areas (local areas having higher occurrence of non-adherent health professionals to IMCI guideline than expected), purely spatial scan statistics

~~purely spatial analysis~~ was employed using SaTScan™ version 10 software. The analysis utilized the Bernoulli model-based purely spatial scan statistic with 1/0 event data, where cases (1) represent “**non-adherent”** and controls (0) represent “**adherent”** to follow IMCI guideline while assessing the three main symptoms of childhood illness. We retained the default maximum spatial cluster size setting (50% of the population at risk). This setting is recommended as it allows for the identification of clusters of both small and large size without pre-selection bias regarding cluster size.

Multivariable multilevel analysis

To account for the sharing of similar characteristics at health facilities, we developed a two-level multivariable multilevel logistic regression model that health workers nested in their ~~health~~ facility type (hospital, health center and private health facility). ~~Bivariate~~ Bivariable analyses were performed to assess the association between IMCI adherence and each exposure variable (one variable at a time) using multilevel logistic regression ~~models~~analysis. Variables that were considered statistically significant or which displayed an association with IMCI adherence in the bivariable models with a P-value of less than 0.25 were included in the final models (multivariable multilevel regression). Exposure variables were checked for multicollinearity using variance inflation factors (VIF). Variables with VIFs greater than 10 were considered to be indicative of multicollinearity and ~~were~~ should be removed from the final models(17).

We developed four models to identify factors associated with IMCI adherence. The fully unconditional model (Model 1) was built with no predictors and aimed to describe the components of the total variance in the odds of IMCI adherence and the intra-class correlation (ICC). The next model (Model 2) was adjusted for individual level (level-one) factors that

showed bivariable associations with IMCI adherence. The next model (Model 3) was adjusted for the facility-level (level two) factors. The final model (Model 4) was adjusted for both individual-level and facility-level factors. Adjusted odds ratios with 95% confidence intervals (CI) were used as estimates of the association between individual and facility-level factors and the odds of IMCI adherence.

~~We developed four models to identify the determinants of IMCI adherence. The fully unconditional model (Model 1) was built with no predictors and aimed to describe the components of the total variance in the odds of IMCI adherence and the intra-class correlation (ICC). The next model (Model 2) was adjusted for individual level (level one) factors that showed bivariable associations with IMCI adherence. The next model (Model 3) was adjusted for the individual level (level two) factor of mother's country of birth. This was the only maternal level variable included in our study. All other variables could vary between newborns. The final model (Model 4) was adjusted for both newborn level and maternal level factors. This model included all the variables in Model 2 and Model 3. Adjusted odds ratios with 95% confidence intervals (CI) were used as estimates of the association between individual and facility level factors and the odds of IMCI adherence. All analyses were performed in Stata 16(18).~~

~~Measures of random effects t~~The intra-class correlation was used as a measure of whether the variation in IMCI adherence was primarily between-facilities or within-facilities. ICC is the ratio of the level-two variance to the total variance and takes a value between 0 and 1, with higher values suggesting greater importance of facility-level factors(19) in understanding the individual differences in IMCI adherence. The proportional change in variance (PCV) was used as a measure of the change in variance between different models (20). In this case, PCV

was used to measure the total variation in IMCI adherence that was attributable to individual-level factors and facility-level factors.

~~Goodness of fit~~ Goodness of fit was assessed for each of the four models using Akaike's information criterion (AIC) and Bayesian information criteria (BIC) (21)to select the model with the optimal fit. Lower values of AIC and BIC were indicative of better fit.

Ethical consideration

This study ~~made~~ used ~~of~~ datasets obtained from the nationally representative Service Provision Assessment (SPA) survey. The survey ~~secondary~~ data used in this study are completely anonymized and cannot be traced back to individual participants, and the original data collection process ensured adherence, to ethical standards and obtained appropriate consent from participants.~~confidentiality and ethical standards.~~ Therefore, additional consent is not required for this study~~additional consent is not be necessary for this study~~. Ethical approval was not considered necessary, given that the datasets were requested through a formal process that involved providing a clear explanation of the study's objectives. We ~~The researchers registered their study and~~ submitted a request for the SPA-Ethiopia dataset through the online database (https://dhsprogram.com/). Subsequently, ~~they received~~we received authorization in the form of a letter that granted permission to download the requested datasets, ensuring adherence to ethical standards and data usage protocols.

Results

~~A~~ T~~t~~otal of 788 health professionals who assessed sick children ~~who were assessed for at least one the main three symptoms of childhood illness (fever, cough and diarrhea) were included in this study.~~ Majority of the participants were from Oromia region ~~1528~~300(38~~41~~%) and

Formatted: Font: (Default) Times New Roman, Font color: Auto, Pattern: Clear

Formatted: Font: (Default) Times New Roman, 12 pt, Font color: Auto, Pattern: Clear

followed by ~~Amhara region~~SNNP212~~ 740~~(27~~0~~%) (Table 1). The median age of children who participated in this study ~~is ~~was 18 months (IQR: 9-31 months).

Table 1: demographic characteristics of study participants (n=788)

Variable	Frequency (weighted sample)	Percent
**Children's age**		
2-24 months	4711~~2329~~	59.84~~63.96~~
≥24 months	317~~1312~~	40.16~~36.04~~
**Sex of child**		
Male	394~~2021~~	49.91~~55.51~~
Female	394~~1620~~	50.09~~44.49~~
**Children's place of residency**		
Urban	366~~1718~~	46.42~~47.20~~
Rural	422~~1923~~	53.58~~52.80~~
**Case type**		
Cold case (not referral)	~~596~~129	~~16.44~~16.41
Referral	~~3032~~659	~~83.56~~83.59
**Sex of healthcare provider**		
Male	509~~2590~~	64.51
Female	279~~1051~~	35.49~~71.13~~
**Qualification of care provider**		~~28.87~~
Nurse	464~~2232~~	58.88~~61.32~~
HO	190~~914~~	24.04~~25.10~~
MD	134~~494~~	17.09~~13.58~~
**Facility type**		
Private HF	317~~1246~~	40.14~~34.21~~
HC	368~~2035~~	46.76~~55.90~~
Hospitals	103~~260~~	13.10~~9.88~~
**Region**		
Afar	3~~72~~	0.31~~1.98~~
Amhara	99~~740~~	12.58~~20.32~~
Oromia	300~~1528~~	37.99~~41.96~~
Somali	22~~187~~	2.83~~5.15~~
Benishangul	8~~96~~	1.03~~2.65~~
S.N.N. P	212~~571~~	26.94~~15.69~~
Gambella	8~~75~~	1.05~~2.07~~
Harari	3~~18~~	0.37~~0.5~~
Addis Ababa	70~~237~~	8.85~~6.51~~
Dire Dawa	18~~29~~	2.30~~0.78~~
Sidama	45~~87~~	5.75~~2.38~~

Formatted ...
Formatted ...
Formatted ...
Formatted ...
Formatted Table ...
Formatted ...
Formatted ...
Formatted ...
Formatted ...
Formatted Table ...
Formatted ...
Formatted ...
Formatted ...
Formatted ...
Formatted ...
Formatted ...
Formatted ...
Formatted Table ...
Formatted ...
Formatted ...
Formatted ...
Formatted ...
Formatted ...
Formatted ...
Formatted ...
Formatted ...
Formatted ...
Formatted ...
Formatted Table ...
Formatted ...
Formatted ...
Formatted ...
Formatted ...
Formatted ...
Formatted ...
Formatted ...
Formatted Table ...
Formatted ...
Formatted ...
Formatted ...
Formatted ...
Formatted ...
Formatted ...
Formatted ...
Formatted ...
Formatted ...
Formatted ...
Formatted Table ...
Formatted ...
Formatted ...
Formatted ...
Formatted ...
Formatted ...

NOTE: HF-health facility, HC- health center, MD- medical doctor, HO- health officer, S.N.N.P- south Nations, Nationalities, and People

The reported adherence rate to IMCI protocols in Ethiopia is was 33% [95% CI: 29.7%, 36.3%]., with a 95% confidence interval ranging from 29.7% to 36.3%. Notably, certain regions exhibit rates significantly above the national average, reflecting varying percentages of adherence toward IMCI guideline. Benishangul Gumuz, Dire-Dawa, Harari, and Gambella stand out with increased rates. Oromia (30%) and Sidama (29%) are among the lowest performing regions (Figure 2).

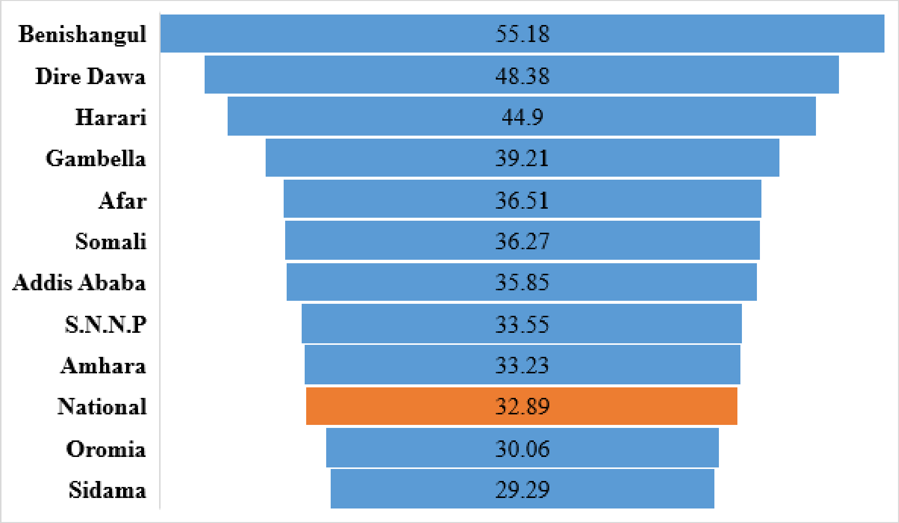

Figure 2: health professionals' aAdherence rate to IMCI guideline in Ethiopia by administrative region, 2021.

The adherence rate of health workers toward the IMCI guideline is highest during the assessment of fever, specifically at 70%. Adherence is higher, specifically 36%, when assessing children aged 2-24 months compared to those aged ≥ 24 months. Furthermore, a

Formatted: Font: Not Bold

Formatted: Font: (Default) Times New Roman, Font color: Auto, Pattern: Clear

Formatted: Indent: Left: 0.25"

highest percentage of adherence, specifically 44%, was observed when children were assessed by health officers. Similarly, children seen at health centers showed an adherence rate of 42%. ~~Health workers adherence rate toward IMCI guideline is found is higher during assessment of fever i.e., 70%. Higher percentage of adherence is followed during assessing children aged ≤ 24 months (36%), among children assessed by health officers (44%) and children seen at health centers (42%)~~ (Table 2).

Table 2: a~~A~~dherence rate of health professionals towards IMCI protocols while assessing the three symptoms of sick child in Ethiopia, 2021.

IMCI items	Overall adherence	Age of child		Qualification of the provider			Facility t~~T~~ypes ~~of HF~~		
		2-24~~≤~~24 months	≥~~>~~24 months	Nurse	HO	MD	Private HFs	HCs	Hospitals
**Fever**	70.55%	73%	64%	63%	83%	80%	65%	73%	77%
**Diarrhea**	50.38%	49%	52%	49%	51%	53%	45%	54%	49%
**Cough or difficulty of breathing**	60.17%	61%	59%	57%	67%	65%	57%	61%	64%
**All 3 symptoms included ~~one~~**	33%	36%	28%	27%	44%	36%	22%	42%	33%

Note: HO- health officer, MD-medical doctor, HFs -health facilities, HC-health centers

Locations of non-adherent health workers

The SaTScan statistics detected clusters categorized into primary and four secondary clusters. A total of 87 statistically significant location IDs (EAs) were found in those categories of clusters. Out of these, 13 EAs were located inside the window of the most likely cluster (a crucial focus point for targeted interventions) and the remaining clusters were classified as secondary clusters. (Table 3).

SaTScan results indicated that primary cluster~~s~~ (a cluster with high LLR, 20.82) mainly converged on west Oromia region (Figure 3), centered at (7.982108 N, 36.203355 E) within a radius of 78.86 km (Table 3). The relative risk 1.55~~, as depicted in table 3,~~ can be interpreted as the probability of non-adherence in the primary cluster is 55% higher compared to care provider outside the cluster.

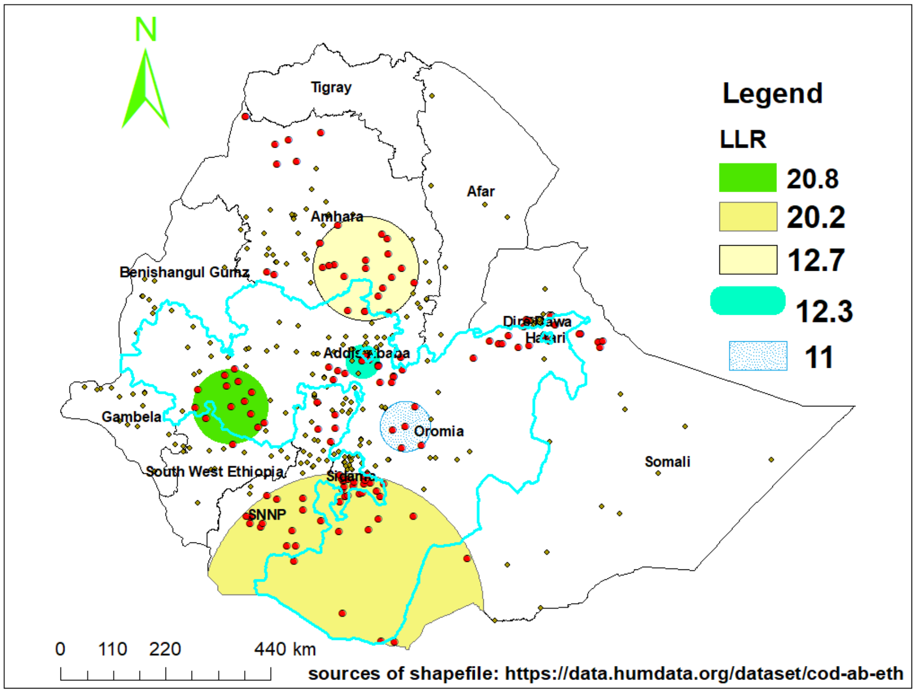

Figure 3: location of statistically significant spatial clustering of ~~health care~~healthcare providers which are not adherent to IMCI in Ethiopia, 2021.

Table 3: all clusters of non-adherent health professionals identified through SaTScan in Ethiopia, 2021.

Types of clusters	Coordinates/Radius	No. cluster	Cluster location	Expected cases	Observed cases	p-value
Most likely cluster	(7.982108 N, 36.203355 E) / 78.86 km	13	West Oromia	34	50	0.0000~~0027~~
Secondary cluster 1	(4.076593 N, 38.317550 E) / 295.43 km	44	South Oromia, south-east of SNNP regions	57	80	0.0000~~0045~~
Secondary cluster 2	(10.593845 N, 38.753502 E) / 108.98 km	21	Southern Amhara region	21	31	0.0006~~56~~
Secondary cluster 3	(8.839652 N, 38.693485 E) / 12.45 km	4	Addis Ababa	20	30	0.0008~~2~~
Secondary cluster 4	(7.614975 N, 39.500907 E) / 52.73 km	5	Oromia region	18	27	0.0027

NOTE: KM-kilometer, E-east, N-north

Formatted: Indent: Left: 0.25"

Multilevel multivariable logistic regression

Measures of variation

An ICC value of 0.5523 in model 1 indicates that more than half (55.23%) of the variability in the odds of IMCI adherence ~~odds~~ is attributed to differences between facilities. When individual-level factors were exclusively considered in the model (Model 2), the corresponding ICC decreased to 52.1%, indicating a reduced contribution of between-individual variation to the explained variance (ICC = 0.521). Model 4, which accounted for both individual-level and facility-level factors, demonstrated a nearly identical proportion of total variance in the odds of IMCI explained by between-facility variation compared to Model 3 (ICC = 0.536). Moreover, the PCV in Model 2 indicated that the addition of the predictors explained an increased proportion of variation in the odds of IMCI adherence (PCV = 4.25).

As illustrated in Table 4, the process of selecting the model with the optimal fit was guided by lower values of AIC ~~(Akaike Information Criterion)~~ and BIC~~ (Bayesian Information Criterion)~~, both of which are indicators of model fit. Accordingly, Model 4, characterized by lower AIC and BIC values, emerged as the best-fitted model for this dataset.

Table 4: m~~M~~easure of facility level variation in model 1 to 4.

	Model 1	Model 2	Model 3	Model 4
**Measure of variation for IMCI adherence**				
Facility level variance (SE)	4 (0.86)	4.17 (0.9)	3.79 (0.82)	3.84 (0.85)
ICC (%)	55.23	52.11	53.58	53.89
Explained variance PCV (%)	Ref	4.25	5.25	4
**Model fitness**				
AIC	807	795	815	791
BIC	816	838	882	844

NOTE: SE-standard Error, ICC-intra-class correlation coefficient, PCV-proportional change in variance, AIC-Akaike information criterion, BIC-Bayesian information criterion

Formatted: Indent: Left: 0.25"

Factors associated with adherence

In the realm of multivariable modeling, certain factors emerge as noteworthy contributors to the adherence rate of health workers to the IMCI guideline in Ethiopia. It is worth noting that the age of the child (being ≥~~over~~ 24 months), the ~~type of health facilit~~facility type ~~y~~ (specifically, being health professional working in health center~~s~~), the child's place of residency (in rural areas), and the academic qualification of the care provider all demonstrate themselves as significant variables correlated to adherence to the IMCI guideline.

Regarding relationship between age of children and IMCI adherence, children in the higher age group (≥~~>~~24 months) show a 34% lower likelihood of ~~adherence~~ being assessed based on IMCI protocols [a~~A~~OR=0.66, 95% CI: 0.45, 0.87] compared to those in the lower age group. Furthermore, children residing in rural communities face a heightened risk of not being assessed

according to the IMCI protocols. Health workers are 36% less likely to adhere to the IMCI protocols [a~~A~~OR=0.54, 95% CI: 0.38, 0.77] when assessing children in rural areas compared to those in urban areas.

Regarding the academic qualification, a discernible difference was shown. The likelihood of adherence is 71% higher [a~~A~~OR=1.71, 95% CI: 1.18, 2.48] for health officers compared to the base group of nurses.

Analysis of facility-level factors reveals a significantly higher likelihood of adherence to the IMCI guideline. Specifically, healthcare providers in health centers demonstrate a 2.61 times greater adherence to IMCI protocols [95% CI: 1.84, 3.37] compared to their counterparts in private health facilities (Table 5)

Table 5: The multivariable logistic analysis (model 4) of adherence rate to IMCI protocol among health professionals in Ethiopia, 2021.

~~displays the results of the multivariable logistic analysis of adherence rate to IMCI protocol among health professionals in Ethiopia, 2021.~~

~~**Table 5**: multilevel multivariable logistic regression results model 4.~~

Variable	COR (95% CI)	a~~A~~OR (95% CI)	p-value of a~~A~~OR
**Child's age in months**			
≤24 months(ref)			
>24 months	0.67 (0.49, 0.91)	**0.66 (0.45, 0.87)**	**0.004**
~~**Health**~~ **facility type**			
Private HF (ref)			
Health center	2.49 (1.77, 3.48)	**2.61 (1.84, 3.37)**	**<0.001~~0~~**
Hospital	1.72 (1.06, 2.81)	1.35 (0.70, 2.61)	0.405
**Case type**			
None referral (ref)			
Referral	0.85 (0.57, 1.29)	0.91 (0.58, 1.43)	0.78
**Child's Place of residency**			

Urban (ref)			
Rural	0.55 (0.41, 0.75)	**0.54 (0.38, 0.77)**	**<0.0010**
**Qualification of care provider**			
Nurse (ref)			
Health officer	2.15 (1.51, 3.06)	**1.71 (1.18, 2.48)**	**0.012**
Medical doctor	1.54 (1.03, 2.32)	1.26 (0.67, 2.37)	0.477
**Sex of provider**			
Male (ref)			
Female	0.76 (0.55, 1.04)	0.89 (0.63, 1.27)	0.545
**Sex of child**			
Male (ref)			
Female	0.79 (0.58, 1.06)	0.75 (0.54, 1.03)	0.076
**Region**			
Afar (ref)			
Amhara	0.46 (0.009, 22.67)	0.47 (0.008, 25)	0.712
Oromia	0.70 (0.015, 32.12)	0.89 (0.017, 45.84)	0.957
Somali	1.12 (0.018, 67.00)	1.07 (0.016, 71.7)	0.974
Benishangul	2.84 (0.033, 243.8)	9.31 (0.094, 919.6)	0.341
S.N.N.P	1.04 (0.022, 49.15)	1.14 (0.021, 60.88)	0.946
Gambella	1.63 (0.02, 123.78)	2.19 (0.025, 187.3)	0.729
Harari	1.71 (0.011, 263.3)	1.81 (0.010, 319.9)	0.821
Addis Ababa	0.89 (0.018, 44.34)	0.55 (0.099, 31.13)	0.775
Dire Dawa	2.28 (0.039, 131.9)	2.59 (0.04, 168.33)	0.654
Sidama	0.84 (0.016, 42.96)	1.18 (0.02, 68.41)	0.934

NOTE: ref- reference, COR-Crude odds ratio, aAOR- adjusted odds ratio, HF-health facility, S.N.N.P- south Nations, Nationalities, and People

Formatted: Indent: Left: 0.25"

Discussion

We explore findings from a detailed analysis, including spatial SaTScan, multilevel multivariable analysis, and an examination of IMCI adherence percentages using the national survey data ESPA. These approaches offer a thorough insight into geographic patterns, individual and facility-level determinants, and the overall percentage of IMCI adherence in assessing three childhood illnesses.

Formatted: Indent: Left: 0.25"

According to this study, the adherence of health workers to the IMCI guideline in Ethiopia is was low (33%)[95% CI: 29.7%, 36.3%]., with a 95% confidence interval of [29.7, 36.3]. This prevalence is consistent with research conducted in Tanzania(22) and Kenya(23), which reported a prevalence of 28.4% and 34%, but lower than study done using the SPA data of Nigeria Namibia (48%), Tanzania (50%), and Uganda (57%) (19%) (23). The variations in IMCI adherence rates may be attributed to differences in socio-demographics, geography, sample sizes, healthcare infrastructure, training programs, and health system disparities between countries. Other contributing factors might include the diverse cadres of health workers analyzed in these studies. Additionally, differences may arise from variations in the assessment domains; our study assessed adherence using only the three main symptoms, whereas other studies incorporated danger signs and physical examinations, either separately or in combination with the three main symptoms (22) of childhood illness. Additionally, differences in results may arise from variations in how studies assess illness. Our study assessed adherence to treatment using only the three main symptoms of the illness. Whereas other studies incorporated danger signs and physical examinations, either separately or in combination with the three main symptoms of childhood illness.

This study identified a significant proportion of healthcare providers showing reluctance to adhere to IMCI protocols, particularly in geographic areas with a higher number of non-compliant health workers, notably in the Amhara, Afar, SNNP, Sidama, and Oromia regions. This coincides with previous research in Ethiopia which reported inconsistency in the proportion of children correctly assessed according to IMCI guidelines (24). The concentration of non-adherent health professionals in those areas could stem from insufficient training (25), understaffing, high workload, lack of awareness, and inadequate supervision and monitoring

Formatted: Font:

by higher officials. We strongly encourage future researchers to carry out a comprehensive and meticulous evaluation aimed at identifying the underlying causes for non-adherence in the geographic areas that have been identified as hotspots. It is crucial to pinpoint the challenges and specific obstacles that contribute to this issue. ~~We urge future researchers to conduct a thorough and detailed assessment to identify the potential root causes, to identify challenges and specific barriers contributing to non-adherence in those identified hotspots geographic areas.~~ We recommend policy makers and program implementers to ~~install~~ integrate evidence-based policy recommendations to address systemic issues contributing to non-adherence.

Formatted: Font: (Default) Times New Roman, 12 pt, Font color: Auto, Pattern: Clear

~~It appears that I~~In this study, there is an observed inverse relationship between the age of sick children and adherence to IMCI guidelines. ~~Specifically, the finding suggests that as the age of the child increases, the odds of adherence to IMCI decrease.~~ This finding is consistent with study done using four African countries' SPA datasets(23). Research conducted in Tanzania also suggests a possible bias towards younger children(26). The study found that healthcare providers dedicated more assessment time to children under 5 years old compared to older children. ~~This might be because healthcare providers spent more time assessing younger children than older ones~~(26). Another reason might be due to work overload(27, 28) or that healthcare providers are less likely to stick to IMCI recommendations for older children, possibly due to assumptions that older children are more resilient to illness or perceived conditions as less severe. Not sticking to the recommendations of the IMCI guideline during assessment of three main sickness in older children (≥24 months) could lead to suboptimal health outcomes like misclassification of diseases, increased costs, additional complications, and prolonged hospital stays.~~Not sticking to the recommendation of IMCI guideline during assessment of fever, diarrhea or difficulty of breathing in older children is (≥24 months) could~~

~~result in suboptimal health outcomes, misclassification of diseases, increased cost, further complications, and long stay in hospital.~~ The health governing body in the country should focus on the need for enhanced training and capacity building for healthcare professionals to ensure that they are equipped with the knowledge and skills required to appropriately manage childhood illnesses in older children.

Another finding of this study ~~is~~ was children living in rural settings ~~of Ethiopia~~ face ~~Rural~~ heightened challenges when it comes to adhering to the IMCI guidelines~~,~~ ~~particularly~~ in comparison to their urban counterparts. Similar finding is also reported ~~by~~ in another study done in the united states of America (29) which concluded that rural dwellers are less likely to get preventive health services based on recommended healthcare guidelines. This might be due to that rural-urban divide of skilled healthcare workers. ~~In general, rural healthcare settings face a shortage of skilled health professionals in general~~(30, 31) ~~and more skilled health professionals are concentrated in urban areas than rural settings in many sub-Saharan countries~~(32)~~.~~ A key challenge in rural healthcare settings in many sub-Saharan countries, as documented evidence shows, is the shortage of skilled health professionals(30, 31). These professionals are concentrated in urban areas compared to rural settings(32). This lack of staff in rural settings might lead healthcare workers to spend less time assessing older children, hindering strict adherence to IMCI for them. Another justification for this finding might be the lower levels of education in rural areas caregivers (12, 33) may contribute to a lack of awareness and parents' ability to recognize and respond to signs of illness in their children. Addressing education, community awareness, closing the gap of skilled health professional distribution in rural areas related challenges could contribute to narrowing the gap in adherence

to IMCI guidelines between rural and urban populations, ultimately improving the health outcomes of children in rural areas.

Regarding the qualification, health officers (HOs) are more likely to adhere to IMCI protocol when assessing children compared to nurses. ~~From care provider related factors, being a professional qualified in health officer (HO) is found to have an increased likelihood of following the recommendation of IMCI protocols during assessing children who come with complaints of fever, difficulty of breathing or diarrhea compared to nurse professionals.~~ One possible reason for this could be that health officer professionals receive more specialized education on IMCI protocols during their professional training at university compared to nurse professionals. ~~A possible explanation for this might be Health officers typically undergo specific education on IMCI protocols during their professional development at university than nurse professionals.~~ According to the Ethiopian curriculum, health officers have a wider scope of practices related to clinical skills, including the comprehensive assessment and management of childhood illnesses(34).

Finally, the facility level factors revealed a significant difference in the likelihood of children receiving clinical services in accordance with IMCI recommendations based on the type of ~~healthcare~~ facility visited. Specifically, children who had sought care in health centers demonstrated a notably higher likelihood, nearly 2.61 times more, of receiving clinical services aligned with IMCI guidelines compared to their counterparts who were attended to in private health facilities. This finding is consistent with other studies done in other African countries such in Uganda(35), north-west Ethiopia(36), Nigeria(37, 38), western Kenya (39) which reported that the provision of health services in accordance with their national guidelines is unsatisfactory. It seems possible that these results are due to the major gap of the country in

Formatted: Font: (Default) Times New Roman, Font color: Auto, Pattern: Clear

regulating the health workers in private health facilities (40). Other alternative explanations for this result ~~are~~ might be that private health facilities may face resource constraints, limiting their ability to invest in staff training, and other resources required for implementation of IMCI recommendations. This finding suggests that targeted intervention specifically to private health facilities is required to ensure that children, regardless of the type of ~~healthcare~~ facility, receive clinical services in accordance with IMCI recommendations.

Strengths and limitation of the study

Finally, several strengths and limitations can be pinpointed from this study. The main strength of this study ~~is~~ was the utilization of most recent national data to study adherence of health workers to IMCI guideline. ~~The authors of~~ T~~t~~his study explored the clustering of health professionals reluctant to adhere to IMCI guideline using the emerging statistical analysis called spatial analysis in the context of health systems. Location specific response to health problems based on identified clusters could help in narrowing health inequalities.

Formatted: Indent: Left: 0.25"

The national survey data ~~utilized~~ used in this study omits data of Tigray region. So, the exclusion of ~~Tigray region's data~~this region from this study might have limited the generalizability of findings to the entire population of the country. The opportunity to gain insight into the Tigray region about prevalence, contributing factors and hotspot areas of adherence to IMCI protocols are missed and this could impair region-specific policy recommendations. In relation to the cross-sectional aspect, SPA Ethiopia encounters analogous constraints to other cross-sectional surveys i.e., cannot be used to determine the cause of something. Hence, we used data from secondary survey, missing other behavioral and cultural factors in this study could compromise model performance. Moreover, ~~.~~this study didn't

directly measure healthcare professionals' adherence to established protocols for assessing danger signs and conducting physical examinations in children. While the data hints at areas for improvement, it can't decisively determine overall adherence rates or reasons for deviations from protocols.

This study's observational design introduces other limitations. The Hawthorne effect, where healthcare providers modify their behavior when aware of observation, could lead to an overestimation adherence rate to IMCI protocols. Furthermore, our dependence on caregiver recall of symptoms through questioning presents a potential source of bias. Inaccurate or incomplete recall by caregivers might distort the data, hindering a precise assessment of actual adherence practices.

Conclusion

The rate of adherence to IMCI guideline in Ethiopia is low. Low adherence could have various implications, including potential gaps in the quality of child healthcare and the prevention and management of childhood illnesses. The study identified child's age, types of facility, academic qualification and place of residency as crucial factors correlated with adherence rate. By tailoring interventions to the unique needs of each geographic area, this targeted plan aims to create a more equitable distribution of health professionals adhering to IMCI protocols, ultimately improving child healthcare outcomes across the region. A full implementation of IMCI programs should consider several key factors to ensure comprehensive and effective coverage like the diverse needs and contexts of children, healthcare facilities, and providers.

Additional research is necessary to conduct thorough analyses in hotspot areas and to examine changes in IMCI adherence over time, exploring spatio-temporal relationships. It is advisable

Formatted: Indent: Left: 0.25"

to undertake studies comparing hotspot areas with non-hotspot regions to discern any noteworthy differences or similarities influencing variations in adherence. Furthermore, we suggest that future researchers employ qualitative research methods to gather local perspectives on IMCI adherence in hotspot areas, providing nuanced insights into community beliefs and attitudes.

Funding

The authors received no specific funding for this work.

Formatted: Indent: Left: 0.25"

Contributions

AAT, AK, NK, YT, AE, SDK, KMA, ET, EBE, CD, LA, FDB, MA and AM were involved in the design and conception of the study; the analysis, and interpretation of the findings. All the authors read and approved the manuscript starting from the first step up to this.

Abbreviations

AIC: Akaike information criteria, **a~~A~~OR**: Adjusted Odds Ratio, **BIC**: Bayesian information criteria**, ESPA**: Ethiopian Service Provision Assessment, **DIC**: Deviance Information Criterion, **ICC**: Intra-Class Correlation, **LLR**: Log-Likelihood Ratio, **PCV**: Proportion of Variance Change, **CI**: confidence interval, **COR**: Crude Odds Ratio~~, **AOR**: Adjusted odds ratio, ~~**EA**: enumeration areas, **SNNP**: South Nation and Nationalities People, **KM**: Kilometer, **IMCI**: Integrated Management Childhood Illness, **EPHI**: Ethiopian public health institute, MOH: Ministry of Health, **USAID**: United States Agency for International Development, TB: tuberculosis, **NCD:** Non-Communicable Diseases**, NTD:** Neglected Tropical Diseases, **VIF**:

variance inflation factors, **HO**: health officer, **MD**: Medical Doctor, **HF**: health facility, **HC**: health center**, ID/EA**: cluster ID/ enumeration areas, **IQR**: interquartile range.

Ethics approval and consent to participate

As the authors utilized secondary data from 2021-SPA Ethiopia, consent of participants and ethical approval is not required. But the authors have been granted access to the dataset of 2021 SPA-Ethiopia through registering to https://www.dhsprogram.com/ websites.

Consent for publication

Not applicable

Availability of data and material

All data used to conduct this study are provided within the manuscript.~~The datasets and materials are available at https://www.dhsprogram.com. The minimal data used for this study are available from the corresponding author on reasonable request~~.

Competing interests

The authors declares that he has no competing interests

Reference

1. Ministry of Health - MoH/Malawi, ICF International. Malawi Service Provision Assessment 2013-14. Lilongwe, Malawi: MoH/Malawi and ICF International; 2014.

Formatted: Normal

2. Ethiopian Public Health Institute, Ethiopia Ministry of Health, ICF. Ethiopia Service Provision Assessment 2021–22. Addis Ababa, Ethiopia, and Rockville, Maryland, USA: EPHI, MoH and ICF; 2023.

3. Bryce J, Victora CG, Habicht J-P, Black RE, Scherpbier RWJHp, planning. Programmatic pathways to child survival: results of a multi-country evaluation of Integrated Management of Childhood Illness. 2005;20(suppl_1):i5-i17.

4. Black RE, Morris SS, Bryce JJTl. Where and why are 10 million children dying every year? 2003;361(9376):2226-34.

5. Lulseged SJEmj. Integrated management of childhood illness: a review of the Ethiopian experience and prospects for child health. 2002;40(2):187-201.

6. Gove SJBotWHO. Integrated management of childhood illness by outpatient health workers: technical basis and overview. The WHO Working Group on Guidelines for Integrated Management of the Sick Child. 1997;75(Suppl 1):7.

7. Nguyen DTK, Leung KK, McIntyre L, Ghali WA, Sauve RJPo. Does integrated management of childhood illness (IMCI) training improve the skills of health workers? A systematic review and meta-analysis. 2013;8(6):e66030.

8. Abayneh M, Gebremedhin T, Dellie E, Tsehay CT, Atnafu AJIJoP. Improving the Assessment and Classification of Sick Children According to the Integrated Management of Childhood Illness (IMCI) Protocol at Sanja Primary Hospital, Northwest Ethiopia: A Pre-Post Interventional Study. 2020;2020.

9. Assefa Y, Gelaw YA, Hill PS, Taye BW, Van Damme WJG, health. Community health extension program of Ethiopia, 2003–2018: successes and challenges toward universal coverage for primary healthcare services. 2019;15:1-11.

10. Robinson DJAh. The integrated management of childhood illness. 1996;18(6):20-1.

11. Federal Ministry of Health Ethiopia %J FMOH AA, Ethiopia. Health Sector transformation plan, 2015/16-2019/20. 2015.

12. Central Statistical Agency - CSA/Ethiopia, ICF. Ethiopia Demographic and Health Survey 2016. Addis Ababa, Ethiopia: CSA and ICF; 2017.

13. Snow JJAA. Health facility End-line Survey-synthesis report. 2008.

14. Abraha A, Myléus A, Byass P, Kahsay A, Kinsman JJPO. Social determinants of under-5 child health: A qualitative study in Wolkayit Woreda, Tigray Region, Ethiopia. 2019;14(6):e0218101.

15. Ababa FDRoEMoHJMoHA. Health Sector Development Program IV 2010/11-2014/15. 2010.

16. Sheng J, Han X, Zhou H. Spatially varying patterns of afforestation/reforestation and socio-economic factors in China: a geographically weighted regression approach. Journal of Cleaner Production. 2017;153:362-71.

17. Midi H, Sarkar SK, Rana S. Collinearity diagnostics of binary logistic regression model. Journal of Interdisciplinary Mathematics. 2010;13(3):253-67.

18. StataCorp L. Stata statistical software: release 14. Statacorp lp College Station, TX; 2015.

19. Merlo J, Chaix B, Yang M, Lynch J, Råstam LJJoE, Health C. A brief conceptual tutorial of multilevel analysis in social epidemiology: linking the statistical concept of clustering to the idea of contextual phenomenon. 2005;59(6):443-9.

20. Merlo J, Chaix B, Ohlsson H, Beckman A, Johnell K, Hjerpe P, et al. A brief conceptual tutorial of multilevel analysis in social epidemiology: using measures of clustering in multilevel logistic regression to investigate contextual phenomena. 2006;60(4):290-7.

21. Vrieze SIJPm. Model selection and psychological theory: a discussion of the differences between the Akaike information criterion (AIC) and the Bayesian information criterion (BIC). 2012;17(2):228.

22. Lange S, Mwisongo A, Mæstad OJSs, medicine. Why don't clinicians adhere more consistently to guidelines for the Integrated Management of Childhood Illness (IMCI)? 2014;104:56-63.

23. Krüger C, Heinzel-Gutenbrunner M, Ali MJBHSR. Adherence to the integrated management of childhood illness guidelines in Namibia, Kenya, Tanzania and Uganda: evidence from the national service provision assessment surveys. 2017;17(1):1-12.

24. Abtew S, Negatou M, Wondie T, Tadesse Y, Alemayehu WA, Tsegaye DA, et al. Poor adherence to the integrated community case management of newborn and child illness protocol in rural Ethiopia. 2022;107(6):1337.

25. Reñosa MD, Dalglish S, Bärnighausen K, McMahon SJGHA. Key challenges of health care workers in implementing the integrated management of childhood illnesses (IMCI) program: a scoping review. 2020;13(1):1732669.

26. Adam T, Manzi F, Schellenberg JA, Mgalula L, de Savigny D, Evans DBJBotWHO. Does the Integrated Management of Childhood Illness cost more than routine care? Results from the United Republic of Tanzania. 2005;83(5):369-77.

27. Adam T, Amorim DG, Edwards SJ, Amaral J, Evans DBJHp, planning. Capacity constraints to the adoption of new interventions: consultation time and the Integrated Management of Childhood Illness in Brazil. 2005;20(suppl_1):i49-i57.

28. Edward A, Dam K, Chege J, Ghee AE, Zare H, Chhorvann CJIJfQiHC. Measuring pediatric quality of care in rural clinics—a multi-country assessment—Cambodia, Guatemala, Zambia and Kenya. 2016;28(5):586-93.

29. Casey MM, Call KT, Klingner JMJAjopm. Are rural residents less likely to obtain recommended preventive healthcare services? 2001;21(3):182-8.

30. Watch BJTSoHiB. Health Workforce in Bangladesh: Who Constitutes the Healthcare System. 2007.

31. Ahmed SM, Hossain MA, RajaChowdhury AM, Bhuiya AUJHrfh. The health workforce crisis in Bangladesh: shortage, inappropriate skill-mix and inequitable distribution. 2011;9(1):1-7.

32. Anyangwe SC, Mtonga CJIjoer, health p. Inequities in the global health workforce: the greatest impediment to health in sub-Saharan Africa. 2007;4(2):93-100.

33. Zhang YJCer. Urban-rural literacy gaps in Sub-Saharan Africa: The roles of socioeconomic status and school quality. 2006;50(4):581-602.

34. Negash FJSIJ. Health Officer: A Physician Assistant Comparable Role in Ethiopia. 2021;8.

35. Mpimbaza A, Babikako H, Rutazanna D, Karamagi C, Ndeezi G, Katahoire A, et al. Adherence to malaria management guidelines by health care workers in the Busoga sub-region, eastern Uganda. 2022;21(1):1-15.

36. Argaw MD, Mavundla TR, Gidebo KDJBHSR. Management of uncomplicated malaria in private health facilities in North-West Ethiopia: a clinical audit of current practices. 2019;19(1):1-14.

37. Mokuolu OA, Ntadom GN, Ajumobi OO, Alero RA, Wammanda RD, Adedoyin OT, et al. Status of the use and compliance with malaria rapid diagnostic tests in formal private health facilities in Nigeria. 2016;15(1):1-11.

38. Udoh E, Oyo-Ita A, Odey F, Effa E, Esu E, Oduwole O, et al. Management of uncomplicated malaria in underfives in private and public health facilities in South-eastern Nigeria: a clinical audit of current practices. 2013;2013.

39. Riley C, Dellicour S, Ouma P, Kioko U, ter Kuile FO, Omar A, et al. Knowledge and adherence to the national guidelines for malaria case management in pregnancy among healthcare providers and drug outlet dispensers in rural, western Kenya. 2016;11(1):e0145616.

40. Dejene D, Yigzaw T, Mengistu S, Ayalew F, Kahsaye M, Woldemariam DJGHR, et al. Exploring health workforce regulation practices and gaps in Ethiopia: a national cross-sectional study. 2019;4(1):1-12.

Formatted: Indent: Left: 0.25"

Authors' response to reviews

Title: Assessing Health Professionals' Adherence to IMCI Protocols for Key Symptoms in Sick Children in Ethiopia: Findings from the 2021-2022 National Service Provision Survey:

Corresponding author: Abiyu Abadi Tareke (abiyu20010@gmail.com)

Version: 2

Date: May 14, 2024

Point by point response for editors/reviewers' comments

Manuscript number: PONE-D-24-03866

Dear editor/reviewer:

Dear all,

We express our profound appreciation for the insightful and productive feedback that you have provided. Your invaluable comments have significantly enriched the quality of the manuscript, and have greatly augmented our expertise in the realm of scientific paper writing. The authors have diligently considered each of the comments and queries raised by the editors and reviewers, and have responded to them in a targeted manner. Our comprehensive point-by-point rejoinders to all the comments and questions can be found in the subsequent pages. In addition, an accompanying supplementary document has been enclosed, which showcases the modifications made in detail, using the track changes feature. We also made some change to fix grammatical error in some paragraphs.

Review Comments to the Author

Reviewer #1: Kindly provide details of the outcome variable how was this assessed and how the decision was made regarding the non-adherent workers. I've attached a marked PDF with comments for your reference.

Authors' Response: Thank you for your feedback. Thank you for your valuable feedback and the attached marked PDF with comments. We appreciate your request for clarification on the outcome variable and assessment methods.

Outcome Variable: Adherence to IMCI Protocols

We defined adherence to IMCI protocols as the complete assessment of three key symptoms in sick children: fever, cough/difficulty breathing, and diarrhea. This aligns with the core components of the IMCI approach for identifying and managing common childhood illnesses.

Adherence was assessed through **direct observation** of sick child consultations by trained survey interviewers. The interviewers used standardized **observation checklists** specifically designed to capture adherence to IMCI guidelines for these three key symptoms. But this observational doesn't include physical examination checklists. Checklists included items about history of three main symptoms for any sick child:

- Did the child have difficulty of breathing or cough?
- Did the child have diarrhea?
- Did the child have fever?

Non-Adherence:

A health professional was classified as non-adherent if they did not perform a specific procedure recommended by the IMCI guidelines for assessing one or more of the three key symptoms. The specific criteria for non-adherence were outlined in the observation checklist. But interviewer did not assess whether the information shared was correct or whether findings were interpreted appropriately.

Reviewer #2: Dear author(s), thank you for the opportunity to review your article titled "Adherence of Health Professionals to National IMCI Protocols: A Study on Assessing Three Key Symptoms in Sick Children in Ethiopia - Insights from the 2021 National Service Provision Survey". Overall, I find your article very interesting and relevant for the journal. However, there are certain points which you can work on to further improve your manuscript:

Authors' Response: Thank you**.** Absolutely! We are happy to hear your feedback and suggestions for improvement.

1. Grammar/language: during review, I noticed many typos and certain sentences that would need to be rephrased. In line 142, it is "Health care workers" while in line 144, it is "Healthcare workers".

Authors' response: Thank you for bringing these issues to my attention. We apologize for the typos and inconsistencies in the sentences. We carefully reviewed the document again and made the necessary corrections. We appreciate your feedback and attention to detail.

Line 161, "caretaker" replace it with "caregiver".

Authors' response: Thank you for pointing out the error. We changed in line 161 from "caretaker" to "caregiver" to ensure accuracy. Your attention to detail is greatly appreciated.

Line 247, "Therefore, additional consent is not be necessary for this study". Line 270, "…toward IMCI guideline is found is higher during…". Line 367, "… difficulty of breathing in older children is (≥24 months) could result…". Line 378, "In general, rural healthcare settings face a shortage of skilled health professionals in general(26, 27)…" There is repetition of 'in general'. All of these sentences need to be rephrased. Please pay attention to upper and lower case letters to use them appropriately, particularly in the titles of the tables and figures. Additionally, a thorough review from a language expert is recommended for further corrections.

Authors' Response: Thank you for bringing these issues to our attention. We apologize for the errors and repetition in those sentences. Here are the revised versions:

Line 247: "Therefore, additional consent is not required for this study."

Line 270: " The adherence rate of health workers toward the IMCI guideline is higher during the assessment of fever, specifically at 70%. Adherence is higher, specifically 36%, when assessing children aged ≤ 24 months compared to those aged ≥ 24 months. Furthermore, a high percentage of adherence, specifically 44%, was observed when children were assessed by health officers. Similarly, children seen at health centers showed an adherence rate of 42%. "

Line 367: " Not sticking to the recommendations of the IMCI guideline during assessment of three main sickness in older children (≥24 months) could lead to suboptimal health

outcomes like misclassification of diseases, increased costs, additional complications, and prolonged hospital stays."

Line 378: "A key challenge for rural healthcare settings in many sub-Saharan countries, as documented evidence shows, is the shortage of skilled health professionals. These professionals are concentrated in urban areas compared to rural settings."

We also ensure proper capitalization, especially in the titles of tables and figures. We appreciate your feedback and recommend a thorough review by a language expert for further corrections. We invite university of Gondar English teachers to edit and comment on our manuscript.

2. Abstract: “BIC and AIC”? always write a full form of non-standard abbreviations. “…survey conducted in 2021.”, write down the duration of the survey as you did later in the Methods and Materials section.

Authors’ Response: Thank you for your feedback. We apologize for the oversight. The survey was conducted over a period of seven months in 2021. We will make sure to include the duration of the survey in the Abstract, as we have done in the Methods and Materials section. We also apologize for not providing the full form of the abbreviations "BIC" and "AIC" in the abstract. In the revised version, we ensured including the full form of these non-standard abbreviations, stating "Bayesian Information Criterion (BIC)" and "Akaike Information Criterion (AIC)" in the abstract.

3. Introduction: In abbreviation to E-SPA, you've written “Ethiopian health service provision”. Assessment word is missed. Para 6, “This study is the first study…”, rephrase this sentence.

Authors’ Response: In abbreviation to E-SPA (Ethiopian Health Service Provision Assessment), the word "Assessment" was inadvertently missed. We apologize for the error. In the revised version, we will make sure to include the complete abbreviation as "Ethiopian Health Service Provision Assessment (E-SPA)". Thank you for bringing this to our attention, and we appreciate your attention to detail.

Para 6, “This study is the first study…”, rephrase this sentence.

Authors’ Response: Thank you for your comment. The sentence is re-phased as **“**This study is the first to assess healthcare worker adherence to IMCI guidelines for evaluating three key childhood illness symptoms.”

4. Methods: Line 125, provide a reference for the health service-delivery structure for the readers to understand the context of it. Define your health facility types here which is used a lot in your research. Considering the secondary nature of your data, mention whether ethics and consent were taken into consideration in the original survey. Follow strobe or other relevant guidelines to arrange your article.

Authors’ Response: We have now included a reference in the introduction to provide readers with a comprehensive understanding of the health service-delivery structure. This addition will help contextualize our study within the broader healthcare framework. Given the secondary nature of our data, we have addressed the ethical considerations by explicitly mentioning that the original survey, from which our data were derived, adhered to ethical

standards and obtained appropriate consent from participants. We have clarified this point to ensure transparency regarding the ethical procedures followed during the original survey. We included your comments under method section >> Ethical consideration section.

5. Results: Start with the whole survey demographics then the population of interest or only the later one I.e. for 788 patients. Here you have just included the overall participants' demographics of the original survey. Follow one format for writing confidence intervals.

Authors' Response: To enhance clarity, we will revise the presentation of demographics data to start with the overall survey demographics before focusing on the population of interest, i.e., the 788 patients included in our study. By following this format, readers will have a clearer understanding of the broader demographic characteristics of the survey participants before delving into the specifics of our study population. Dear reviewers thank you for your suggestions. In the initial submission of this manuscript, Table 1 was inadvertently included before removing missing values from the dependent variable, resulting in an inflated sample size of 3641 instead of the correct 788. To rectify this error, we have replaced the values in columns 2 and 3 by copying values from the accurate table generated after removing missing values using the Stata command. We apologize for this oversight and appreciate the valuable feedback from reviewer 1 and reviewer 2, which helped us identify and correct this error.

Here you write it as "…with a 95% confidence interval ranging from 29.7% to 36.3%" while in another place you mention it as "(95% CI: 29.70%, 36.26%)".

Authors' Response: Thank you for pointing out the inconsistency. To ensure uniformity in reporting confidence intervals throughout the manuscript, we will adopt one format for presenting them. Considering both options provided, we suggest using the format "[95% CI: 29.7%, 36.3%]" for consistency. This format is concise and widely recognized, making it easier for readers to interpret the confidence intervals across different sections of the manuscript. We appreciate your attention to detail and will make the necessary revisions accordingly.

Line 271, age is written as "≤ 24 months" while in tables it is 2-24 months. This changes the meaning of it. Only follow 2-24 months.

Authors' Response: Thank you for your feedback. Here's the revised paragraph and adjustment to the age representation: We standardized the age representation to "2-24 months" vs "≥24 months" throughout the manuscript to maintain consistency and clarity.

Line 270-273, rewrite this whole para in correct order. Additionally, here, when a no. is greater among all it's called "highest" not higher.

Authors' Response: dear reviewer for your surprise this paragraph is also recommended to rephrase by the other peer reviewer. Thank you for your feedback and the additional clarification regarding the rephrasing recommendation by the other peer reviewer. Here's the revised paragraph with the correct ordering and terminology:

"*The adherence rate of health workers toward the IMCI guideline is highest during the assessment of fever, specifically at 70%. Adherence is higher, specifically 36%, when assessing children aged 2-24 months compared to those aged ≥ 24 months. Furthermore, a highest percentage of adherence, specifically 44%, was observed when children were*

assessed by health officers. Similarly, children seen at health centers showed an adherence rate of 42%."

We appreciate the opportunity to address these concerns and ensure the clarity and accuracy of our manuscript. If there are any further adjustments needed, please let us know.

Line 305, "… AIC (Akaike Information Criterion)…" is written as Akaike Information Criterion (AIC). Line 313, "…child being over 24 months". In other places you mention it as "age >24 months", maintain consistency in your manuscript.

Authors' Response: Line 305: We adjusted the text to maintain consistency, writing "AIC (Akaike Information Criterion)" as "Akaike Information Criterion (AIC)" throughout the manuscript. Additionally, line 313: We modified the expression "child being over 24 months" to "age ≥24 months" to align with the terminology used elsewhere in the manuscript.

6. Discussion: Since general danger signs were not considered in the study, mention them in your limitations. Para 3 and 4 lack sufficient comparisons to past studies. Provide a critical analysis of your findings with the prior studies for the mentioned paragraphs.

Authors' Response: We agree that paragraphs 3 and 4 could benefit from a more in-depth comparison with past studies. We made the necessary revisions.

Line 359, "It appears that in this study…" rephrase it simply to "In this study…". First 2 sentences of the following para have the same meanings, avoid repetition and keep it concise. Line 405, isn't it counterintuitive that private sectors have resource constraints? Justify your explanation in the text.

Authors' Response: Thank you for your feedback. Here's how we addressed your suggestions: Line 359: We simplified the expression to "In this study..." for conciseness and clarity. Following paragraph: We consolidated the first two sentences to avoid repetition and keep it concise while conveying the same meaning effectively.

7. Tables and Figures: Tables 1 and 4 are missing footnotes. Mention in it all the abbreviations included in the respective tables. An ideal table should stand for itself without referring to text. Table 2, avoid phrases like "all 3 symptoms in one", write it instead as "all 3 symptoms included". Again age here is ≤24 months. Clear this controversy. Use single terms for a variable across the tables. E.g. here you name it "Facility type" while in Table 5 it is "Health facility type". In Table 3, the p-values are written inappropriately. E.g one is "0.00000027" other is "0.0027". Round them off to the same number of digits. Table no.5, avoid writing the p-value as "0.000" instead write it as p <0.001. Adjusted odds ratios are usually written as aOR not as "AOR".

Authors' Response: Thank you for the detailed feedback on the tables. Here's how we'll address each point: According to your recommendations, In **Tables 1 and 4,** we included footnotes mentioning all the abbreviations used in the respective tables to enhance clarity and ensure that the tables standalone without referring to the text. **Table 2:** We modified the phrase "**all 3 symptoms in one"** to "**all 3 symptoms included**" accordingly. Regarding the age controversy, we standardized the representation to "<24 months" across all tables and narrations for consistency. **Table 3:** We round off the p-values to the same number of digits (to four decimal places) for consistency purpose**. Table 5:** We will adjust the presentation of p-values as "p <0.001" instead of "0.000". throughout our manuscript toughly ensured consistency in variables naming. We decided to continue with "facility

type” instead of “types of health facility or health facility type”. Finally, Adjusted odds ratios will be presented as "aOR" instead of "AOR" in Table 5 and other part of the manuscript to maintain consistency with standard conventions.
