# Supplementary material for: Adherence to IMCI guidelines for key symptoms in Ethiopian children: A 2021–2022 national service provision survey
Source: PLoS One. 2024 Oct 16;19(10):e0312138. doi: 10.1371/journal.pone.0312138 (PMC11482708; doi:10.1371/journal.pone.0312138)
Supplement: Attachment — Submitted filename: Response to Reviewers.docx [file pone.0312138.s004.docx]

**Authors’ response to reviews**

Title: Adherence to IMCI Guidelines for Key Symptoms in Ethiopian Children: A 2021-2022 National Service Provision Survey

Corresponding author: Abiyu Abadi Tareke ([abiyu20010@gmail.com](mailto:abiyu20010@gmail.com))

Version: 2

**Date:**  May 15, 2024

Point by point response for editors/reviewers’ comments

Manuscript number: PONE-D-24-03866

Dear editor/reviewer:

Dear all,

We express our profound appreciation for the insightful and productive feedback that you have provided. Your invaluable comments have significantly enriched the quality of the manuscript, and have greatly augmented our expertise in the realm of scientific paper writing. The authors have diligently considered each of the comments and queries raised by the editors and reviewers, and have responded to them in a targeted manner. Our comprehensive point-by-point rejoinders to all the comments and questions can be found in the subsequent pages. In addition, an accompanying supplementary document has been enclosed, which showcases the modifications made in detail, using the track changes feature. We also made some change to fix grammatical error in some paragraphs.

**Review Comments to the Author**

**Reviewer #1:** Thank you for the revision and clarification of the terms which were not clear in the previous version. However, there are still issues which were highlighted and are not addressed in the revision. I am attaching the revised paper with highlighted text and comments for you to address. Additionally, the manuscript needs proof reading and correction from a language expert. There are still many grammatical errors and issues.

**Authors’ response:** Thank you for the thorough review of our revised manuscript. We appreciate you taking the time to provide detailed feedback and highlighting the areas that still need improvement. We have carefully reviewed the comments and tracked changes you provided in the attached document. We addressed each of the remaining issues and concerns you have raised. Regarding the language and grammar, we will have the manuscript proofread by a professional language editor to correct any remaining grammatical errors and improve the overall writing quality. We are committed to producing a high-quality manuscript that meets the standards of your journal. Thank you again for your valuable input.
