# Supplementary material for: Adherence to IMCI guidelines for key symptoms in Ethiopian children: A 2021–2022 national service provision survey
Source: PLoS One. 2024 Oct 16;19(10):e0312138. doi: 10.1371/journal.pone.0312138 (PMC11482708; doi:10.1371/journal.pone.0312138)
Supplement: Attachment — Submitted filename: Response to Reviewers.docx [file pone.0312138.s005.docx]

**Authors’ response to reviews**

Title: Adherence to IMCI Guidelines for Key Symptoms in Ethiopian Children: A 2021-2022 National Service Provision Survey

Corresponding author: Abiyu Abadi Tareke ([abiyu20010@gmail.com](mailto:abiyu20010@gmail.com))

Version: 3

**Date:**  September 14, 2024

Point by point response for editors/reviewers’ comments

Manuscript number: PONE-D-24-03866

Dear editor/reviewer:

Dear all,

We sincerely appreciate the insightful and constructive feedback you have provided. Your invaluable comments have significantly enhanced the quality of the manuscript and deepened our understanding of scientific writing. The authors have carefully considered each comment and question raised by the editors and reviewers, responding to them in a focused manner. Our detailed point-by-point responses to all feedback can be found in the following pages. Additionally, we have included a supplementary document that highlights the modifications made, utilizing the track changes feature. We also corrected some grammatical errors in a few paragraphs.

**Editors comment to Authors**

 We note that Figures 1 and 3 in your submission contain [map/satellite] images which may be copyrighted. All PLOS content is published under the Creative Commons Attribution License (CC BY 4.0), which means that the manuscript, images, and Supporting Information files will be freely available online, and any third party is permitted to access, download, copy, distribute, and use these materials in any way, even commercially, with proper attribution.

**Answer=** Dear editors,

Thank you for your feedback regarding the images used in Figures 1 and 3 of our manuscript. We would like to clarify that these images were generated by us using ArcGIS and StatsCan software, based on our own analyses.

The shapefile used for the preparation of the maps was sourced from the Open-Source website OCHA Ethiopia (<https://data.humdata.org/organization/ocha-ethiopia>). We have ensured that this data is publicly available and adheres to the necessary licensing requirements.

We affirm that we hold the rights to the images we created, and we have documented our methods and data sources in the manuscript to provide clarity.

**Review Comments to the Authors**

**Reviewer #1:** Reviewer #1: I have reviewed the paper but some of the comments are not incorporated or answered. Therefore, I would like to request to kindly provide point by point response on each of the comment raised in R1 file. Also, the same should be evident in the track change file for easy follow up.

**Authors’ response:** Dear Reviewer #1, Thank you for your thorough review of our paper. We appreciate your feedback and understand the importance of addressing all your comments. We have carefully reviewed each point you raised in the R1 file and have provided a detailed point-by-point response.

In addition, we have made sure that these responses are reflected in the track change document for your convenience. Please find both the responses and the updated manuscript attached for your review.

Thank you once again for your valuable insights

**Abstract**

**Method=** The sentence “The data utilized to analyze this study was based on information collected through 33 Service provision assessment in Ethiopia (SPA-ET) survey conducted in 2021.” Is rewrote as “*The data for this study were gathered from the Service Provision Assessment (SPA) survey in Ethiopia, which was conducted nationwide from August 11, 2021, to February 4, 2022*”

**Result= the** variable name “health facility type” is changed to “facility type (health center)”, and *child’s aged* is changed to *“child’s age (being ≥24 months)”*

**Method**

**Study area= the “***pharmacies, and drug stores”* is modified as *“pharmacies”* because of in Ethiopia context pharmacies and drug stores have similar meaning.

**Dependent variable=** the sentence that you highlighted was controversial and unclear. This controversy is also detected and commented by reviewer two. Following your valuable comments, we modified the whole paragraph and see the revised version of the manuscript. This also works for **sampling procedure** sections.

**Result**

The sentence; “A total of 788 sick children who were assessed for at least one the main three symptoms of childhood illness (fever, cough and diarrhea) were included in this study.” Is modified as “From the total of 788 health professionals who assessed sick children, 300 (38 %) were from the Oromia region, followed by SNNP 212 (27%) (Table 1).”

**Discussion**

**The word install is remodified as *“****We recommend policy makers and program implementers to* ***integrate*** *evidence-based policy recommendations to address systemic issues contributing to non-adherence.***”**
